# Nanocollagen for Historical Leather Conservation: A Case Study on Three Islamic Bookbindings

**DOI:** 10.3390/s26216709

**Published:** 2026-10-23

**Authors:** Irene Angela Colasanti, Federica Valentini, Alberto Mario Campus, Stefania Zeppieri, Camilla Zaratti, Andrea Macchia, Michela Relucenti, Loredana Cristiano, Ilaria Fratoddi, Sara Cerra

**Affiliations:** 1 Sciences and Chemical Technologies Department, Tor Vergata University, Via della Ricerca Scientifica 1, 00133 Rome, Italy; ireneangela.colasanti@students.uniroma2.eu (I.A.C.); albertomario.campus@alumni.uniroma2.eu (A.M.C.); camilla.zaratti@students.uniroma2.eu (C.Z.); 2 Youth in Conservation of Cultural Heritage Aps, Via Torquato Tasso 108, 00185 Rome, Italy; aps@yococu.com; 3 Department of Anatomical, Histological, Forensic and Orthopedic Sciences, Sapienza University of Rome, Via Alfonso Borelli 50, 00161 Rome, Italy; michela.relucenti@uniroma1.it (M.R.); loredana.cristiano@uniroma1.it (L.C.); 4 Department of Chemistry, Sapienza University of Rome, Piazzale Aldo Moro 5, 00185 Rome, Italy; ilaria.fratoddi@uniroma1.it (I.F.); sara.cerra@uniroma1.it (S.C.)

**Keywords:** Nanocollagen, historical leather conservation, analytical validation with laboratory and original samples, library of the University of Naples “L’Orientale”

## Abstract

Historical book artefacts are highly susceptible to degradation processes, particularly when characterised by leather bindings, whose deterioration progressively compromises the cohesion and structural integrity of the artefact. Among the consolidating agents commonly employed for their conservation, Klucel G is one of the most widely used materials; however, its long-term stability and compatibility with degraded leather substrates are still discussed. Consequently, alternative materials have been investigated for the conservation of historical leather, among which Nanocollagen has emerged as a particularly promising solution. The present study evaluates the performance of Nanocollagen compared to that exhibited by the conventional Klucel G-based treatments. Three Islamic bookbindings preserved at the Library of the University of Naples “L’Orientale” were characterised using a multi-analytical approach including optical microscopy, pH measurements, qualitative chemical assay, FTIR and Raman spectroscopy, SEM-EDS, Spectrocolorimetry, and Mechanical tests. Laboratory mock-up samples were prepared to assess the effectiveness of the treatments, and subsequently applied to the original samples collected on the historical bindings. The results demonstrated that Nanocollagen preserves the surface morphology of leather, induces negligible chromatic variations, and improves the flexibility of degraded samples when compared to the conventional Klucel G conservation treatments. The best result is observed when Nanocollagen is applied to samples exhibiting moderate damage—specifically, a structural order-to-disorder transition of the protein material (essentially, an early-stage rather than advanced case of “red rot”).

## 1. Introduction

Manuscripts and the indoor archival holdings represent a fundamental testimony to the transmission of knowledge, culture, and artistic traditions of Past Civilisations, providing valuable information on historical materials, manufacturing techniques, and cultural contexts. This was particularly evident in Islamic culture, where the book held not only a practical function but also a symbolic, cultural, and social value [1,2,3,4].

Book artefacts are generally composed of leather, parchment, paper, and inks [4,5]. Among these materials, leather is particularly vulnerable to physical, chemical, and biological degradation processes. Oxidation, hydrolysis, and denaturation of the collagen matrix can lead to hardening, embrittlement, colour alterations, and loss of fibre cohesion [6,7,8,9,10,11,12,13,14]. To mitigate these degradation phenomena and preserve the structural integrity of historical leather, consolidation represents one of the most widely adopted treatments. Its primary aim is to re-establish the cohesion of the degraded collagen matrix and limit further deterioration while maintaining the physical and aesthetic characteristics of the artefact [15,16]. This intervention is particularly important for leather affected by powdering, which compromises both conservation procedures and the safe handling and consultation of historical book artefacts [17,18,19,20,21].

Although numerous consolidating agents have been investigated, only a few provide an adequate balance between consolidating effectiveness, substrate compatibility, long-term stability, and preservation of the aesthetic characteristics of the materials [21]. Among these, the most widely used is Klucel G, a non-ionic cellulose derivative (hydroxypropyl cellulose, HPC). It is extensively employed in the conservation of bookbindings, particularly those affected by Red Rot, generally providing satisfactory results [20,21]. Klucel G can partially restore the flexibility of leather and is generally considered chromatically compatible with the substrate. However, over time it tends to become rigid, making the material more susceptible to mechanical stress. Furthermore, it is sensitive to sulphuric acid, a compound commonly associated with leather degradation and atmospheric pollution [20,21,22,23].

Despite the widespread use of Klucel G in leather conservation, concerns regarding its long-term stability have encouraged the search for innovative alternative materials capable of providing improved conservation performance. Yoo and Park (2024) [24] evaluated several consolidating formulations, but none achieved fully satisfactory results in terms of improving both flexibility and colour stability. Other studies have focused on SC6000, an emulsion of wax and acrylic resin in isopropanol, and on the red-rot cocktail, a mixture of SC6000 and Klucel G; however, these products impart a glossy appearance to the leather surface [16,20]. Ludwick (2013) [21] reported the use of Silicone Oils, a class of polymerised siloxanes characterised by different viscosities and physical–chemical properties, capable of providing greater substrate flexibility and improved stability under adverse environmental conditions. Although these products have shown certain specific advantages, they still present limitations related to substrate compatibility or alteration of the aesthetic characteristics of the treated artworks. For this reason, research in recent years has progressively shifted towards approaches based on materials that are chemically compatible with the original substrate [25,26,27,28].

In this context, Bicchieri, Valentini et al. (2018) [14] investigated the applicability of Nanocollagen to leather bookbindings, obtaining significant results in terms of enhanced mechanical strength and flexibility while reducing the fragility acquired by the artefact over time. The high affinity of Nanocollagen for the collagen matrix, combined with its nanoscale dimensions, promotes penetration into the substrate, enhances interactions with collagen fibrils, and favours the filling of interfibrillar voids, thereby improving the structural integrity of the materials. The treatment also provided satisfactory aesthetic results, with only limited colour variations [14]. Despite the promising applications of Nanocollagen on collagen-based artefacts, comparative studies specifically assessing its performance against Klucel G on historical objects remain limited.

According to this, the present study investigates the use of Nanocollagen and compares its consolidating features with those exhibited by conventional/traditional Klucel G. Both consolidating agents were applied for the conservation of three Islamic bookbindings preserved at the Library of the University of Naples “L’Orientale”. As the items consisted solely of binding fragments or detached bindings, it was not possible to identify their period of production. Based on the scientific investigations conducted in this study, the only ones available to date, the pieces show no signs of prior restoration. Consequently, this study is highly innovative, given the absence of authoritative sources documenting either the dating or any traces of earlier restoration and consolidation work.

A multi-analytical characterisation was carried out to identify the constituent materials, assess their conservation condition, and investigate the main degradation phenomena. Subsequently, the consolidating performance of Nanocollagen and Klucel G was compared through a series of analytical tests performed on both artificially aged laboratory samples and the original ones.

## 2. Materials and Methods

### 2.1. Sampling Procedures

**Figure 1:**
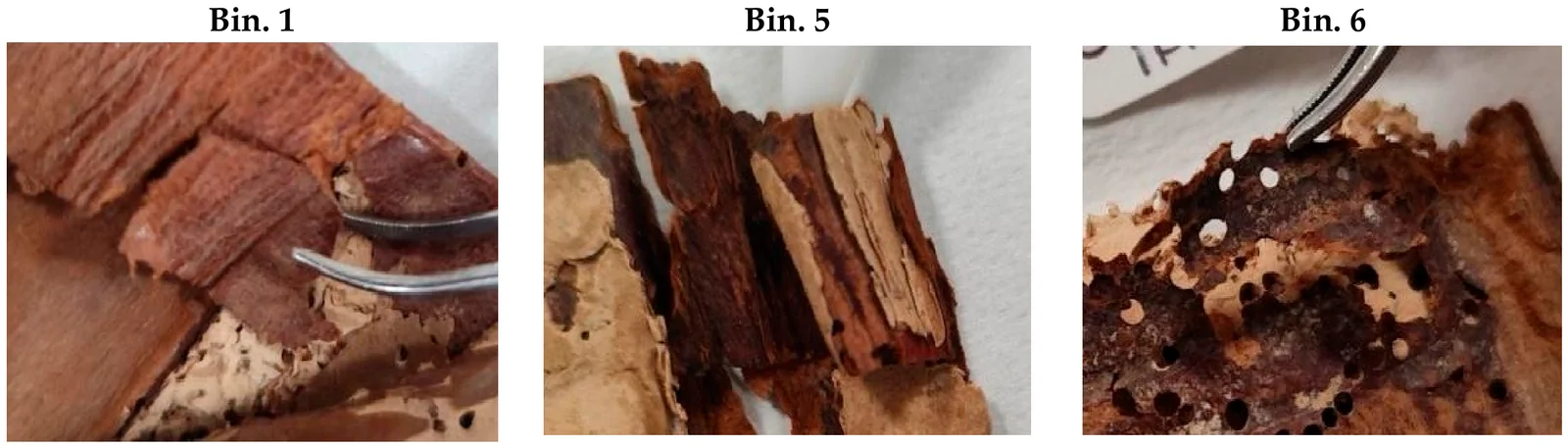
Original non-relocatable fragments collected from original samples.

The real leather samples were collected from three bindings belonging to the Library of the University of Naples “L’Orientale”, referred to as Bin. 1, Bin. 5, and Bin. 6. The bindings were selected according to their different conservation states, characterised by hardening, fragility, chromatic alterations, and loss of fibre cohesion. Sampling was carried out using non-relocatable fragments collected from marginal and non-structurally significant areas, to minimise the impact on the artefacts (Figure 1).

### 2.2. Laboratory Samples Preparation

Laboratory samples were assembled using vegetable-tanned goat skin in order to reproduce different degradation conditions and obtain representative samples reflecting the conservation state of the original artefacts. This was chosen as the most comparable material to the leather of the investigated bindings, based on microscopic and spectroscopic observations performed on the original artefacts. These samples provided a homogeneous reference material suitable for controlled ageing and preliminary assessment of the conservation treatments prior to their application on fragments of the original samples.

Measurements (e.g., color, spectroscopy, degradation tests) on laboratory samples were primarily performed on the grain side (hair side), as this is the surface that is historically and aesthetically most significant, and the one that sustains the greatest visual and functional impact from degradation. If the goal is to understand how the material degrades in its entirety over time, it is ideal to perform measurements on both sides (hair and flesh) to compare the resistance of the hair layer (grain) against that of the fibrous layer (flesh). This is relevant to the assessment of consolidation Efficiency (%), addressing Reviewer 2′s fourth point/observation, where we also measured Efficiency (%) on both the flesh and hair (grain) sides (as reported later in the manuscript and in the Supplementary Section).

#### Artificial Ageing Procedures

Two artificial ageing protocols were employed to obtain different degradation levels comparable to those observed on the original artefacts:

- Severe ageing by Inorganic Acids (IA): the samples were immersed in a 6 M hydrochloric acid (HCl) solution for 40 min, rinsed with water, and soaked in deionised water for 24 h. Tannic acid was then applied to the hair side at concentrations of 2 g/L and 10 g/L. After drying at room temperature, the samples were artificially aged according to UNI EN ISO 17228:2015 for 96 h at 50 °C and 90% RH [29,30].
- Standard ageing free from Inorganic Acids (INA): the samples were treated directly with tannic acid solutions (2 g/L and 10 g/L), without HCl pre-treatment, and then treated with the same accelerated ageing conditions (96 h, 50 °C, 90% RH).

**Scheme 1:**
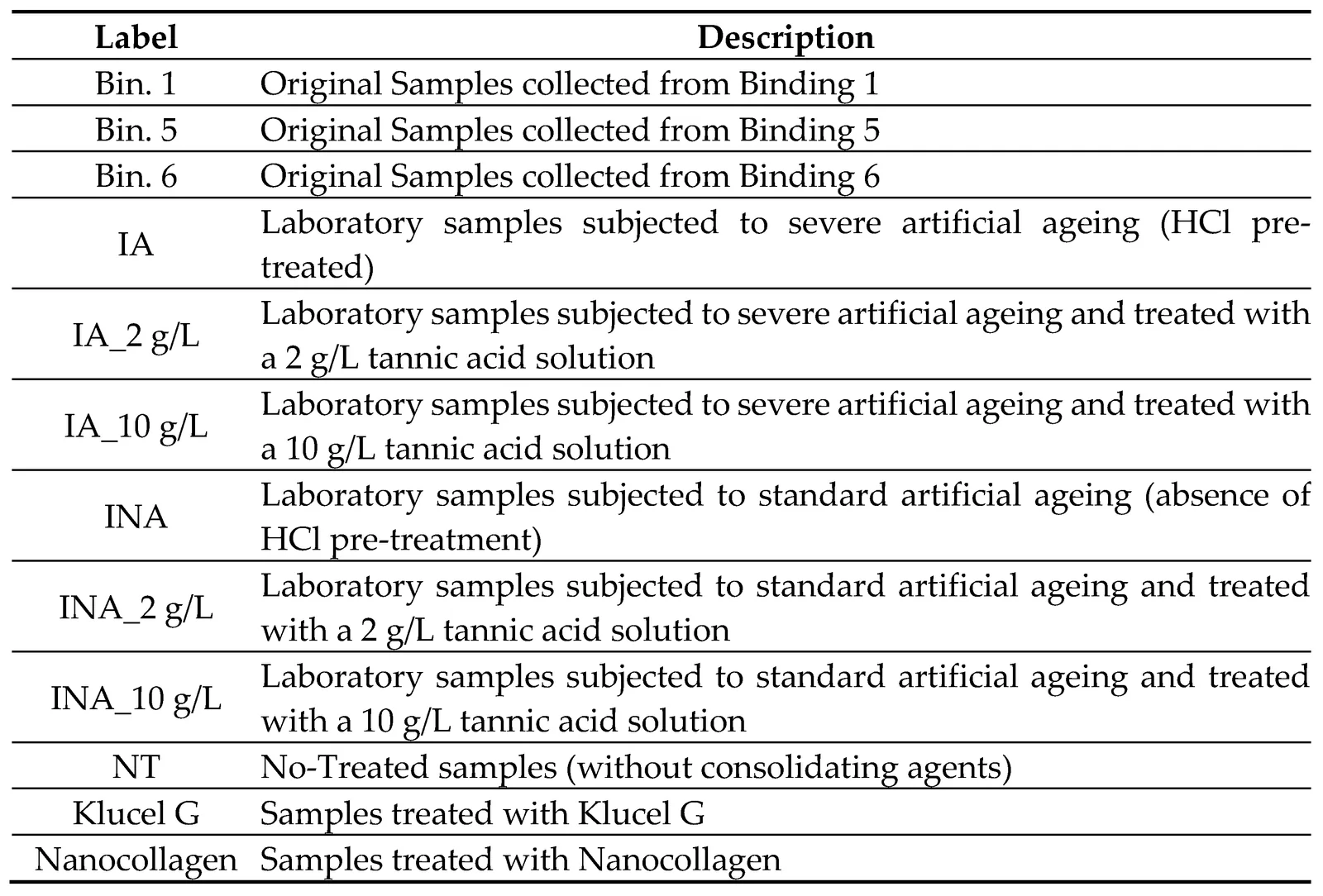
Representative panel of analysed samples.

In Scheme 1, a summary presentation including a detailed description of all analyzed samples has also been provided.

### 2.3. Consolidation Treatments

The consolidation treatment was designed to restore cohesion and structural stability to the leather bindings, without altering their physical–chemical and aesthetic properties. Two consolidants were employed for the experimental validation: Klucel G, used as a conventional consolidating agent, and Nanocollagen, selected for the highest compatibility with the proteinaceous matter. Klucel G (CTS Italia, Altavilla Vicentina, Italy) was dissolved in ethanol (Sigma-Aldrich, Darmstadt, Germany) at a concentration of 2% *w*/*v* [16,20,21,24]. Nanocollagen was electrochemically synthesised according to Bicchieri, Valentini et al. (2018) [14] and Patent N. 102016000096336 (UA2016A006832). Briefly, the tropocollagen precursor was used at a concentration of 1 mM in 0.1 M acetate buffer solution at pH 4.7. The alumina template membrane had the features reported in Patent N. 102016000096336 (UA2016A006832). The template membrane-assembled working electrodes are made conductive by means of an Ag layer, having 20 nm thickness (previously deposited by sputtering: current intensity of 2 mA, for 2 min). A constant working potential (−1.0 V/versus Ag/AgCl/Cl^−^ as the reference electrode) was applied by the chronoamperometry technique. The electrochemical deposition time was 3600 s, and it was performed under N_2_ flow (at a flow rate of 0.3 cm^3^/min). The synthesised Nanocollagen powder was very well dispersed in a 70:30% (*v*/*v*) hydroalcoholic mixture of 2-propanol (Sigma-Aldrich, Darmstadt, Germany) and distilled water, at the concentration of 0.7 mg/mL [14]. Both consolidants were applied by brush to laboratory samples and original fragments. The treatment consisted of three applications on both the hair and flesh sides, allowing complete solvent evaporation between consecutive applications. The samples were then left to dry for at least 24 h before measurements. A quantitative estimate of the efficiency of consolidation treatments performed using Nanocollagen is also reported; the experimental procedure for measuring treatment efficiency (both at the initial application and after two years) is described in detail below.

*Experimental evaluation of the efficiency over time to evaluate the effects of aging.* The proposed Equation for the evaluation of the Modified Global Efficiency Index (I_CE_) is reported below (Equation (1)):

(1) $$I_{\mathrm{CE}}=w_{1}\;\times \;E_{\mathrm{Cohesion}}\;+w_{2}\times \;E_{\mathrm{Elasticity}}+\;w_{3}\;\times \;P_{\mathrm{Aesthetics}/\mathrm{Color}}$$

where w_1_, w_2_, w_3_ are the weighting coefficients (which must sum to 1) that determine the relative importance of each parameter based on the initial state of degradation of the Art-Work objects. The definitions and measurements of the individual parameters are detailed below.

*Surface Cohesion Efficiency* (E_Cohesion_). Degraded historic leather (especially when affected by “*red rot*”) suffers from powdering caused by the breakdown of the collagen network. The goal of Nanocollagen treatment is to bind the powdery fibers and to calculate it, the principle of weight loss due to detachment is used (e.g., via a standardized Scotch Tape Test or contrast microscopy). The formula is given by Equation (2):

(2) $$E_{\mathrm{Cohesion}}(\%)\;=\left(\frac{M_{\mathrm{before}\;}-M_{\mathrm{after}}}{M_{\mathrm{before}}}\right)\times 100$$

where M is the mass of dust/fibers removed before and after the treatment. The closer the value is to 100%, the more effectively the consolidant has arrested the powdering.

*Elasticity Retention Efficiency* (E_Elasticity_). Unlike with Pietraforte, where the goal was an increase in strength, with leather the aim is to preserve the cover’s original flexibility, avoiding a “*cardboard-like*” effect or brittleness caused by stiffening. This parameter is calculated by monitoring Young’s modulus (E) or the breaking stress, though the relationship is inverse in cases of excessive stiffening. An effective formula for measuring the extent to which the leather has remained “close” to its ideal elasticity (or has regained plasticity without becoming stiff) is provided by Equation (3):

(3) $$E_{\mathrm{Elasticity}\;(\%)\;=\;}\left(1-\;\frac{\left|{\mathrm{Strain}}_{\mathrm{Target}\;}-\;{\mathrm{Strain}}_{\mathrm{Post}}\right|}{{\mathrm{Strain}}_{\mathrm{Target}}}\right)\;\times 100$$

If the treatment maintains the elongation capacity of the healthy or tolerated standard, efficiency is maximized.

*Aesthetic Penalty Factor* (PAesthetics/Color). The antique book covers from the University of Naples “L’Orientale” cannot tolerate whitening or changes in gloss induced by the consolidant (an effect typical of certain nanoparticles that are not perfectly dispersed). This factor acts as a negative value that reduces overall efficiency if the alteration is perceptible.

This additional parameter can be calculated via spectrophotometry to determine the ΔE. The formula is as follows (Equation (4)):

(4) $$P_{\mathrm{Aesthetics}/\mathrm{Color}}\;=\;\left(\frac{{\Delta E}^{*}\;-2}{{{\Delta E}^{*}}_{\max\;\mathrm{accetable}}}\right)\times 100$$

If ΔE* < 2, the aesthetic and color change is invisible to the human eye, and PAesthetic/Color = 0.

If ΔE* > 2, P_Aesthetic/Color_ indicates that the color shift exceeds the maximum acceptable limit for book restoration; for instance, the penalty reaches its maximum value, thereby destroying the overall efficiency index. Since the aim of this further study was to understand how the material degrades in its entirety over time, it is ideal to perform measurements on both sides (hair and flesh) to compare the resistance of the hair layer (grain) against that of the fibrous layer (flesh). This is relevant to the assessment of consolidation Efficiency (%) exhibited by Nanocollagen treatments over time.

The integrated platform for measuring mechanical properties, such as elasticity (Young’s modulus) and tear resistance, combined with analytical techniques that track parameters like cohesive forces, pH-potentiometric probes and optical or color characteristics (e.g., yellowing, chromatic alterations), constitutes an integrated sensor system. This last enables continuous, long-term monitoring of the condition of Cultural Heritage assets, particularly archival and library materials (indoor Cultural Heritage), which require constant surveillance due to frequent handling by end-users for both academic study and scientific research.

### 2.4. Analytical Methods

#### 2.4.1. Optical Microscopy

Optical microscopy observations were carried out on both original and laboratory samples in order to identify the animal species (from which the raw leather material is derived), assess the state of conservation of the leather, and evaluate the effectiveness of the consolidation treatments. The analyses focused on surface morphology, microcracks, abrasions, deposits, and morphological modifications induced by the treatments. Observations for animal species identification were performed using a Leica Microsystems Leica MZ75 microscope (Leica Microsystems GmbH, Wetzlar, Germany) with magnifications ranging from 2.5× to 5×, through the examination of the morphology and distribution of hair follicles. Further investigations were carried out using a DinoLite AM411-FVW digital optical microscope (AnMo Electronics Corporation, New Taipei City, Taiwan) at magnifications of 60× and 180× under visible (VIS) and ultraviolet (UV) radiation.

#### 2.4.2. pH Measurements

The pH measurement was carried out by water extraction (performed at R.T.), following the procedure described in ISO 4045:2018 (cold extraction protocol) [31]. The pH of the obtained solution was potentiometrically measured using a pH meter apparatus, Model 2335 Digital pH meter (from Amel Electrochemistry Srl, Milan, Italy), equipped with a 411/CGG/6 combined pH electrode, having Ag/AgCl as the reference electrode, glass body, Ø4.5 mm × 100 mm, S7 connector, 3.3 M KCl. The pH difference was evaluated by diluting the solution used for pH measurements 10 times. The measurements were carried out following the cold extraction standard test ISO 4045:2018 (*ISO 4045:2018 (IULTCS/IUC 11)-Leather-Chemical tests-Determination of pH*).

#### 2.4.3. Qualitative Chemical Assays

To investigate the chemical composition of the raw materials, chemical assays were performed to verify the presence of organic and inorganic compounds associated with tanning and manufacturing processes. In particular, ferric chloride was used to detect tannins, rhodamine for hydrolysable tannins, the butanol-HCl test for condensed tannins, and alizarin for the identification of potash alum associated with mixed tanning processes [32,33].

#### 2.4.4. FTIR (Fourier Transform Infrared Spectroscopy) Spectroscopy

Measurements were performed on both the hair side and the flesh side of the samples (32 scans per sample, in the 400–4000 cm^−1^ spectral range with a resolution of 4 cm^−1^). The analyses were carried out directly on the sample surfaces, without further preparation, using a Thermo Scientific Nicolet Summit PRO spectrometer (Thermo Fisher Scientific Inc., Waltham, MA, USA) equipped with an ATR Everest Diamond accessory. Far-IR (Far Infrared) investigations were additionally performed on the real samples using a VERTEX 70 FTIR spectrophotometer (Bruker Corp., Billerica, MA, USA).

#### 2.4.5. Raman Spectroscopy

Measurements were acquired directly on the untreated sample surfaces, using a portable RK785-I spectrometer (Oceanhood Taiwan Opto-Electronics Co., Ltd., Taoyuan City, Taiwan), equipped with a 785 nm laser source. The acquisition conditions included a laser power ranging from 50 to 200 mW, an integration time of 3000 ms, a spectral range of 200–3000 cm^−1^, and a spectral resolution of 4 cm^−1^.

#### 2.4.6. Scanning Electron Microscopy (SEM) Coupled with Energy-Dispersive X-Ray Analysis (EDAX) Characterization

The observations were performed using a variable-pressure scanning electron microscope (VP-SEM), model Hitachi SU3500 (Hitachi High-Tech Corporation, Tokyo, Japan), operated under low-vacuum conditions (30 Pa) with an accelerating voltage of 8 kV. The system was equipped with dual energy-dispersive X-ray detectors (dEDS, Bruker XFlash^®^ 6|60; Bruker Nano GmbH, Berlin, Germany) for microanalysis/elemental analysis. The samples were assembled on aluminium stubs using carbon tape and analysed without further preparation, working at magnifications of 500× and 1000×.

#### 2.4.7. Mechanical Tests

The tests were performed using an MTS Insight 5 instrument (model 569329-02, MTS Systems Corporation, Eden Prairie, MN, USA) on samples with a width of 10 mm, a gauge length of 50 mm, and a thickness between 1.2 and 1.5 mm. Due to dimensional limitations of original samples, the analyses were carried out exclusively on laboratory samples. For each treatment, the reported value corresponds to the average of three independent measurements.

#### 2.4.8. Spectrocolorimetry

Spectrocolorimetric analyses were carried out to evaluate chromatic variations induced by the consolidation treatments and to assess their aesthetic compatibility with the original material. Measurements were performed on both laboratory samples and non-relocatable fragments using a 3 nh YS3060 spectrocolorimeter (Shenzhen ThreeNH Technology Co., Ltd., Shenzhen, China). For each point, the average of five consecutive measurements was recorded to ensure data repeatability. The results were processed using the ΔE* parameter, calculated according to the CIELab (1976) method. In agreement with the reference literature, ΔE* values lower than 3 were visually imperceptible, values between 3 and 5 were considered acceptable for conservation applications, and values higher than 5 indicated evident chromatic variation unsuitable for historical and artistic surfaces [34,35].

## 3. Results and Discussion

This chapter is organized as follows: the results are presented starting with the characterization of the raw materials making up the samples (to identify the status of conservation 3.1.), moving on to the laboratory-reproduced samples (for validation of conservation treatments 3.2.), and finally to the original ones (for conservation of the original samples, 3.3.). Following the presentation of experimental data, a brief yet significant discussion is provided (at the end of the results for each sample type) regarding key information essential to understanding the conservation process based on Nanocollagen and compared with the Klucel G traditional treatment.

### 3.1. Characterisation of Raw Materials: Status of Conservation

**Figure 2:**
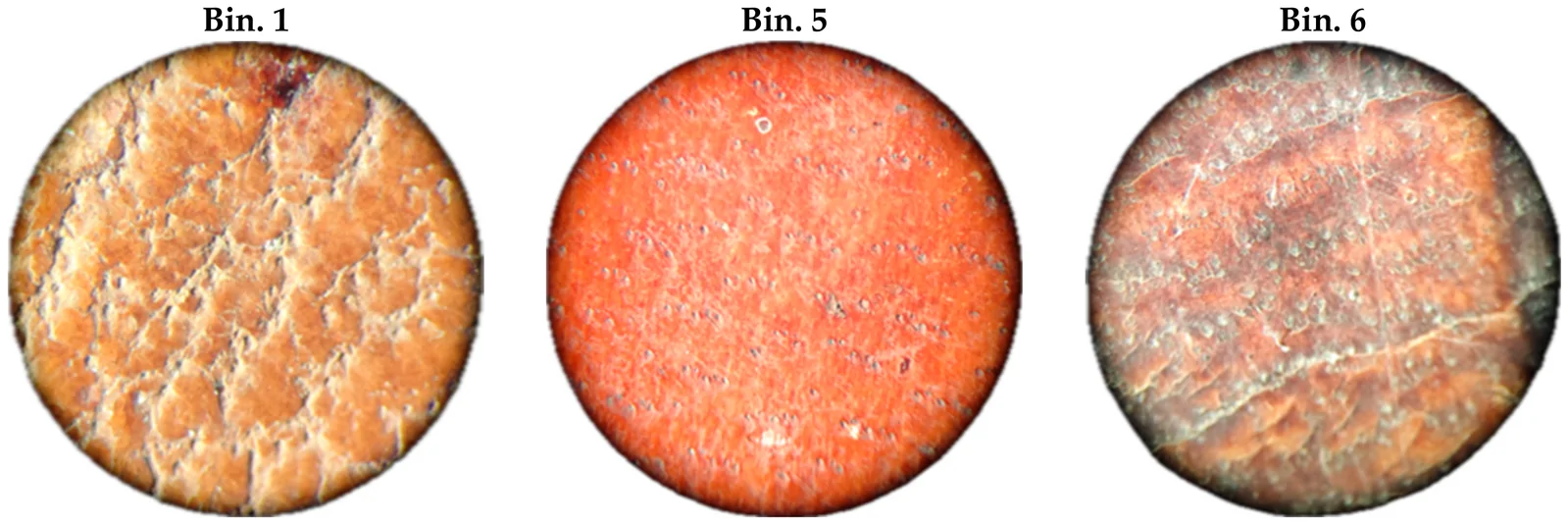
Optical microscopy for the identification of the animal species of the leather used for the original bindings. Bin. 1: Original Samples collected from Binding 1; Bin. 5: Original Samples collected from Binding 5; Bin. 6: Original Samples collected from Binding 6.

Optical microscopy analysis allowed the identification of the animal species (from which the skin was extracted) in Bins. 1 and 6 (Figure 2). The morphology and distribution of the hair follicles, arranged in parallel rows and characterised by follicular groups composed of three units consisting of one primary pore associated with smaller secondary pores, are typical of goatskin [20,36,37]. In contrast, for Bin. 5, the identification of the animal species was more complex, as the arrangement of the hair follicles was not clearly visible.

Optical microscopy under VIS and UV illumination provided complementary information on the surface condition of the historical bindings (Table 1). VIS observations allowed the morphological features of the leather to be assessed, including the preservation of the grain layer, exposure and cohesion of the fibrous structure, cracks, surface deposits, and the presence of adhering materials. UV illumination provided complementary information based on differences in fluorescence response, allowing areas of surface heterogeneity and materials showing a distinct response from the surrounding leather to be highlighted and spatially correlated with the corresponding VIS observations.

Bin. 1 showed widespread deterioration of the leather surface, with partial loss of the grain layer and exposure of the underlying fibrous structure, particularly along the margins. Several lighter subcircular areas were also visible under VIS illumination; the same areas were recognizable under UV illumination, where they exhibited weak fluorescence.

Bin. 5 showed marked deterioration of the surface layer, with loss of the grain layer and extensive exposure of the collagen fibrous structure. The fibres appeared disaggregated and poorly cohesive, particularly in the marginal areas. Paper residues were also observed on the flesh side, and the fluorescence detected under UV illumination was associated with these residues.

Bin. 6 exhibited a different degradation pattern, characterized by linear fractures and microcracks, surface deposits, and numerous holes attributable to entomological activity. On the flesh side, VIS observations also revealed material adhering to the leather surface. Under UV illumination, this material exhibited intense yellowish fluorescence, spatially coincident with the adhering material observed in the corresponding VIS images.

The analysed leather samples (Bin. 1, 5, 6) showed pH values close to neutrality and ΔpH values ranging from 0.11 to 0.20, indicating negligible acidity of the samples. (Table 2). If the differential index (ΔpH) measured on samples of damaged historic leather (such as those from the library of the “Orientale” University in Naples) shows low values below the threshold of concern, this indicates the absence of free strong mineral acids and suggests that the moderate acidity detected is attributable to stable weak organic acids (e.g., tannins or fatty acids). A ΔpH greater than 0.7 (or 1.0, depending on specific guideline limits, UNI EN ISO 4045) is a major warning sign, as it indicates the presence of a free strong acid (such as sulfuric acid resulting from atmospheric deterioration or old tanning treatments) that causes acid hydrolysis and the irreversible destruction of collagen (“red rot” effect). Consequently, a markedly low ΔpH value (<0.7) confirms that, although the historic leather may appear structurally damaged by other factors (e.g., oxidation, heat, mechanical trauma, or fluctuations in temperature and humidity), there is no active degradation accelerated by strong mineral acids.

Concerning the composition of the leather, qualitative chemical tests revealed similar results for all the bindings, characterised by the presence of predominantly condensed tannins and potash alum (KAl(SO_4_)_2_·12H_2_O), while hydrolysable tannins were not identified (Table 2). Concerning the composition of the leather, qualitative chemical tests showed comparable results for the three historical bindings (Table 2). All samples gave a positive response to the ferric chloride test, with the development of a violet coloration indicating the presence of tannins. The butanol–HCl test also gave a positive response in all three samples, characterized by the development of a reddish coloration and supporting the presence of condensed tannins. Conversely, the rhodamine test, used to assess the presence of hydrolysable tannins, did not produce the expected reddish coloration in any of the samples. This negative response may indicate either the absence of hydrolysable tannins or their presence at concentrations below the detection limit of the qualitative test. Finally, the Alizarin S test produced a dark coloration in Bin. 1, Bin. 5 and Bin. 6, indicating the presence of aluminum salts. Overall, the qualitative tests revealed a comparable composition among the three bindings, characterized by the presence of tannins, predominantly of the condensed type, and aluminum salts attributable to alum.

**Figure 3:**
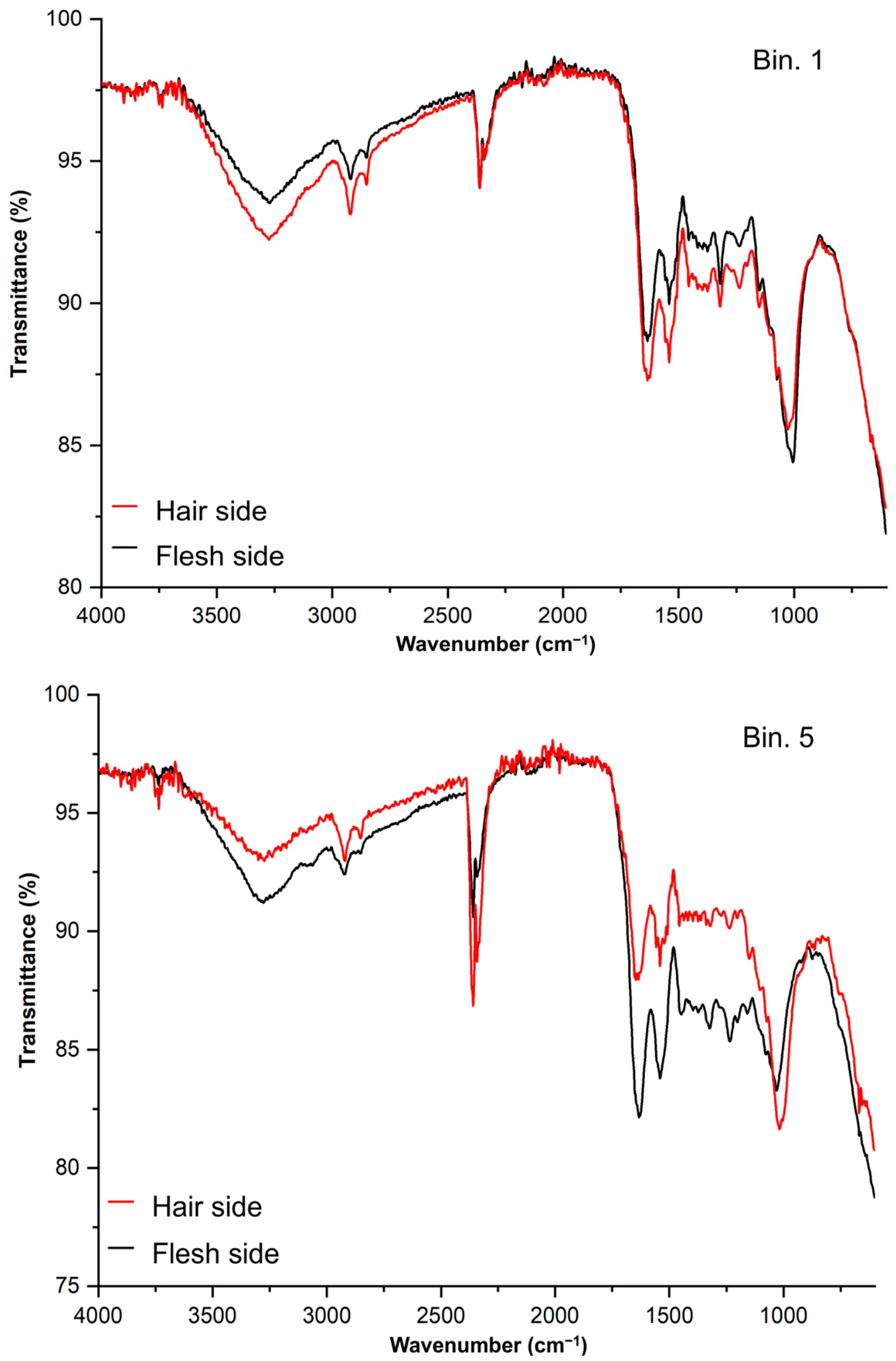

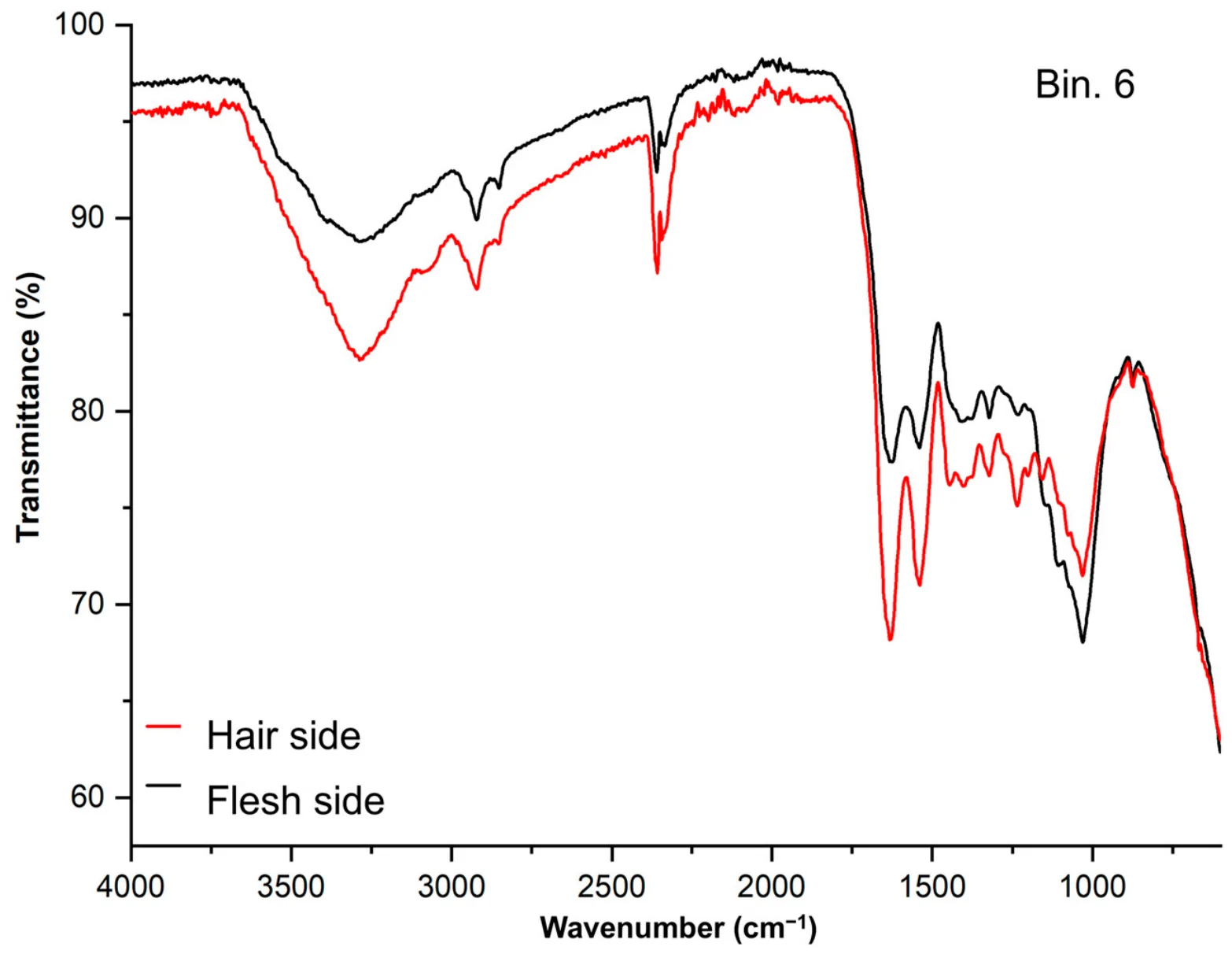
FTIR spectra of original samples, collected on the hair side (red) and flesh side (black). Bin. 1: Original Samples collected from Binding 1; Bin. 5: Original Samples collected from Binding 5; Bin. 6: Original Samples collected from Binding 6.

These results are consistent with the FTIR analyses, which revealed limited compositional differences among the samples, mainly related to the relative intensity of the spectral signals rather than a significant frequency shift (Figure 3).

All samples exhibited a proteinaceous component attributable to collagen, identifiable through the main amide bands (Amide B, I, II and III), located at ~3070, 1650, 1630, 1540 and 1235 cm^−1^ respectively [38,39]. Regarding the organic fraction, bands attributable to tannin compounds were identified, mainly recorded in the range of 1330 and 776 cm^−1^ [38,39,40,41,42]. The presence of alum is confirmed by the sulphate bands in the mid-IR region (~1235, 1105 and 670 cm^−1^) and by the Al–O vibrations, detected at ~876, 680 and 642 cm^−1^ [42,43,44,45]. Contributions related to hydrated aluminium complexes were also evident in the Far-IR region (Figure S1 in the Supplementary Section). Bands associated with silicates/aluminosilicates were also detected within the 1050–1000 cm^−1^ range [45,46,47]. The presence of carbonates was suggested by the band observed between 230 and 220 cm^−1^, exclusively detected in Bins 1 and 6 [6,48]. Finally, the presence of starch was also identified in Bins 1 and 5 through the bands at ~924, 865 and 775–757 cm^−1^ [49]. A detailed band assignment is highlighted in Tables S1–S4 (Supplementary Materials) [50,51].

**Figure 4:**
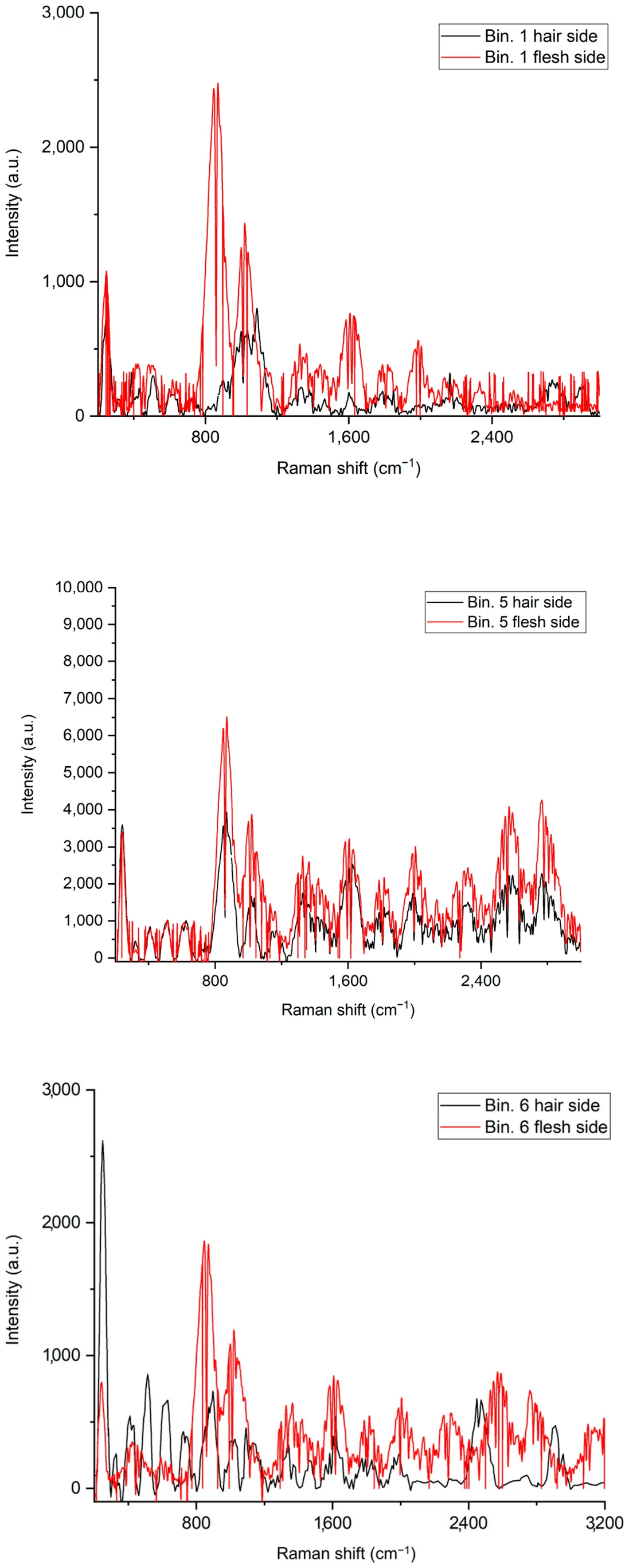
Raman spectra of all original samples, collected on the hair/grain side (black) and flesh side (red). The omission of the Raman profile for the flesh side of the original leather samples in the previous version of the manuscript is justified by the higher background noise present in the corresponding spectrum (and this is due to the differing chemical composition of the flesh side of the antique leather compared to its hair/grain side). Furthermore, the flesh side is also the one least exposed to environmental damage and wear and tear from end-users. Bin. 1: Original Samples collected from Binding 1; Bin. 5: Original Samples collected from Binding 5; Bin. 6: Original Samples collected from Binding 6.

Raman spectroscopy confirmed the presence of a complex matrix containing both organic and inorganic compounds, present in all the samples, despite the high background noise caused by the fluorescence of the organic fraction, particularly evident in Bin. 1 and Bin. 5 and, more generally, on the flesh side of all the analysed samples (Figure 4). The reason for this marked difference and the nature of the phenomenon depends on specific chemical and structural factors inherent to the two sides of the processed leather. In particular, the hair side (also known as the grain side) exhibits a higher concentration of tannins and tanning agents, which are responsible for the fluorescence signals, as well as the presence of organic residues and degradation products (aging processes that also contribute to the fluorescence signal) and, finally, specific surface morphology. Indeed, the hair side retains the characteristic structure of hair follicles and the skin’s grain pattern. This microscopic roughness and structural heterogeneity can increase diffuse scattering and optical dispersion, thereby “blurring” or distorting the signal compared to a more uniform surface. The organic compounds exhibit bands attributable to collagen and tannins, associated with the main structural amino acids of collagen and with aromatic, C–O and O–H vibrations of tannin compounds, respectively [49,52,53,54,55]. To overcome this problem and extract significant Raman peaks from the fluorescent background, further investigations will employ near-infrared laser excitation sources (e.g., at 1064 nm using the FT-Raman hyphenated technique) or advanced methods such as time-gated Raman spectroscopy.

The inorganic component instead reveals signals attributable to sulphates, carbonates, silicates/feldspars and alumina. Sulphates can be identified through the bending modes centred at ~427 and 626 cm^−1^, as well as the symmetric and asymmetric stretching vibrations recorded at ~973–1001 and ~1092 cm^−1^, together with bands associated with hydrogen-bond interactions acquired at higher frequencies (1833, 1984, 2466 cm^−1^) [56,57]. Carbonates are recognisable through the characteristic ν_4_ mode at ~722 cm^−1^ and the ν_1_ C–O stretching around 1090 cm^−1^, whereas silicates and feldspars are identifiable through the Si–O–Si/Si–O–Al vibration located at ~511 cm^−1^ [52,58,59]. A detailed assignment of the Raman bands is also reported in Table S5 and Figure S2 (Supporting Material). The results obtained through FTIR spectroscopy (combined with Raman data) also enabled an assessment of the conservation state of the leather binding samples. In particular, Table 3 reports the main spectral parameters used to evaluate the degree of degradation of the original samples. The analysed samples did not show significant evidence of oxidation of the collagen matrix. The IAI/IAIII ratio obtained with Raman measurements revealed, in the case of the Bin. 1 sample, typical damage due to the structural order-disorder protein transitions [10]. FTIR parameters associated with hydrolytic degradation exhibited values commonly reported in the literature as indicative of hydrolytic alterations of collagen [17], as shown here by the original Bin. 5 sample. To explain the enhanced resistance and peculiar preservation of Bin. 6 (which appears to be affected only by biodeterioration events (and not by physicochemical damage) compared to Bin. 1 and Bin. 5, two main non-mutually exclusive hypotheses can be advanced:

- Supplemental treatment or protective finishing. It is highly probable that Bin. 6 underwent a supplemental surface finishing treatment (e.g., application of waxes, hydrophobic oils, or specific tanning/retanning agents) during or after manufacturing. This chemical-physical barrier may have shielded the collagen matrix from moisture absorption and acidic atmospheric pollutants, thereby preventing both the acid hydrolysis observed in Bin 5 and the thermal/structural destabilization (order-disorder transition) seen in Bin 1. Conversely, the presence of organic fatty or proteinaceous finishing substances could have provided an ideal nutrient substrate for microorganisms, justifying the prevalence of biological degradation alone.
- Intrinsic nature and leather quality. Alternatively, the discrepancy could lie in the very nature of the original material. Differences in animal species (e.g., calfskin vs. sheep/goatskin), the animal’s age, or the specific tanning recipe (e.g., a higher density of stable vegetable tannins) might have granted Bin 6 superior intrinsic hydrolytic stability. A naturally denser and well-crosslinked collagen network resists chain scission and chemical denaturation more effectively, succumbing only to the enzymatic action of biodeteriogens over the long term.

In conclusion, the thermal and chemical vulnerability of Bin. 1 and Bin. 5 indicates a loss of structural integrity of the leather, whereas Bin. 6 maintained its molecular architecture intact against physicochemical factors, yielding only partially to biological attack.

Ultimately, therefore, the only detailed scientific information regarding these original samples is precisely that derived from this scientific study, given the current lack of authoritative historical sources documenting the conservation history and dating of the specimens originating from the University of Naples “L’Orientale.”

**Figure 5:**
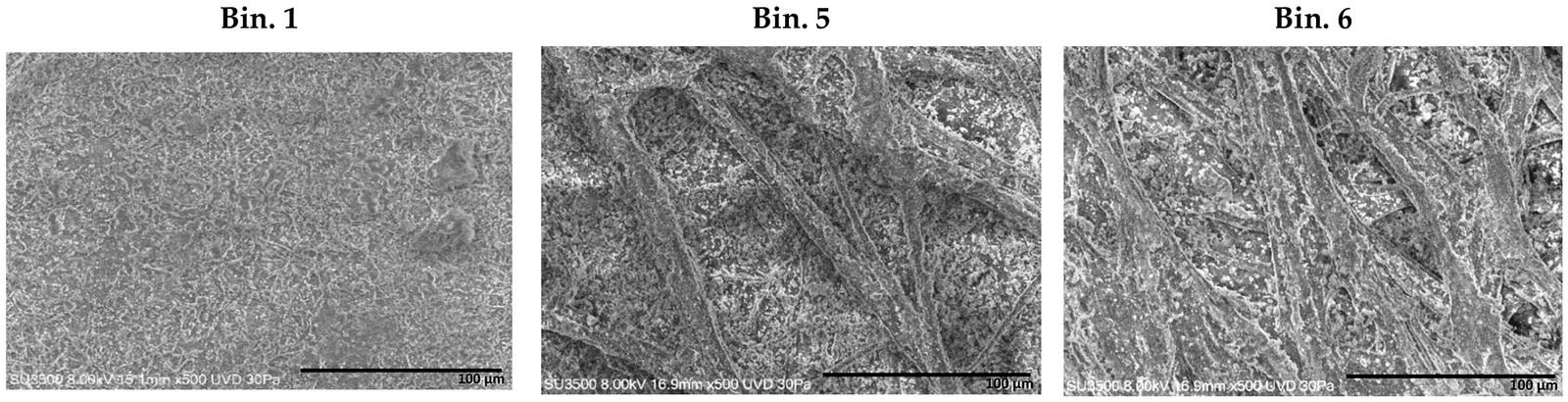
SEM micrographs of the original samples for Bin. 1 Bin. 5 and Bin. 6. Magnification of 500×. In the first phase (without nano-collagen), the SEM imaged the “bare” surface morphology or dry contaminants, provided the samples had been properly metal-coated (e.g., gold or carbon sputtering) or analyzed in low-vacuum mode (Variable Pressure, as in our case study). The SEM measurement will differ, however, following treatment of the original samples with Nanocollagen. Bin. 1: Original Samples collected from Binding 1; Bin. 5: Original Samples collected from Binding 5; Bin. 6: Original Samples collected from Binding 6.

SEM-EDS investigations provided further insight into the different conservation states of the bindings. In particular, the micrograph of Bin. 1 showed significant fragmentation of the collagen fibres, with dimensions smaller than 100 μm. In contrast, Bin. 5 and 6 exhibited fibrillar bundles with more rounded morphologies and more preserved fibrillar structures (Figure 5). EDX microanalysis revealed the presence of a mineral component mainly characterised by calcium (Ca), potassium (K), magnesium (Mg), sodium (Na), aluminium (Al), silicon (Si), chlorine (Cl), and oxygen (O), as reported in Figure S3.

The useful information derived from the results obtained for the original samples concerns the composition (raw materials), the state of preservation and the treatments they underwent. Firstly, microscopic analyses revealed that Bins. 1 and 6 are composed of goat leather, allowing the identification of the animal species used for the manufacture of the artefacts. Indeed, the arrangement of the hair follicles is consistent with that reported in the literature for this animal species, which is characterized by follicular groups organized in parallel rows [20,36,37]. In the case of Bin. 5, however, the identification of the hair bulbs was not possible due to the degraded condition of the surface and/or the probable mechanical treatments undergone by the leather. In particular, the surface morphology may have been altered by finishing, pressing, oiling and polishing processes, traditionally employed to reduce surface irregularities and increase the uniformity of the membranous support [62,63]. Microscopic observations also highlighted different degradation levels among the analysed bindings:

- Bin. 1 exhibited the most pronounced morphological evidence of degradation among the analysed bindings. Optical microscopy observations and SEM investigations revealed exposed and highly fragmented structural fibres associated with leather powdering phenomena and loss of cohesion among collagen fibrils [20]. These features indicate a marked deterioration of the collagen network, characterised by the progressive loss of the original fibrillar organisation and structural integrity of the leather support. UV observations also revealed weak fluorescence corresponding to the lighter subcircular areas observed under VIS illumination. This response may be related to the chemical nature of the main organic components of leather. FTIR analysis confirmed the presence of vegetable tannins in the leather samples through their characteristic bands, mainly observed in the 1330–776 cm^−1^ region. The extended conjugated aromatic structures of vegetable tannins are responsible for their intrinsic fluorescence under UV excitation, which may vary according to their chemical composition and state of ageing [64]. A further possible contribution to the fluorescence response may arise from the collagen matrix, the main protein component of leather. During ageing, oxidative processes affecting aromatic amino acid residues, particularly tyrosine, can lead to the formation of fluorescent oxidation products, such as dityrosine, which may contribute to the UV-induced fluorescence of aged proteinaceous materials [65]. These processes may be accompanied by structural changes in collagen, detectable through modifications of its characteristic spectroscopic features [65]. In Bin. 1, FTIR and Raman analyses confirmed a significant alteration of the collagen matrix: FTIR parameters indicated hydrolytic degradation, while Raman data revealed an order–disorder transition of the protein component. However, the absence of the FTIR band in the 1720–1740 cm^−1^ region, used in the present study as an indicator of oxidative phenomena, provided no significant spectroscopic evidence of collagen oxidation. Therefore, although the collagen matrix and its ageing products may potentially contribute to the fluorescence of historical leather, the fluorescence observed in Bin. 1 cannot be specifically associated with oxidative degradation of collagen. Overall, the degradation appears to be mainly associated with the progressive loss of cohesion and structural integrity of the collagen matrix, rather than with advanced protein denaturation processes. However, the Raman data in Table 3 indicate damage typical of an order-disorder protein transition, attributable to the initial stage of the phenomenon known as “red rot,” common in antique skins and leathers. The “red rot” effect in antique leathers and skins is closely linked to a highly acidic pH level. This chemical degradation, typical of vegetable-tanned leathers, transforms the leather into a brittle, reddish powder [66]. In the case of study Bin. 1, the measured pH does not indicate strong acidity in the sample; therefore, the effect may be present, but at an early rather than an advanced stage.
- Bin. 5 exhibited leather powdering phenomena, with exposure of structural fibres detectable by optical microscopy, similarly to what was observed in Bin. 1. SEM investigations, however, revealed less fragmented collagen fibrils, still capable of maintaining their three-dimensional structure. These features suggest that, although degradation caused a partial loss of cohesion of the material, the collagen fibrillar network remains relatively preserved. Paper residues adhering to the flesh side were also observed, and the fluorescence detected under UV illumination was associated with these residues. Spectroscopic analyses and pH measurements provided results comparable to those obtained for Bin. 1. FTIR parameters indicated hydrolytic alteration of the collagen matrix, without significant evidence of oxidative degradation, while Raman data showed Amide I/Amide III ratios below the threshold associated with an order-disorder transition, indicating a lower degree of structural alteration compared with Bin. 1. Overall, Bin. 5 exhibits an intermediate degradation state, characterised by surface alterations and a moderate loss of cohesion, while still preserving a substantial degree of organisation within the collagen fibrillar structure.
- Bin. 6 showed the least advanced degradation among the analysed samples. Indeed, the binding does not exhibit evident leather powdering phenomena and largely preserves the characteristic surface morphology of leather. However, cracks in the dermis attributable to the natural ageing of the support and insect-related holes associated with entomological degradation are present [20,67]. A distinctive feature of Bin. 6 is the material adhering to the flesh side, clearly visible under VIS illumination and characterised by intense yellowish fluorescence under UV illumination. As discussed for Bin. 1, fluorescence may also arise from intrinsic components of leather, including tannins and the collagen matrix and its ageing products. In this respect, FTIR analysis confirmed the presence of the tannin component and indicated only slight hydrolytic alteration of collagen, without evidence of oxidation or denaturation, while Raman data did not indicate an order–disorder transition of the protein structure. Therefore, although a contribution from the intrinsic components of leather cannot be excluded, the fluorescence in Bin. 6 is spatially coincident with the adhering material observed under VIS illumination, while the surrounding leather does not show a comparable response. Considering both the spectroscopic results and the spatial correlation between the VIS and UV observations, the localized fluorescence may be attributed to the presence of an adhesive residue, as several adhesives commonly used in historical objects are known to exhibit fluorescence under UV illumination [68,69]. Paper fragments were also found within the binding. Their presence, together with that of organic materials potentially associated with the binding structure, may have contributed to the observed entomological degradation, as paper and organic adhesives can provide suitable nutritional substrates for insects commonly found in historical book collections [70,71,72]. This interpretation is consistent with the localized distribution of the holes and with the overall conservation state of the binding. The better conservation state of the leather is primarily supported by the microscopic and SEM observations, which indicate a largely preserved collagen matrix, limited fragmentation of collagen fibres and the maintenance of the characteristic fibrillar organisation of the support. These findings are consistent with the spectroscopic analyses and pH measurements, which support the overall preservation of the collagen matrix and the limited extent of chemical degradation phenomena affecting the support. In summary, the observed degradation appears to be mainly associated with physical ageing and entomological attack, while the collagen matrix retains a substantial degree of structural integrity.

Regarding the characterisation of the constituent materials, the results showed that all bindings present substantial compositional homogeneity. The simultaneous presence of collagen, tannins and alum indicates that all bindings underwent a mixed tanning process, historically employed to improve the mechanical stability and aesthetic properties of leather [73]. The presence of carbonates may be associated with residues deriving from tanning processes, particularly lime-based treatments used during leather depilation [6]. Silicates and aluminosilicates are instead likely attributable to surface deposits and environmental contamination [13]. Finally, starch traces detected in Bins. 1 and 5 appear attributable to residues of adhesives used for paper mounting (starch glue), a practice frequently documented in Eastern book production [74]. The information collected through the multi-analytical characterization of the original samples guided the preparation of laboratory specimens (in the next Section 3.2), which were subsequently subjected to different artificial ageing protocols in order to simulate degradation conditions like those observed in the historical bindings.

### 3.2. Laboratory Samples: Validation Study

FTIR analyses have been mainly carried out on the grain/hair side of laboratory samples, consistent with what was previously explained in the experimental section.

**Figure 6:**
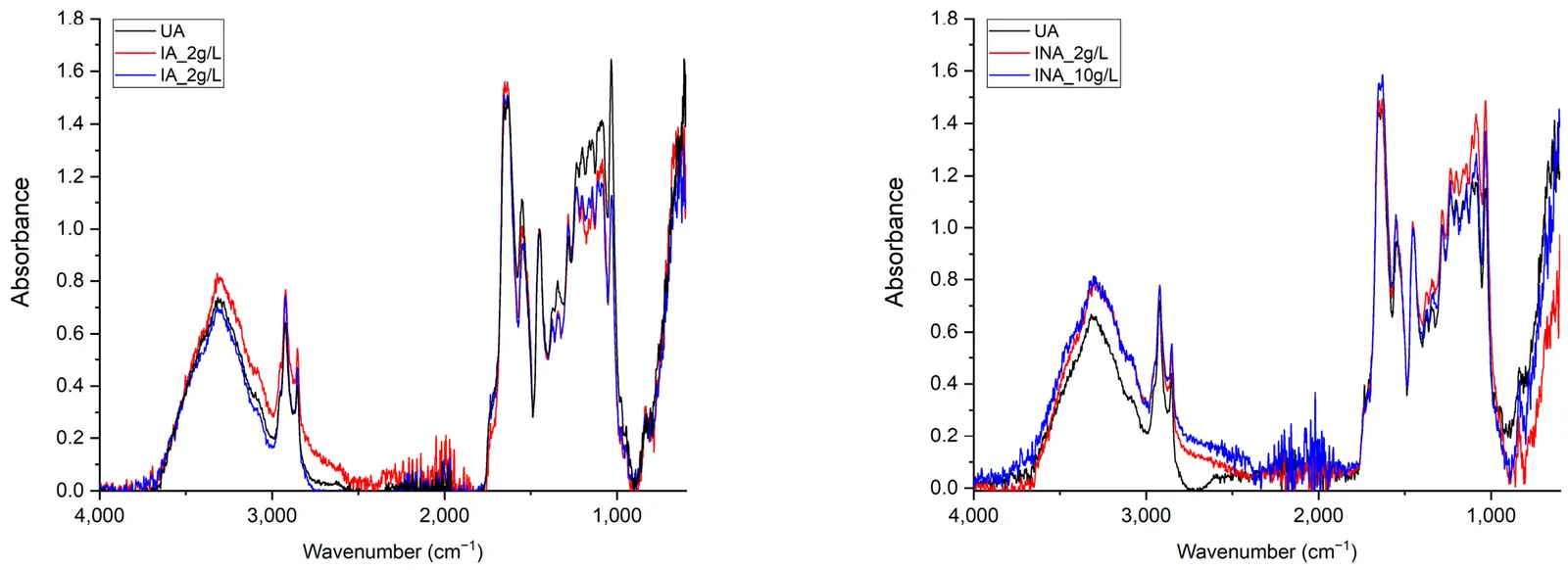
FTIR spectra of laboratory samples (hair/grain side) subjected to severe ageing protocol (IA, **left**) and standard ageing protocol (INA, **right**). For each protocol, the spectra acquired from the unaged sample (black), the sample treated with 2 g/L tannic acid (red), and the sample treated with 10 g/L tannic acid (blue) are reported.

Figure 6 of the unaged sample revealed the main bands attributable to collagen and vegetable tanning agents, confirming the composition of the support. The analyses also highlighted the different degradation conditions induced in the laboratory samples. In particular, the samples treated with HCl and tannic acid (severe ageing; IA_2 g/L and IA_10 g/L) showed an increase in the AI band and a decrease in the AII band, accompanied by a shift towards lower frequencies. These variations led to an increase in the AI/AII ratio; similarly, the Δν(AI–AII) parameter increased, reaching values higher than 95, indicative of advanced alteration processes affecting the collagen matrix [32,33].

Regarding the samples subjected to the standard ageing protocol (INA_2 g/L and INA_10 g/L), more limited variations were observed. An increase in both the AI and AII bands was detected, without the evident shift in the AII band towards lower frequencies observed in the severely aged samples. Moreover, Δν(AI–AII) values remained below 95, suggesting a more limited alteration of the collagen structure, which still appeared to be partially preserved [32,33].

After obtaining this useful information, treatments with Nanocollagen and Klucel G (used here for comparison) were also performed on laboratory samples; following treatment, these were characterized using optical microscopy, mechanical testing, and colorimetry (color analysis). Microscopic observations (Table 4) revealed significant differences between the two treatments: Nanocollagen did not alter the microstructure of the leather and allowed the hair follicles to be clearly recognised under both VIS and UV illumination. The effectiveness of Nanocollagen and the observed microscopic clarity can be explained by three main factors:

- Chemical-structural affinity (biomimicry). The hide (parchment or leather) is composed almost entirely of collagen. Nanocollagen shares the same chemical nature as the substrate. This allows it to integrate seamlessly into gaps within the dermal matrix without creating optical or structural discontinuities (thereby preserving the original microstructure).
- Nanoscale size and penetration. Unlike traditional polymers, nanoparticles penetrate deep into the microscopic fibers rather than remaining on the surface. They consolidate the structure from within, leaving morphological voids—such as hair follicles—open and clean.
- Response to light (VIS/UV) and absence of “filming”. As it does not form an opaque film and has a refractive index similar to that of degraded collagen, visible light passes through the material without anomalous reflections. Furthermore, collagen exhibits natural UV fluorescence (due to amino acids such as tyrosine and phenylalanine). Nanocollagen does not mask this fluorescence nor introduce spurious fluorescence, allowing morphological details (such as follicles) to stand out clearly.

The behaviour of Nanocollagen differs from that of Klucel G for several structural and optical reasons, as reported in Table S6 in the Supplementary Materials. In summary, while Klucel G acts primarily through surface mechanical adhesion, creating a film that risks flattening the specimen’s morphology and altering its optical response, Nanocollagen works through structural integration. The latter consolidates the fibrous network while leaving the surface “open” and chemically unaltered; consequently, hair follicles remain perfectly identifiable under the microscope regardless of the lighting conditions.

Klucel G, on the other hand, allowed the recognition of the hair follicles only under visible light, while exhibiting strong fluorescence under UV illumination. When examining damaged antique leather treated with Klucel G (hydroxypropyl cellulose) under an optical microscope using ultraviolet (UV) light, the observed fluorescence phenomenon is not generated by the consolidant itself, but rather by the optical-chemical interaction between the historical object’s degraded components and the protective agent’s carrier solvent [https://restaurotecnica.it/prodotto/klucel-g (accessed on 13 September 2026)]. The observed UV fluorescence is primarily due to three concurrent factors:

- Intrinsic fluorescence of degraded tanning materials. Antique leathers (often vegetable-tanned with condensed tannins) undergo thermo-hygrometric and oxidative processes over time, such as the well-known phenomenon of “red rot” caused by the absorption of acidic pollutants. Aging alters the structure of the collagen and tannins, generating by-products and oxidized aromatic compounds that exhibit strong intrinsic fluorescence when excited by UV radiation.
- Solubilization and migration effect of degradation products. Klucel G is typically applied as a solution in polar organic solvents, most often ethanol or isopropanol. The solvent penetrates the porous, structurally compromised matrix of the aged leather, partially solubilizing surface salts, altered original tanning fats, or hydrolysed tannins. As the solvent evaporates, capillary action draws these fluorescent compounds toward the surface, concentrating them within the newly formed microscopic polymer film.
- Modification of the film’s optical refraction properties. Pure hydroxypropyl cellulose (Klucel G) exhibits negligible or no intrinsic fluorescence when fresh. However, by forming a continuous film that fills the microscopic voids of the powdery leather, it acts as an “optical trapping lens.” By altering the surface refractive index, it renders underlying fluorescent compounds much more visible and sharply defined under the microscope compared to untreated leather, which scatters light due to its high surface roughness.

Regarding the mechanical investigations, differences were observed depending on both the degradation level induced in the laboratory samples and the consolidating agents used for the treatments (Table 5). In untreated samples, stress and strain values varied according to the ageing protocol adopted; in particular, standard ageing generally resulted in an increase in stress values, ranging from 13.25–19.46 MPa in severely aged samples to 24.03–25.18 MPa in standard-aged samples. This trend is consistent with the FTIR results, which suggested better preservation of the collagen structure in these samples.

This result can be attributed primarily to two closely related factors: the degree of thermomechanical degradation of the matrix and the stiffening effect induced by structural changes (such as thermal cross-linking) during less aggressive aging.

Here are the molecular and structural details that explain the phenomenon:

- Collagen integrity and preservation. FTIR data confirm that standard aging better preserves the triple helix and native structure of collagen compared to severe aging. In samples subjected to severe aging, high temperatures or extreme conditions cause collagen denaturation (partially transforming it into gelatin) and the breaking of peptide bonds. This degraded collagen is no longer able to effectively transfer and withstand mechanical loads, resulting in significantly lower stress (strength) values (13.25–19.46 MPa).
- Cross-linking phenomena induced by standard aging. During standard aging, the material undergoes a moderate, prolonged thermal process. This can promote the formation of intermolecular cross-links within the well-preserved collagen structure or lead to the loss of bound water (dehydration). Both phenomena reduce polymer chain mobility, increasing the material’s stiffness and tensile strength, which explains the jump in stress values to 24.03–25.18 MPa.
- Presence of structural defects and micro-cracks. Severe aging has a destructive effect, creating micro-voids, fissures, and zones of significant heterogeneity within the material. Under mechanical stress, these defects act as stress concentrators, leading to premature failure of the sample. In contrast, the homogeneous and protected structure resulting from standard aging distributes the load evenly.

In summary, the higher stress value observed under standard aging conditions is due to the fact that the collagen structural framework remained intact (as evidenced by FTIR) and became stabilized or stiffened, whereas severe aging literally dismantled the material’s molecular architecture, compromising its mechanical properties.

Nanocollagen generally produced an increase in elasticity and elongation at break in most of the samples, especially in samples treated with 2 g/L tannic acid. For example, in IA_2 g/L samples, strain increased from 46.49% to 83.06%, while elongation at break increased from 23.24 mm to 41.53 mm in untreated and Nanocollagen-treated samples, respectively. In contrast, more limited variations were observed in samples treated with 10 g/L tannic acid, where the values remained comparable to those of the untreated control. Klucel, on the other hand, showed more limited variations compared to Nanocollagen and, in most cases, exhibited values close to those of untreated samples. The only case in which this consolidating agent improved the properties compared to the untreated sample was observed for IA_2 g/L, with strain increasing up to 75.07% and elongation at break up to 37.54 mm, although the values were still lower than those obtained for Nanocollagen.

Colorimetric analysis further highlighted significant differences in the behaviour of the two treatments (Table 6). Nanocollagen induced extremely limited chromatic variations under all experimental conditions (ΔE* < 3), consistent with visually imperceptible colour changes. In contrast, Klucel G showed a more variable chromatic impact: although in most cases the ΔE* values remained below 3, the IA_2 g/L sample exhibited a ΔE* value of 5.22, exceeding the acceptability threshold and indicating a significant chromatic variation [34,35].

The results obtained on the laboratory samples also made it possible to evaluate the effectiveness of the consolidating treatments in relation to the different artificially induced degradation levels. FTIR analyses confirmed that the accelerated ageing protocol produced more pronounced alterations of the collagen matrix compared to the standard ageing protocol, consistent with the results of the mechanical investigations carried out on untreated samples. This difference can be attributed to the presence of HCl in the accelerated ageing treatment, which promotes hydrolytic degradation of collagen and reduces its structural stability. The two ageing procedures therefore enabled the preparation of samples characterized by different degradation levels, similarly to what was observed in the real samples. Among the consolidants analysed, Nanocollagen exhibited highly compatible features towards the original (ancient) leather samples. Microscopic and SEM observations indeed revealed preservation of the natural surface morphology of the leather and a better maintenance of the tridimensionality of the fibrillar structure, suggesting a homogeneous interaction between the treatment and the degraded collagen matrix. The effectiveness of Nanocollagen was further supported by mechanical tests, which demonstrated enhanced flexibility and elongation at break, particularly in moderately degraded samples. These results suggest that the treatment is capable of improving cohesion among collagen fibrils, contributing to the recovery of the natural elasticity of the support, in agreement with previous literature [14]. This effect may be associated both with the chemical compatibility of Nanocollagen with the proteinaceous matrix of the support and with the nanometric dimensions of the material, which favour deeper penetration within the degraded matrix and the ability to create links among fibrils. This enables widespread consolidation rather than an effect limited exclusively to the surface of the artefact, promoting a more effective reorganization of the collagen fibrillar structure. Conversely, Klucel G tends to form a more compact and continuous superficial film, particularly evident in UV and SEM observations, causing a flattening of the surface morphology without significantly modifying the internal structure of the material. Moreover, the mechanical parameters showed more limited and less homogeneous effects compared to those observed for Nanocollagen, with values frequently comparable to those of untreated samples. This experimental evidence suggests a predominantly superficial action of the consolidants, consistent with what has been reported in the literature for cellulose derivatives employed in the treatment of historical leather [16,24,75]. From an aesthetic point of view, Nanocollagen also induced extremely limited chromatic variations (ΔE* < 3) both in laboratory samples and in original fragments, thus proving fully compatible with the requirements for the treatment of historical artefacts. Klucel G, on the other hand, showed more heterogeneous chromatic variations; indeed, although remaining in most cases within limits still considered acceptable from a conservation perspective, in some samples it exceeded the ΔE* = 5 threshold, producing clearly perceptible chromatic variations above the tolerability threshold for treatments applied to historical artefacts. Overall, the obtained results confirm the potential of Nanocollagen as an innovative consolidating agent for collagen-based historical artefacts. Its high chemical affinity with the proteinaceous substrate, its ability to penetrate within the degraded matrix and its limited aesthetic impact make it possible to improve fibrillar cohesion and the mechanical properties of the support without significantly altering its surface morphology. These characteristics make Nanocollagen a valid alternative to Klucel G for the conservation and restoration of historical leather artefacts (the latter studied in the next Section 3.3).

### 3.3. Original Samples: Conservation of Historical Leather Samples

The results collected for the laboratory samples allowed the Nanocollagen application (and Klucel G for comparison) on the original samples, which, due to their small size, were analysed only by SEM-EDS and spectrocolorimetric techniques. For SEM-EDS characterization, Bin. 1 was selected because it proved more stable for SEM observation. In fact, Bin. 5 was powdery and tended to literally crumble apart (as soon as it was handled to prepare the fragment for the sample holder, or stub). If the material loses structural cohesion, analysis becomes impossible because the sample would disintegrate before or during the test (also causing issues with the high-vacuum environment). On the other hand, sample B.6 showed clear evidence of biological damage. In the presence of biodeterioration (as in this case), with the surface of sample B.6 completely covered by established biological contaminants, the SEM electron beam images only the biological deposit rather than the actual morphology of the leather fibers. In the case of sample Bin. 5, the ancient, mechanically fragile leather exhibits very high porosity and extremely hydrolyzed and fragmented collagen. This type of structure tends to absorb the Nanocollagen like a sponge; however, because the advanced state of degradation prevents uniform bonding, the excess consolidant deposits on the surface, creating a continuous, amorphous, glassy film. This “coating” levels out and obliterates all micro-roughness and the native topography of the fibers. Furthermore, if the organic polymer is not perfectly integrated or forms an excessively thick layer, the charging effect increases drastically: electrons from the SEM beam become trapped in the insulating Nanocollagen layer, causing glare, distortion, and a total loss of image contrast. Biofilm is not merely surface dirt; it is a hygroscopic extracellular polymeric substance (EPS) matrix produced by microorganisms on Bin. 6, rich in retained water and salts. Nanocollagen applied over this layer (Bin. 6) could not reach the actual leather beneath; instead, it reacted both chemically and physically with the biofilm biomass. The Nanocollagen “sealed” and encapsulated the biofilm. When the sample was placed in the SEM vacuum chamber, residual moisture trapped beneath the new nano-collagen film tended to collapse, causing micro-explosions, swelling, or distortion of the film itself. Furthermore, the disordered interaction between the consolidant’s nano-proteins and the biofilm’s organic matter creates a heterogeneous surface that is chemically unstable under the electron beam, thereby degrading the resolution of the SE (Secondary Electron) parameters.

**Figure 7:**
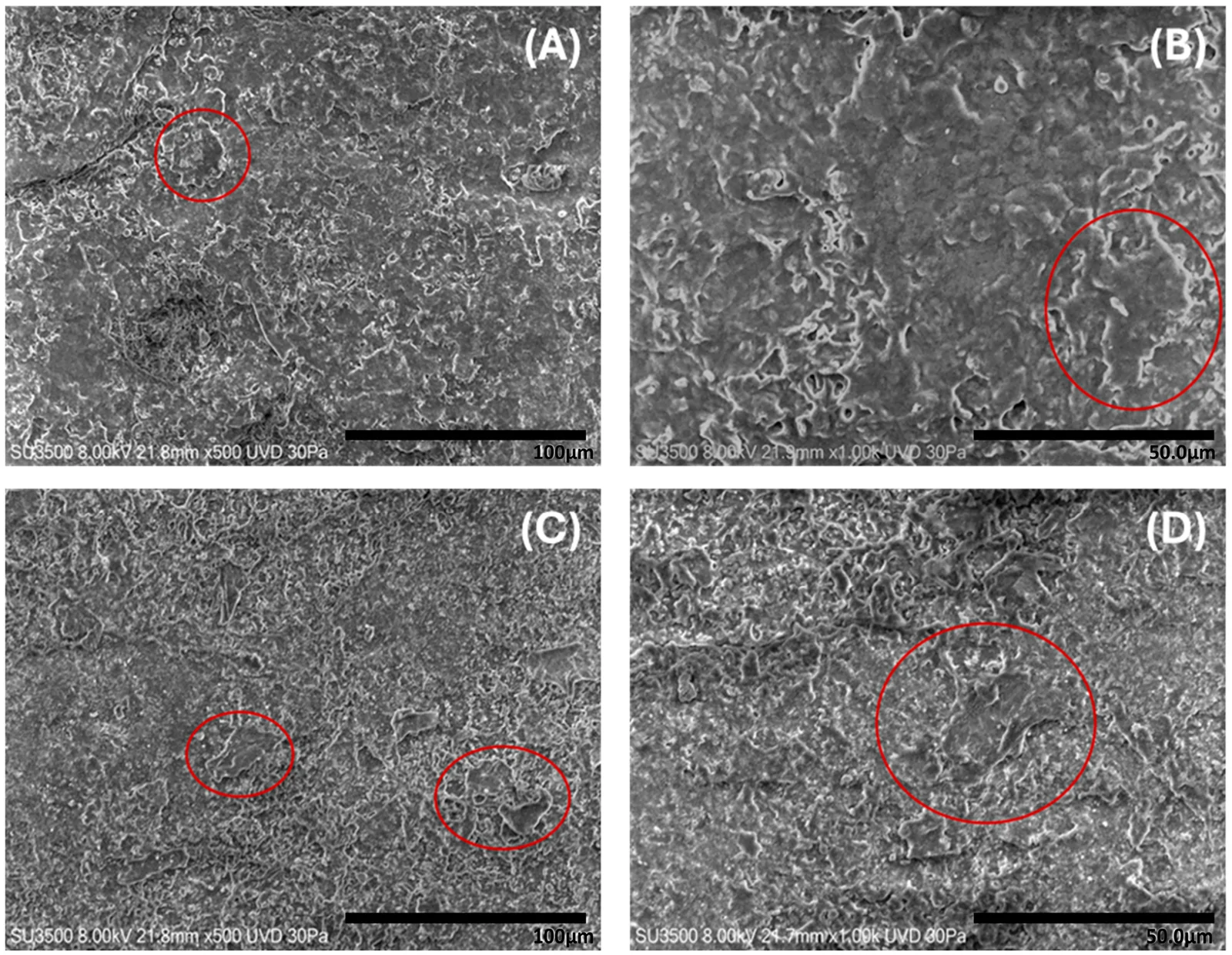
SEM micrographs acquired from Bin. 1 after: Klucel G at 500× (**A**) and 1000× magnification (**B**); Nanocollagen at 500× (**C**) and 1000× magnification (**D**). The survey data were of better quality for Bin 1 than for Bins 5 and 6, as the latter exhibited extreme fragility and susceptibility to breakage (Bin 5) and surface contamination due to microorganisms (primarily Bin 6). The technical and experimental reasons for analyzing only the original Bin 1 sample have been described in detail in the text above. Bin. 1: Original Samples collected from Binding 1.

SEM micrographs in Bin. 1 revealed significant differences between the two treatments (Figure 7). In samples treated with Klucel, the surface of the bindings appeared flattened and lacking tridimensionality, as visible from the margins of the areas highlighted in red. In contrast, the original samples treated with Nanocollagen showed a more natural appearance, preserving the surface details and the natural tridimensionality of the leather structure, as evidenced by the areas highlighted in red.

Concerning spectrocolorimetric analysis, the results confirmed information previously acquired/collected in the presence of laboratory samples (Table 7). In all the analysed bindings, Nanocollagen treatment induced limited chromatic variations, having ΔE* values below the perceptibility threshold. Instead, Klucel G showed a more variable effect especially in the case of Bin. 5, which exceeded the limit of 3 (visible chromatic variation), while still remaining within the acceptability range [34,35].

The consolidation performed on the original samples using Nanocollagen yielded interesting results that could open the way for future studies to be performed on other ancient leather samples affected by more advanced stages of red rot phenomena.

In light of the foregoing, it is appropriate to present the experimental results obtained from measurements designed to determine the efficacy of the consolidation treatment based on the new Nanocollagen. Since the aim of this further study was to understand how the material degrades in its entirety over time, it is ideal to perform measurements on both sides (hair and flesh) to compare the resistance of the hair layer (grain) against that of the fibrous layer (flesh). This is relevant to the assessment of consolidation Efficiency (%) exhibited by Nanocollagen treatments over time.

While in previous studies on high-rigidity stone matrices [35] the efficiency of the nanostructured treatment followed a logic of purely incremental mechanical and water-barrier properties, the case of organic library materials at the “Orientale” library in Naples necessitated a reformulation of the algorithm. Historical leather requires a balanced index in which structural cohesion is weighed against the preservation of flexibility and constrained by a strict chromatic tolerance penalty (ΔE). With historical leather, however, consolidation is a balancing act. An excessive increase in mechanical rigidity or a noticeable color shift signifies a treatment failure. Overall efficiency must therefore be a balanced, multi-parameter index. Here is how we can structure the Global Consolidation Efficiency Index formula (I_CE_), specific to the leather from the “Orientale University” in Naples:

$$I_{\mathrm{CE}\;}=\;w_{1}\;\times \;E_{\mathrm{Cohesion}}\;+w_{2}\times \;E_{\mathrm{Elasticity}}+\;w_{3}\;\times \;P_{\mathrm{Aesthetics}/\mathrm{Color}}$$

where w_1_, w_2_, w_3_ are the weighting coefficients (the sum of which must equal 1) that determine the relative importance of each parameter based on the initial state of degradation of the Art-Work objects. The structure of the individual parameters is detailed in the Experimental paragraphs. At Time Zero (immediately after the first consolidation treatment, carried out on Bin. 1 original sample), the values of the individual parameters and the global index are as follows:

- Cohesion (E_Cohesion_): 90% (crumbling is almost completely prevented);
- Elasticity (E_Elasticity_): 95% (the leather remained very soft and flexible);
- Color (ΔE): 1.2 (The shift is less than 2, so it is invisible. The (P_Aesthetic\Color_) penalty is 0.

The calculation of the global Consolidation Efficiency Index therefore results in I_CE_ = 93.5%, which demonstrates that the treatment was perfectly successful (at Time Zero). The same algorithm was applied two years after the initial consolidation treatment (specifically, the procedure involved assessing what is known as the aging settlement on Bin. 1 sample). The results are shown below:

- Cohesion (E_Cohesion_): 88% (Minimal drop: Nano-collagen still holds the fibers firmly together);
- Elasticity (E_Elasticity_): 85% (The leather has settled, showing very slight stiffening that is entirely safe for the movement of the cover);
- Color (ΔE): 1.8 (The color changed very slightly, remaining below the critical threshold of 2). The human eye does not perceive any spots or bleaching. The (P_Aesthetic\Color_) penalty remains 0.
- The calculation of the global Consolidation Efficiency Index therefore results in I_CE_ = 85.9%.

All these consolidation efficiency results are summarized in Table S7 in the Supplementary Materials for Bin. 1 original sample and laboratory sample (IA_2 g/L) for both sides: flesh and hair/grain. This two-dimensional approach is necessary because the two sides of the same ancient leather possess completely different structural, chemical, and degradation properties, given that the flesh side is both less exposed to environmental damage and less handled by end-users. A brief summary is provided below regarding the quantitative assessment of the consolidation treatment efficiency (%), including its performance over time (to investigate the effects of post-treatment aging).

Application of the Modified Global Efficiency Index (I_CE_) enabled an assessment of the long-term sustainability of the nanostructured treatment applied to historical leather bindings at the library of the “Orientale” University of Naples. At Time Zero, the treatment demonstrated excellent efficiency (I_CE_ = 93.5%), driven by the outstanding preservation of elasticity and the absence of chromatic alterations (ΔE* < 2). After a two-year monitoring period, the index showed a very slight, expected decline, settling at 85.9%. This value falls well within the acceptable range for conservation purposes. The most significant finding is the absence of aesthetic drawbacks: the ΔE* stabilized at 1.8, confirming that the Nanocollagen did not cause surface migration or blanching. Furthermore, the retention of 85% of elastic efficiency rules out the risk of delayed collagen stiffening, demonstrating the treatment’s long-term stability and compatibility with the flexibility requirements of book bindings.

In the future, this integrated sensor platform could serve as a model, particularly in the field of cultural heritage (though also in environmental and medical applications), for the accurate monitoring of key parameters such as mechanical properties, adhesion forces, pH, and optical properties or color. Regarding the latter, the ability to use portable CIELab spectrophotocolorimeters, combined with potentiometric probes (integrated ion-selective sensors) for measuring pH, temperature, and saline conductivity (the last of which monitors saline efflorescence and subflorescence), represents an essential first step toward developing mobile or portable laboratories. These would enable integrated sensors to operate directly “in situ”, a capability that is crucial for immovable Cultural Heritage (in loco integrated sensors mobile laboratory for monitoring the condition of Art-Works, both indoors and outdoors Cultural Heritage).

## 4. Conclusions

This work investigates the potential role of Nanocollagen as a consolidating agent towards the conservation of historical leather bookbindings (comparing with the conventional Klucel G agent). This study is original because, for the first time, it provides detailed and accurate scientific information regarding the state of preservation, raw materials, and treatments of historical artifacts from the University of Naples “L’Orientale.” However, it was not possible to do the same for dating, as the items consisted of binding fragments or detached bindings, making it impossible to identify their period of production.

The multi-analytical approach was applied to both laboratory samples and non-relocatable original fragments, the latter collected from three historical bookbindings preserved at the Library of the University of Naples “L’Orientale”. Results provide a complete characterization of the constituent (raw) materials, the treatments that the raw materials have undergone over time, and their state of conservation. Experimental data (acquired prior to the application of the proposed consolidation treatments) revealed a compositional homogeneity among the bookbindings combined with a main degradation effect, firstly related to the fragmentation of collagen fibres and the loss of matrix cohesion. No significant evidence of oxidation and/or acidification processes was observed on the same samples. Once this information was obtained, treatments based on Nanocollagen and Klucel G were applied to laboratory samples that replicated the various degrees of damage previously observed on the original samples (such as non-repositionable fragments).

The comparison between the two treatments showed that Nanocollagen exhibits greater compatibility with the collagen-based substrate than Klucel G. The treatment preserved the surface morphology of the leather, limited colour variations, and improved the mechanical properties of the degraded samples, particularly in the case of moderate degradation. By contrast, Klucel G showed a predominantly superficial action, associated with lower effectiveness in improving the mechanical properties of the degraded material and, in some cases, with more pronounced colour changes.

Overall, the results confirm the potential of Nanocollagen as a valid alternative to Klucel G for the consolidation of historical collagen-based artefacts. Its high affinity with the substrate, the limited colour variations associated with the treatment, and its ability to improve the cohesion of the degraded matrix highlight its potential for future applications in the conservation and restoration of library heritage (also featuring more advanced red rot effects).

After a two-year monitoring period, the index showed a very slight, expected decline, settling at 85.9%. This value falls well within the acceptable range for conservation purposes. Furthermore, the retention of 85% of elastic efficiency rules out the risk of delayed collagen stiffening, demonstrating the treatment’s long-term stability and compatibility with the flexibility requirements of book bindings.

The potential to develop a platform of integrated sensors—some of which are portable or mobile—undoubtedly paves the way for a new concept of sensors and smart devices for monitoring Cultural Heritage, including “in situ” applications. This aspect offers performance and versatility not only for immovable Art-Works but also for other compelling fields such as Telemedicine, Environmental Pollution monitoring and numerous industrial sectors.

## Figures and Tables

**Table 1:** Optical microscopy of original samples in VIS and UV light. Magnification: 60× (scale bar: 1.0 mm) and 180× (scale bar: 0.2 mm). Bin. 1: Original Samples collected from Binding 1; Bin. 5: Original Samples collected from Binding 5; Bin. 6: Original Samples collected from Binding 6.

Sample	50×		180×	
	VIS	UV	VIS	UV
**Bin. 1**	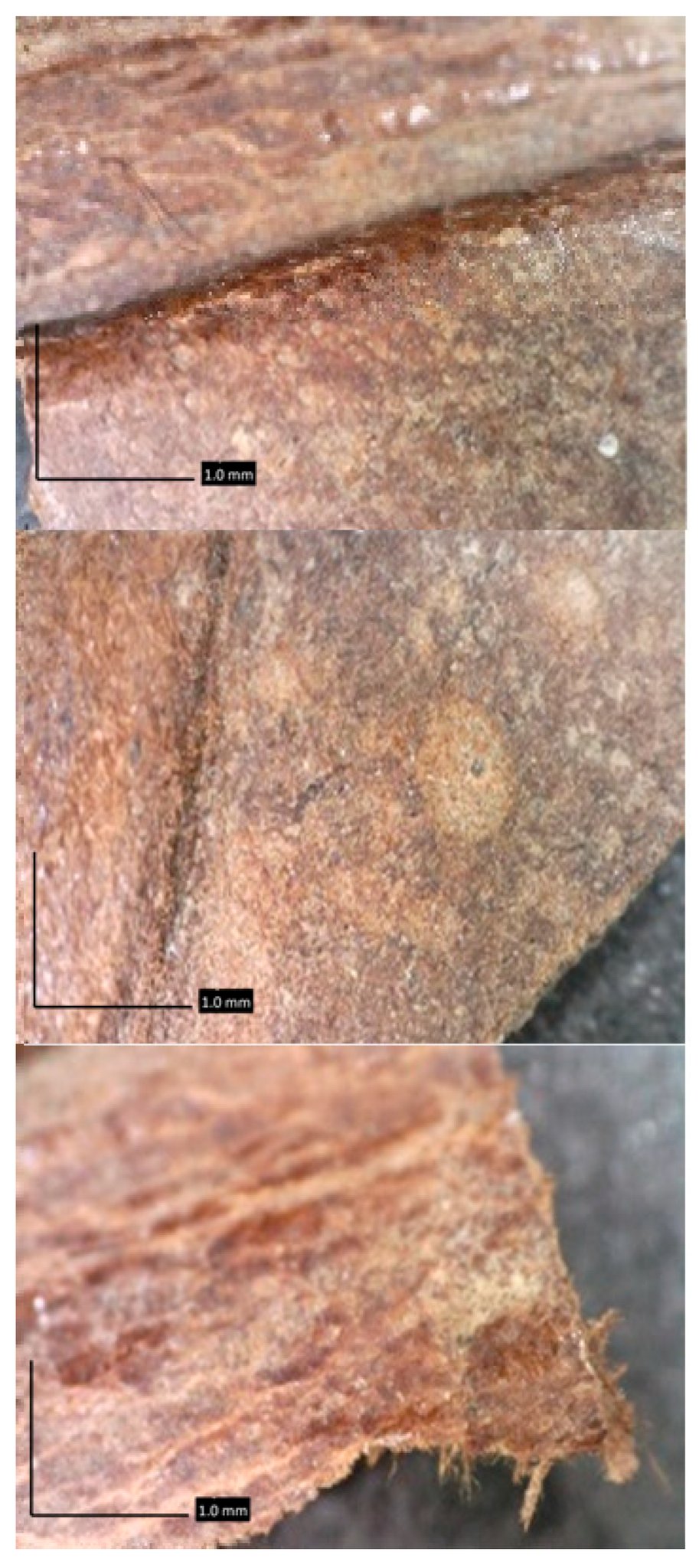	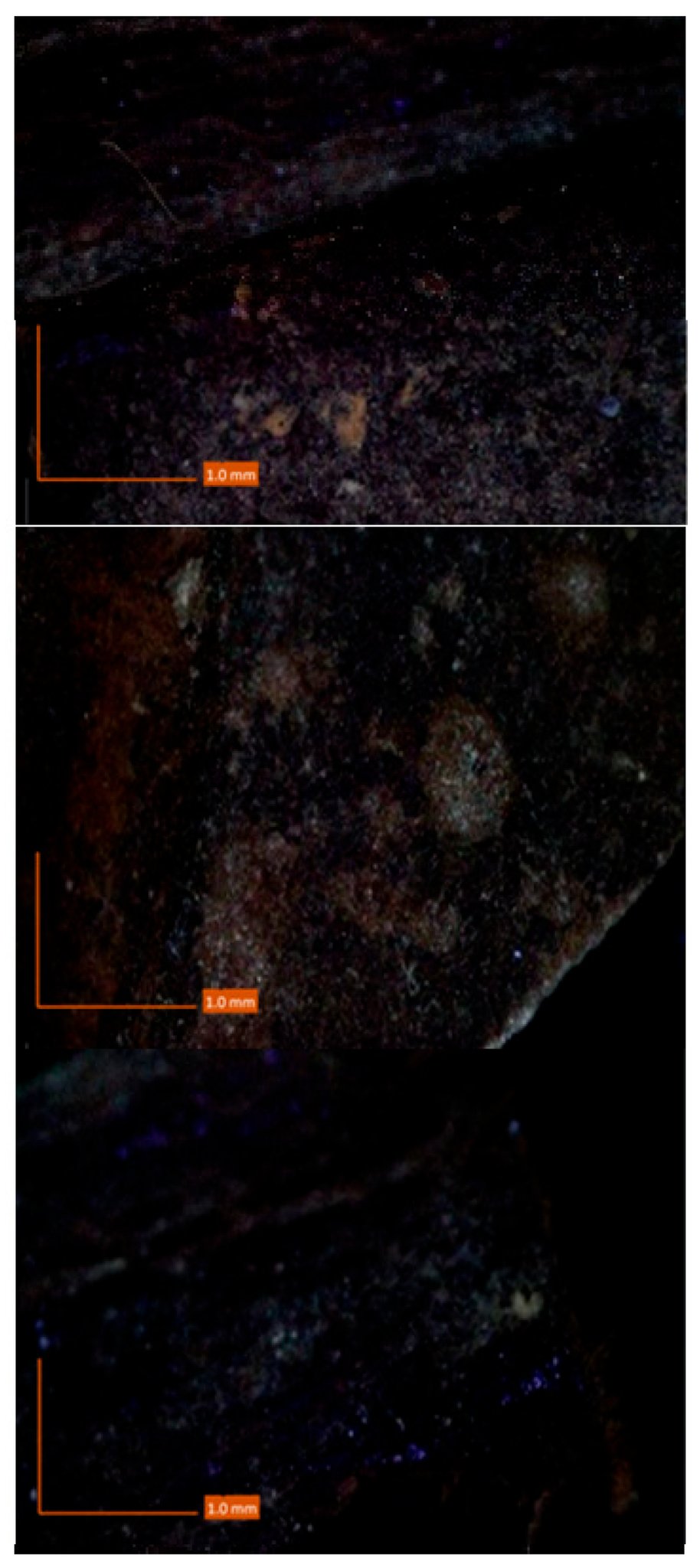	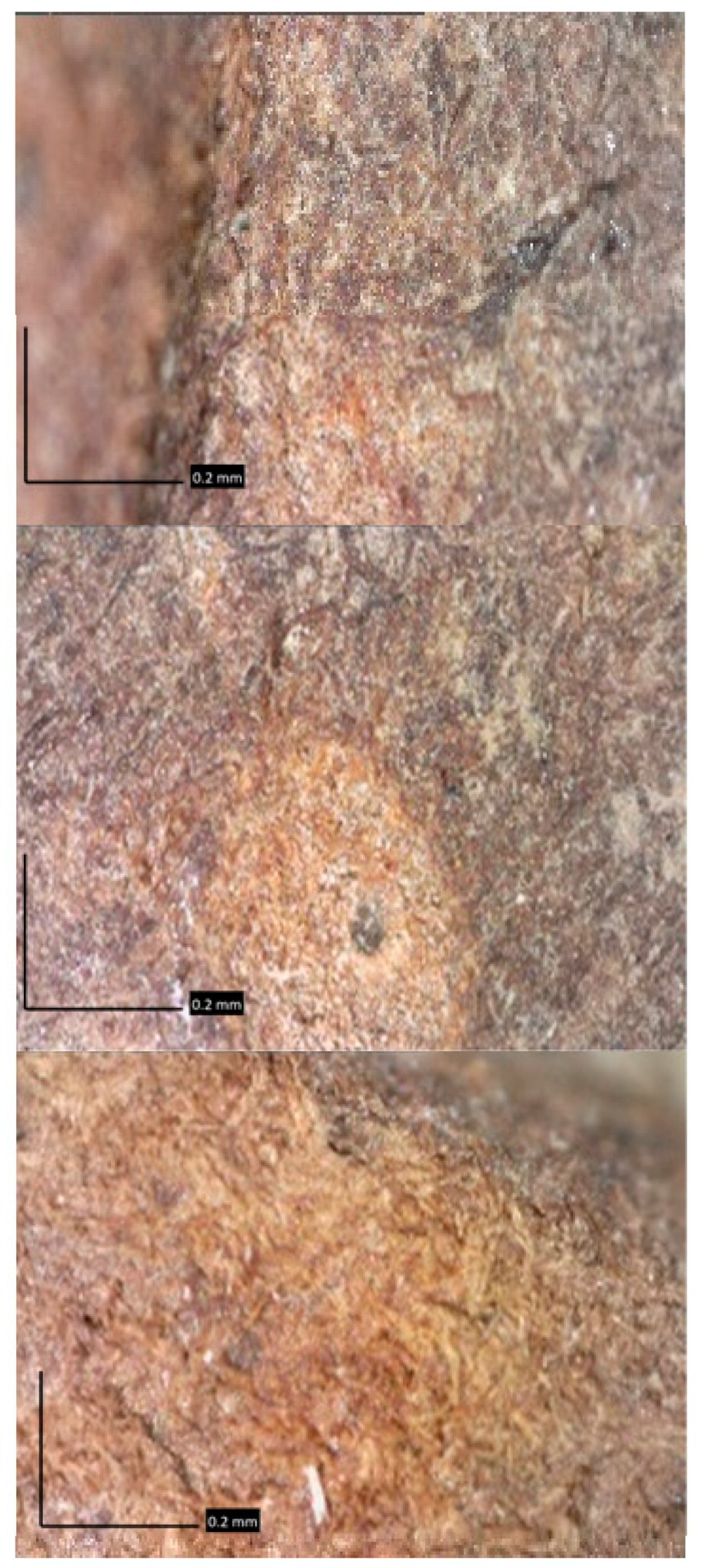	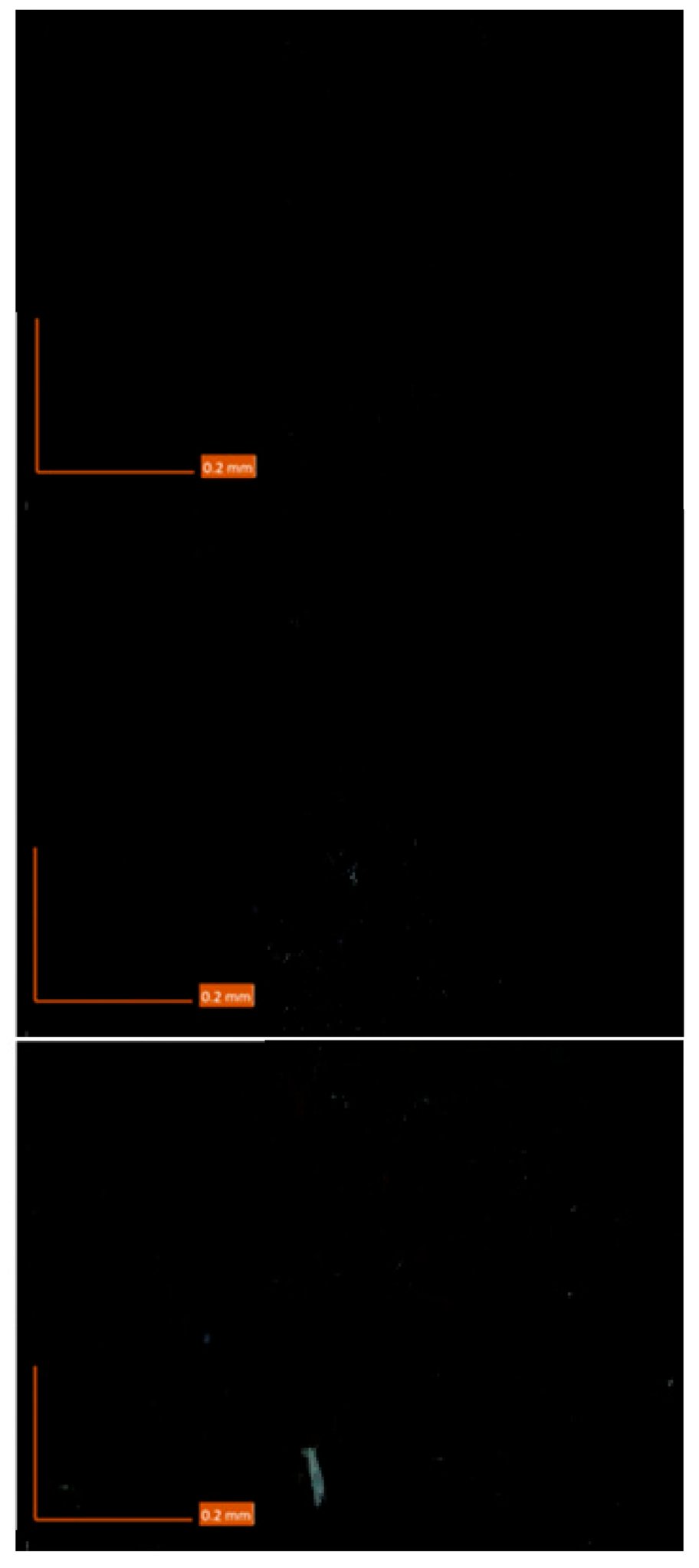
**Bin. 5**	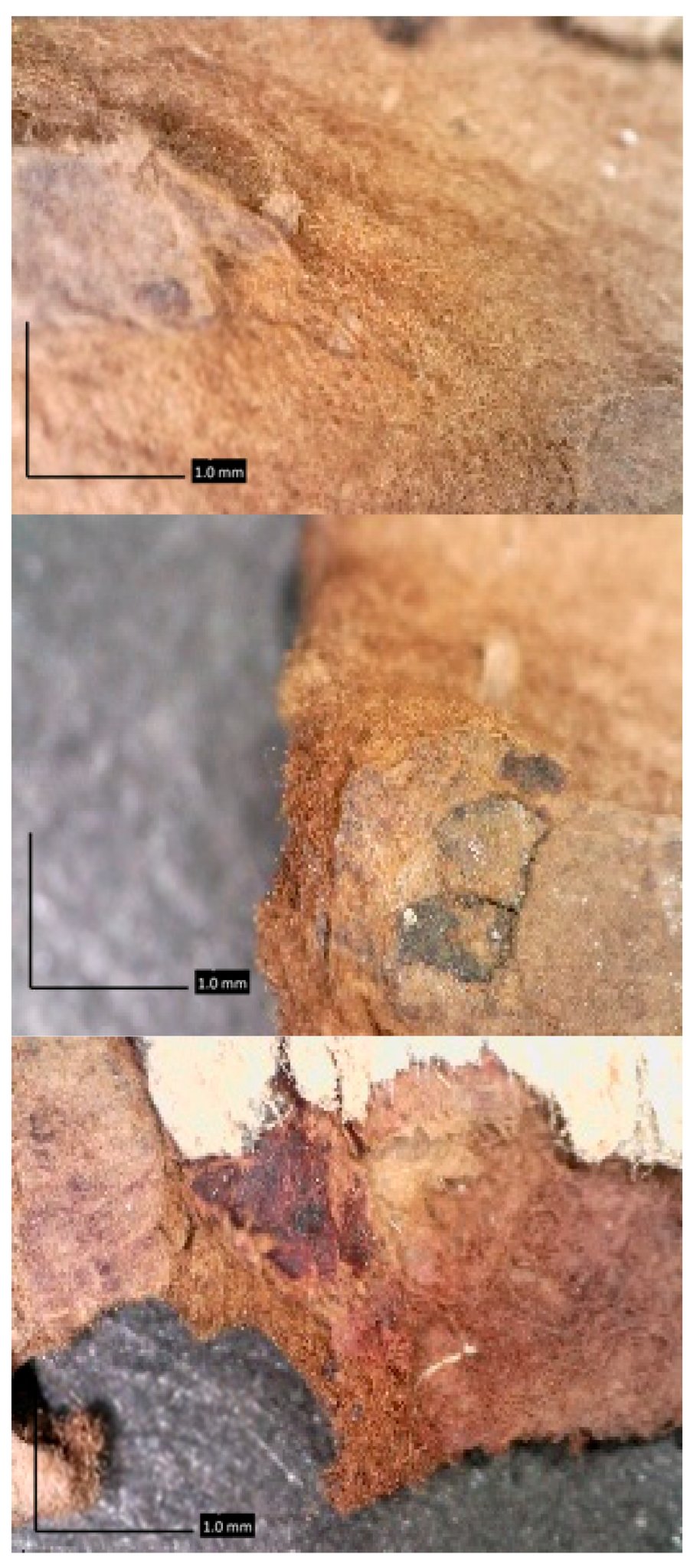	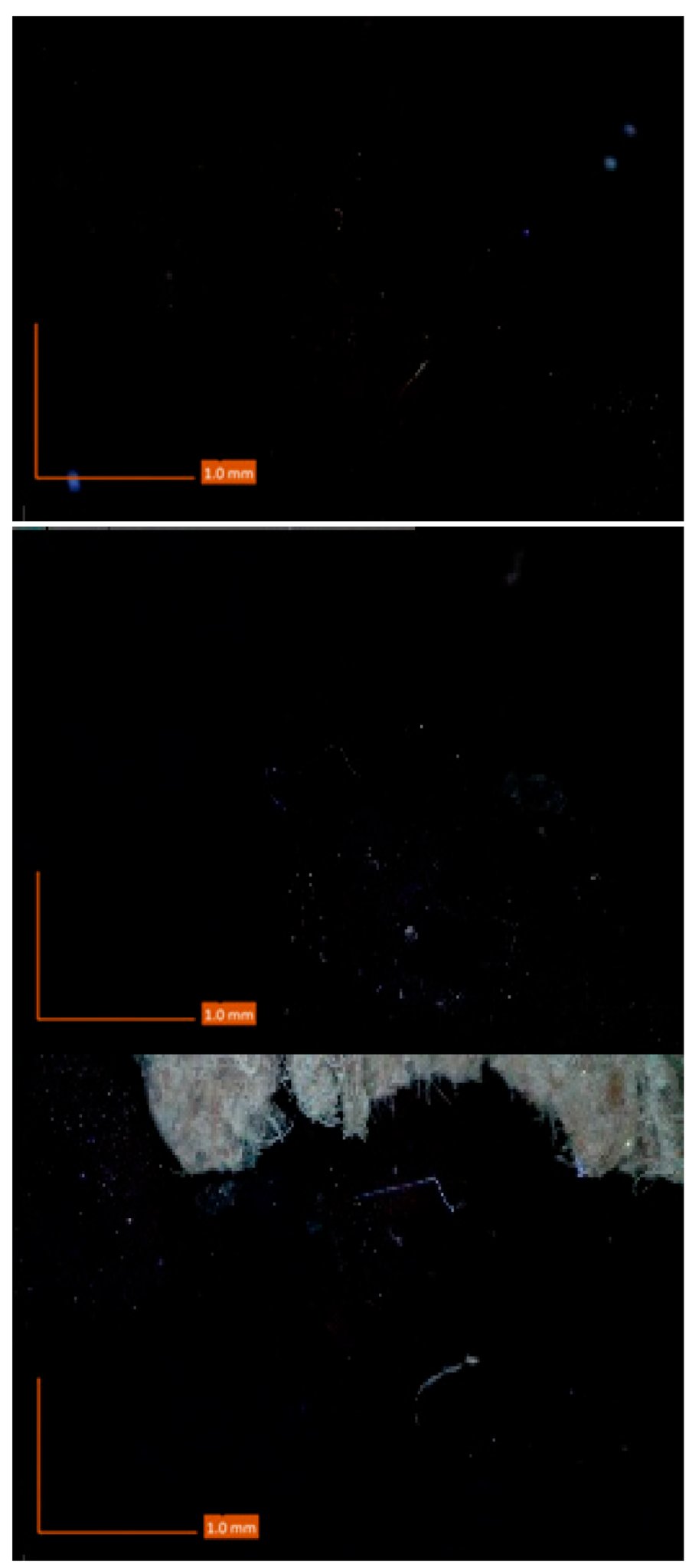	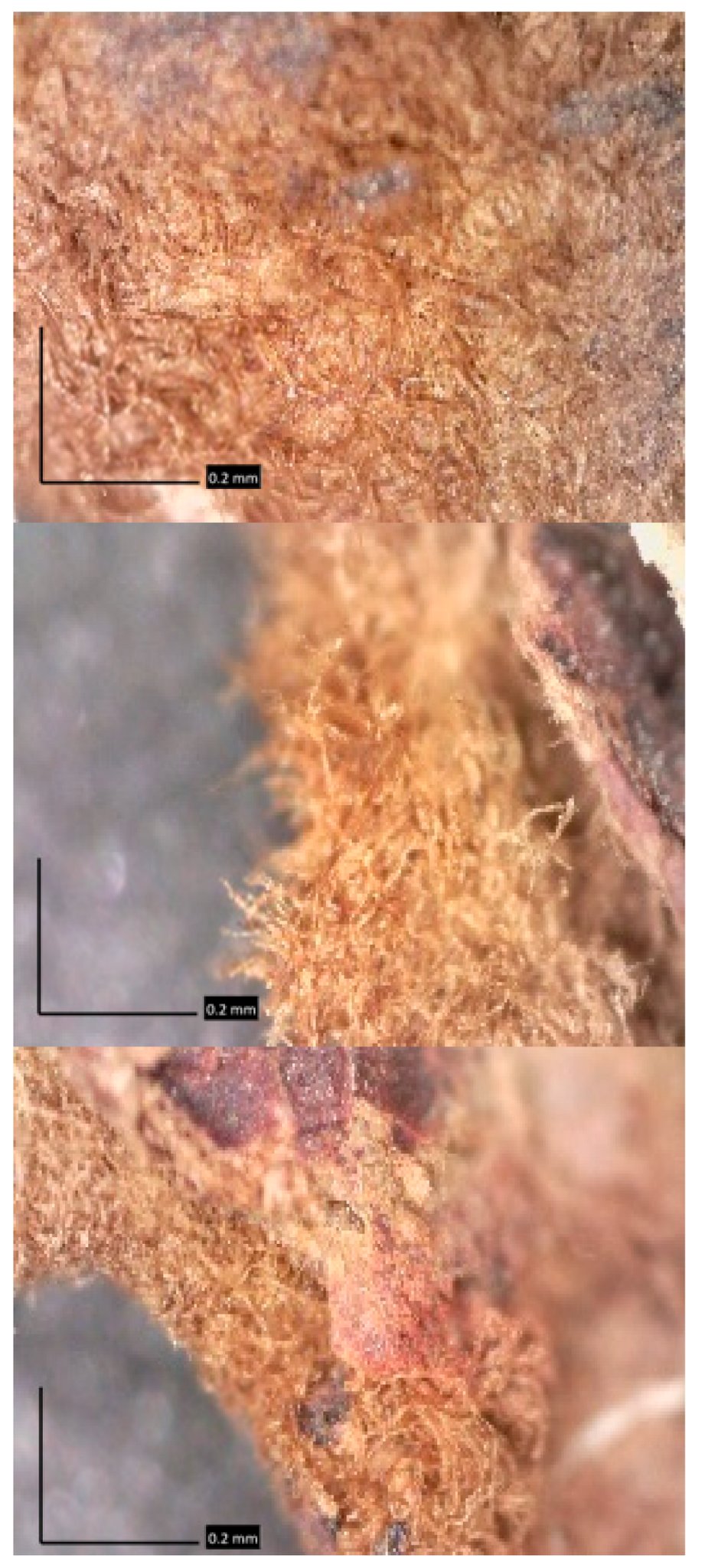	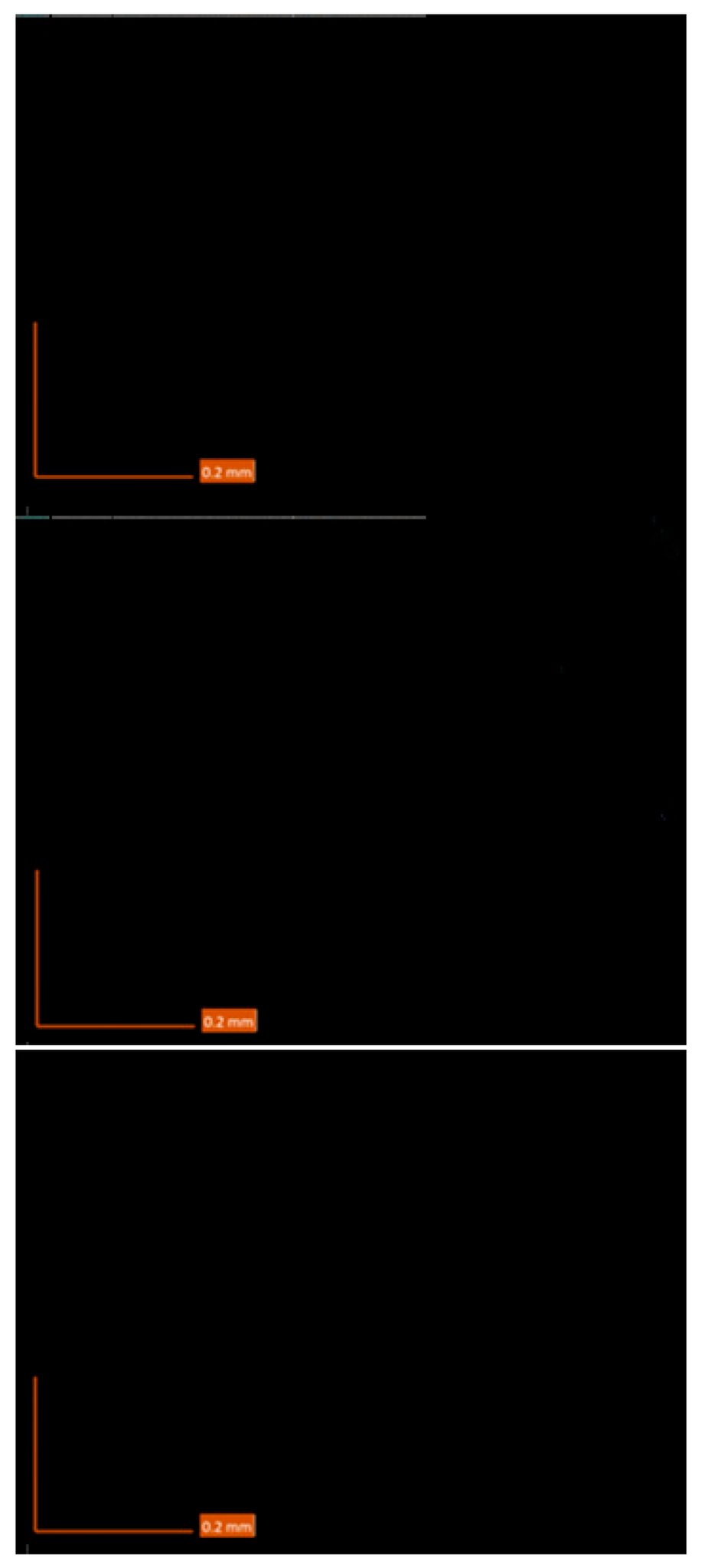
**Bin. 6**	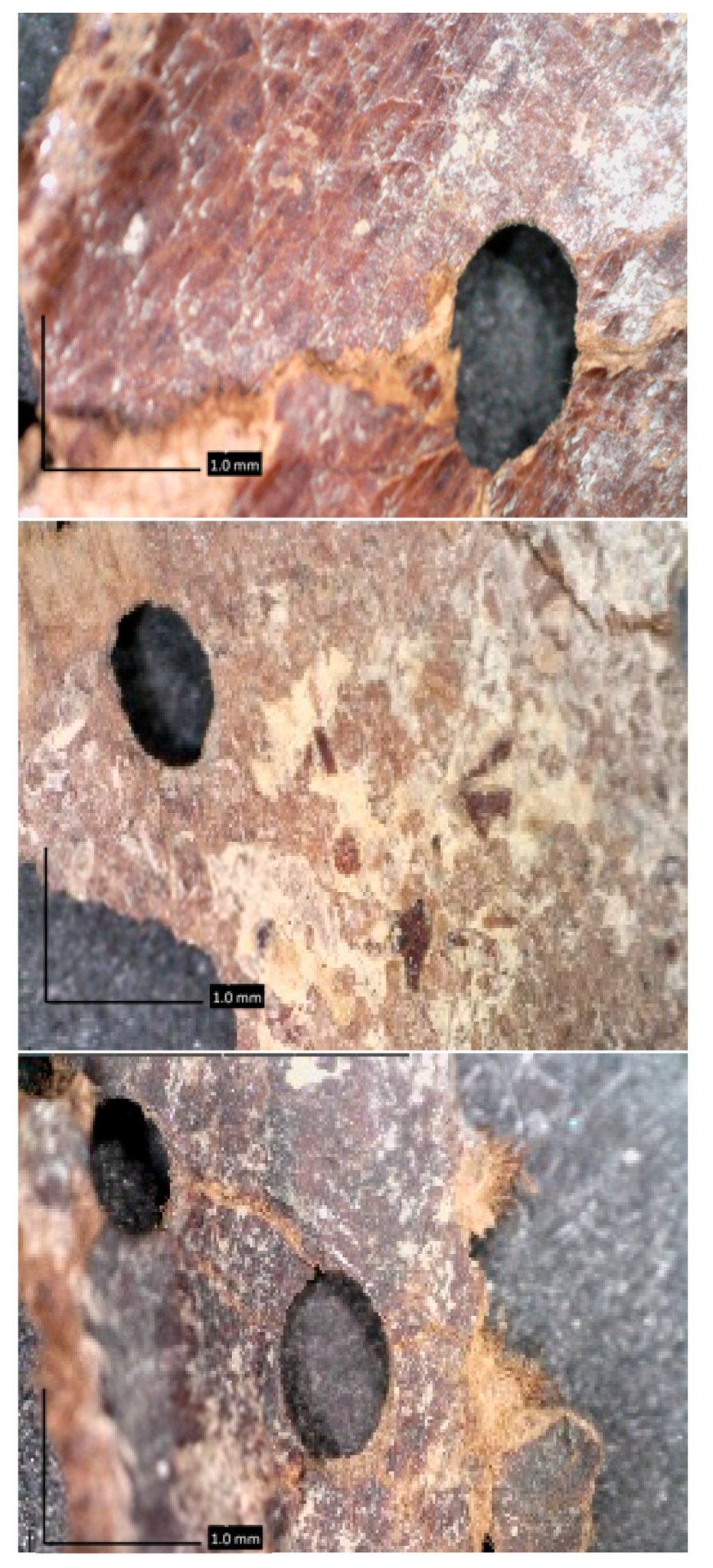	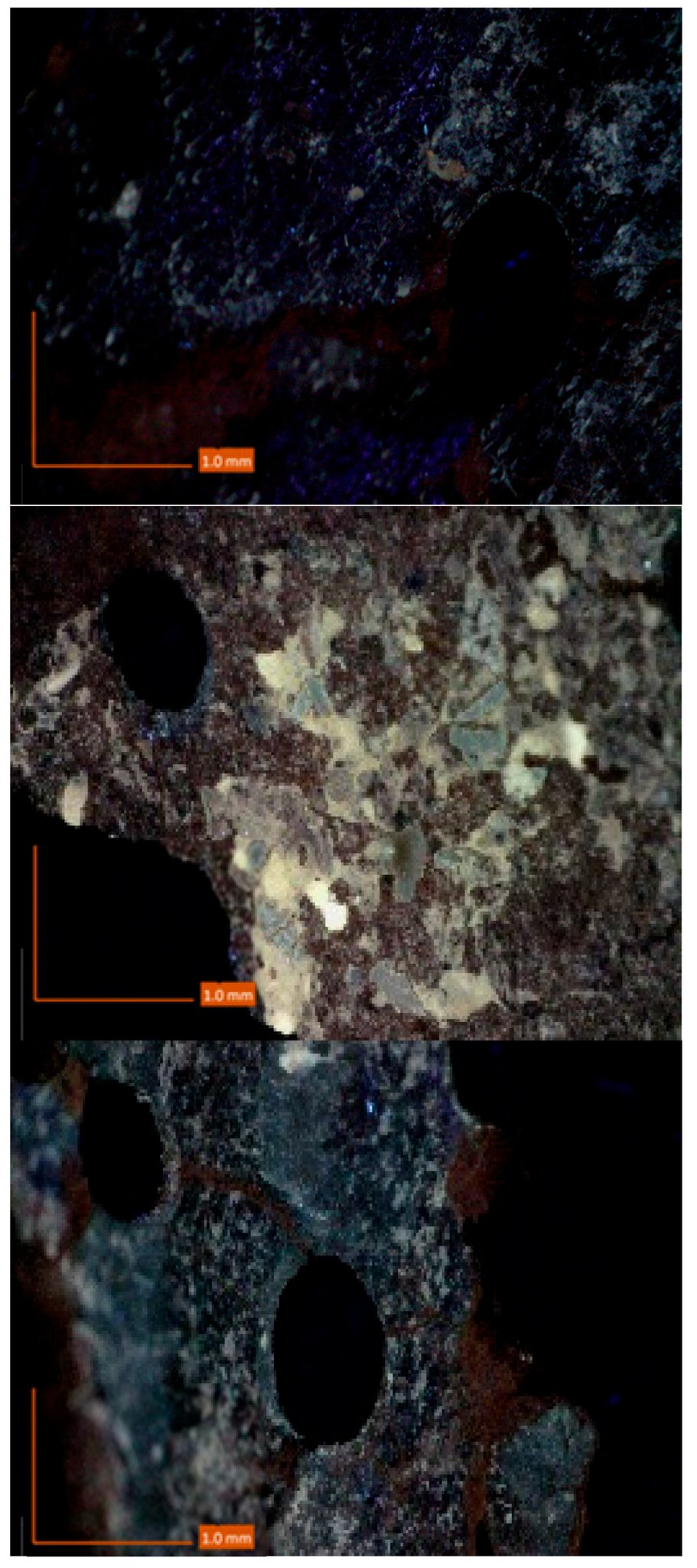	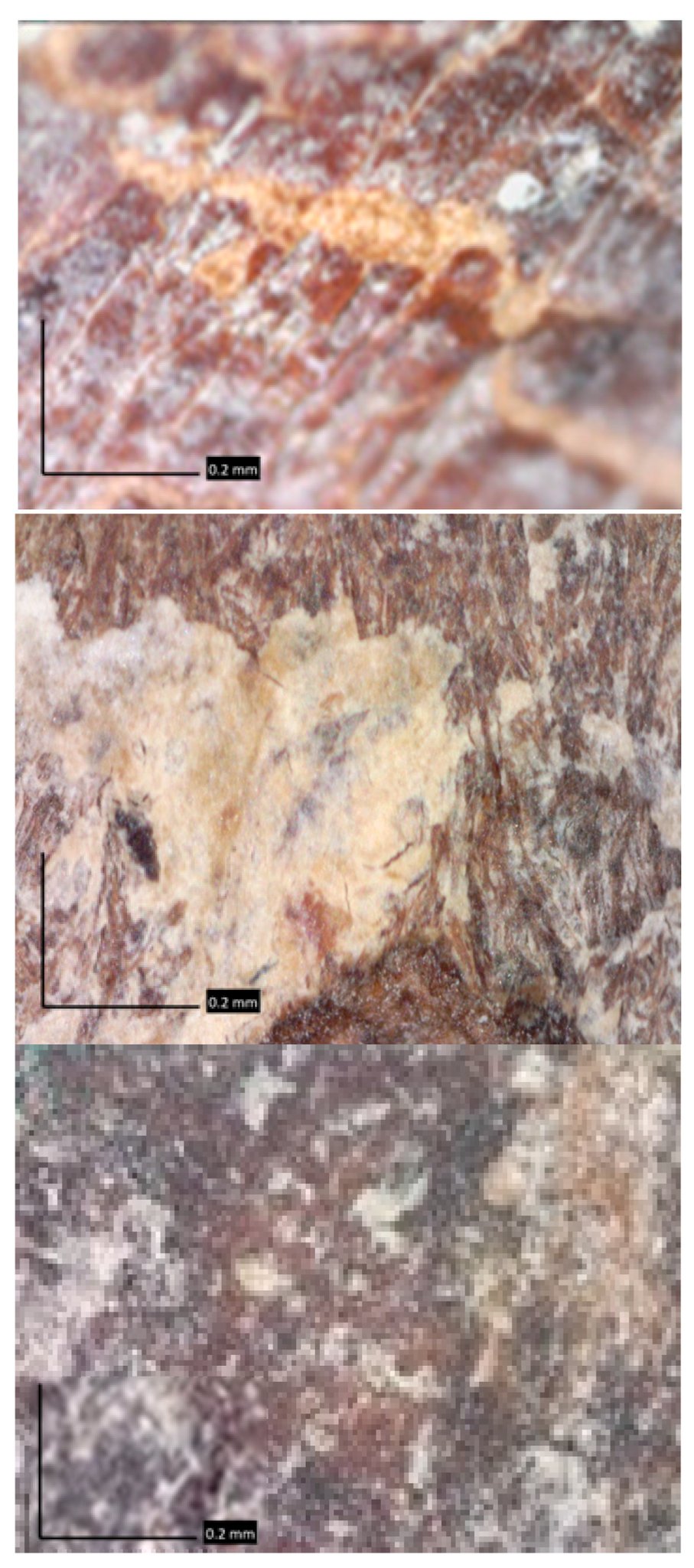	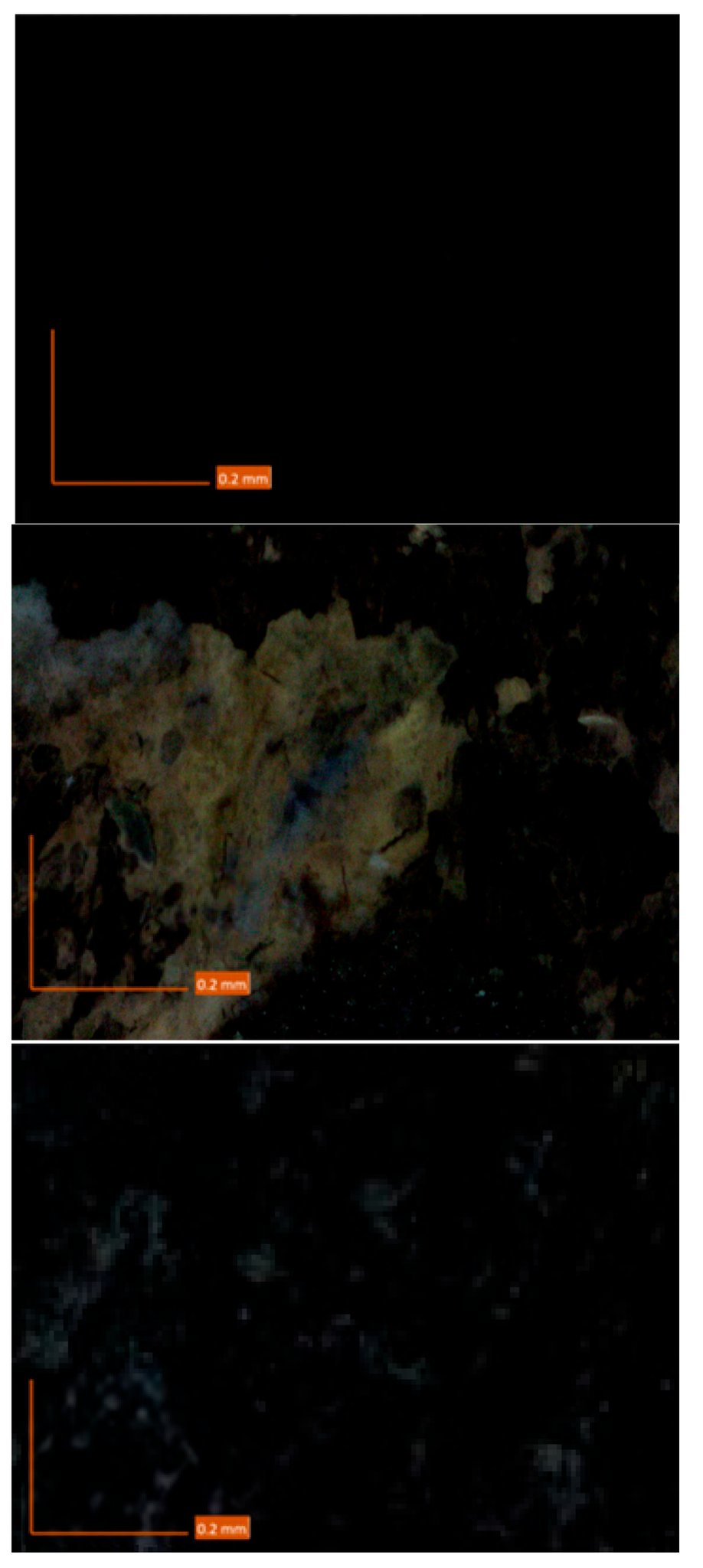

**Table 2:** Summary table of pH measurements and qualitative tests performed on the original samples. Bin. 1: Original Samples collected from Binding 1; Bin. 5: Original Samples collected from Binding 5; Bin. 6: Original Samples collected from Binding 6.

Sample	pH Measurement		Qualitative Chemical Tests			
	pH	ΔpH	FeCl_3_	Rhodamine	Butanol-HCl	Alizarin
Bin. 1	6.50	0.11	+	−	+	+
Bin. 5	6.40	0.20	+	−	+	+
Bin. 6	6.29	0.14	+	−	+	+

The distilled water used for the pH measurements had a pH value of 5.27. +: positive; −: negative.

**Table 3:** Raman and FTIR spectral parameters as indicators of the conservation state of leather samples. Bin. 1: Original Samples collected from Binding 1; Bin. 5: Original Samples collected from Binding 5; Bin. 6: Original Samples collected from Binding 6.

Parameters	Analytical Technique	Bin. 1		Bin. 5		Bin. 6		Value	Ref.
		Hair Side	Flesh Side	Hair Side	Flesh Side	Hair Side	Flesh Side		
I_AI_/I_AII_	FTIR	1.55	1.09	1.35	1.26	1.14	1.22	>1 Hydrolysis	[60,61]
I_AI_/I_AIII_	Raman	2.86	2.25	1.36	1.27	1.15	1.23	>2 Structural order-disorder transitions	
Δ(ν_AI_–ν_AII_) (cm^−1^)	FTIR	83.82	73.82	83.89	83.82	84.37	87.67	>95 Denaturation	
1720–1740 (cm^−1^)	FTIR	-	-	-	-	-	-	Oxidation	

-: band not detected.

**Table 4:** Optical microscopy images of laboratory samples before and after ageing under VIS and UV illumination (IA: severe ageing; INA: standard ageing; 2 g/L tannic acid; 10 g/L tannic acid). Images were acquired at 60× (scale bar: 1.0 mm) and 180× magnification (scale bar: 0.2 mm).

Sample		50×		180×	
		VIS	UV	VIS	UV
Before aging		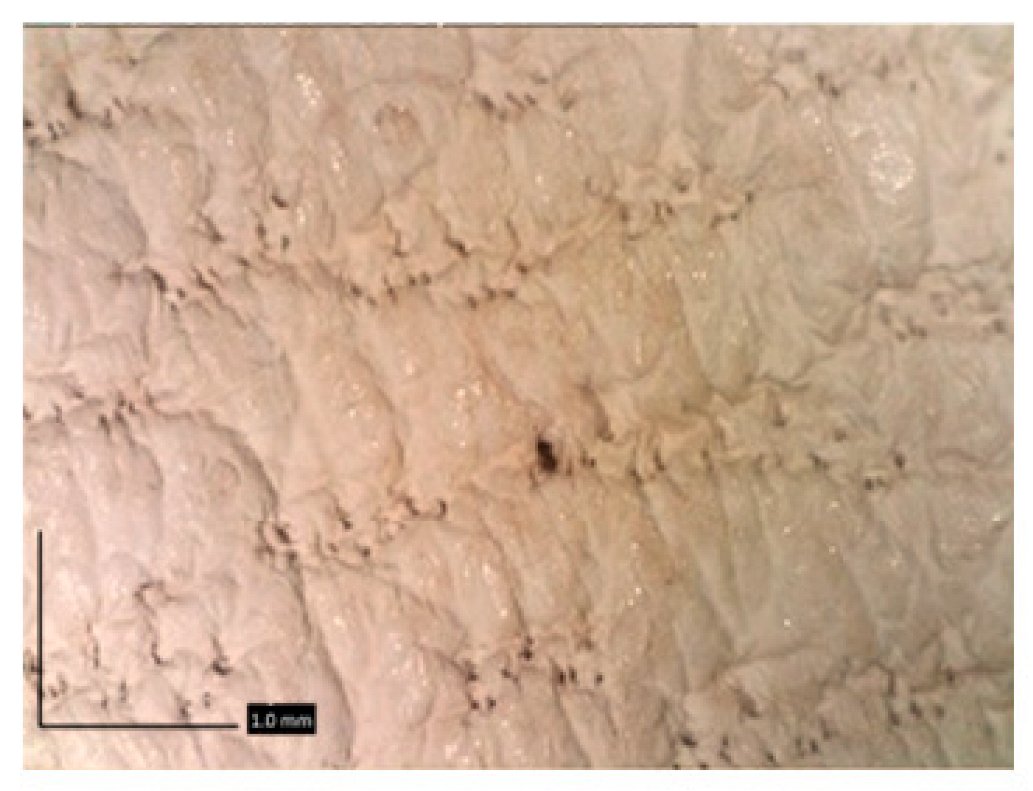	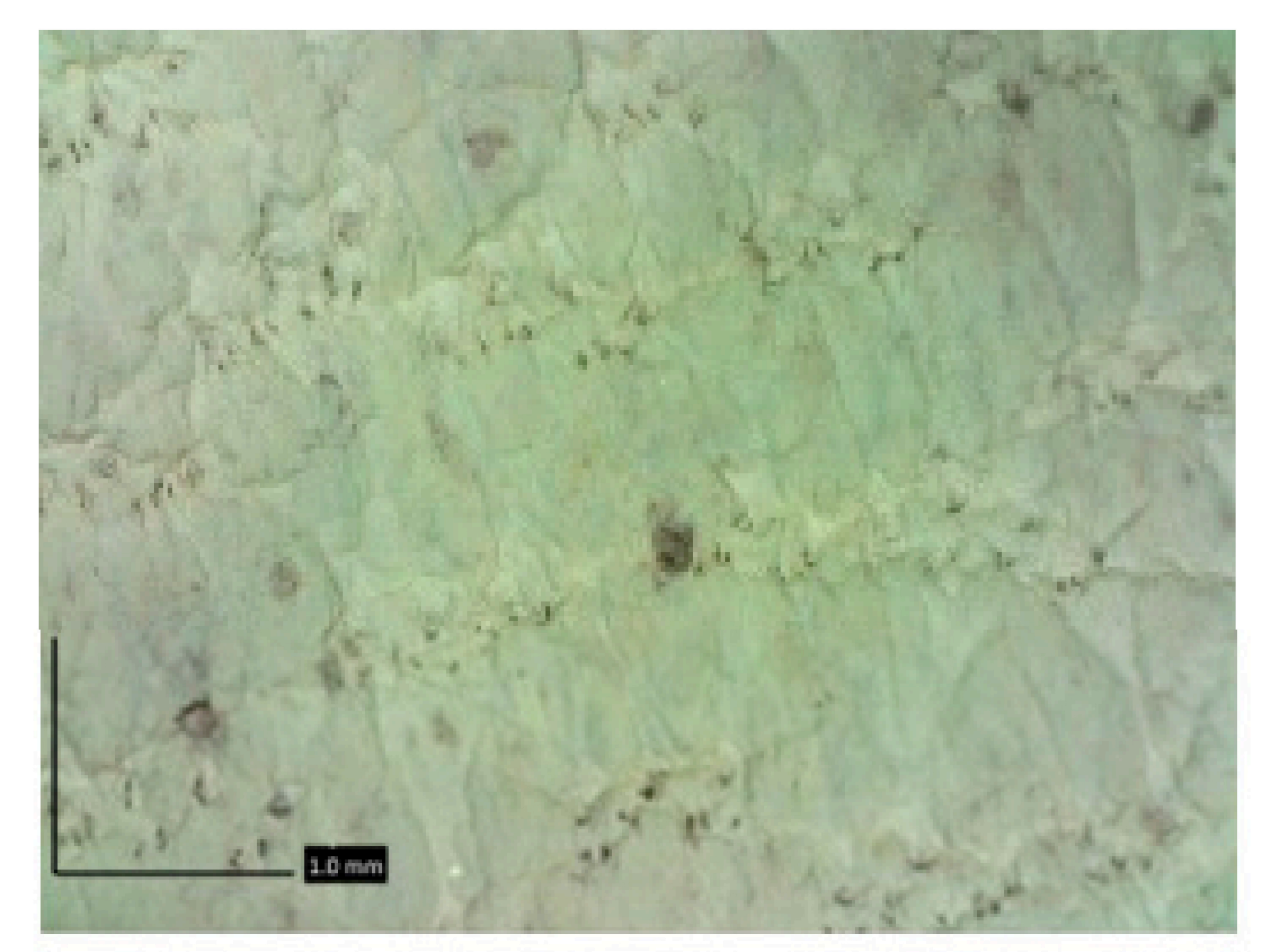	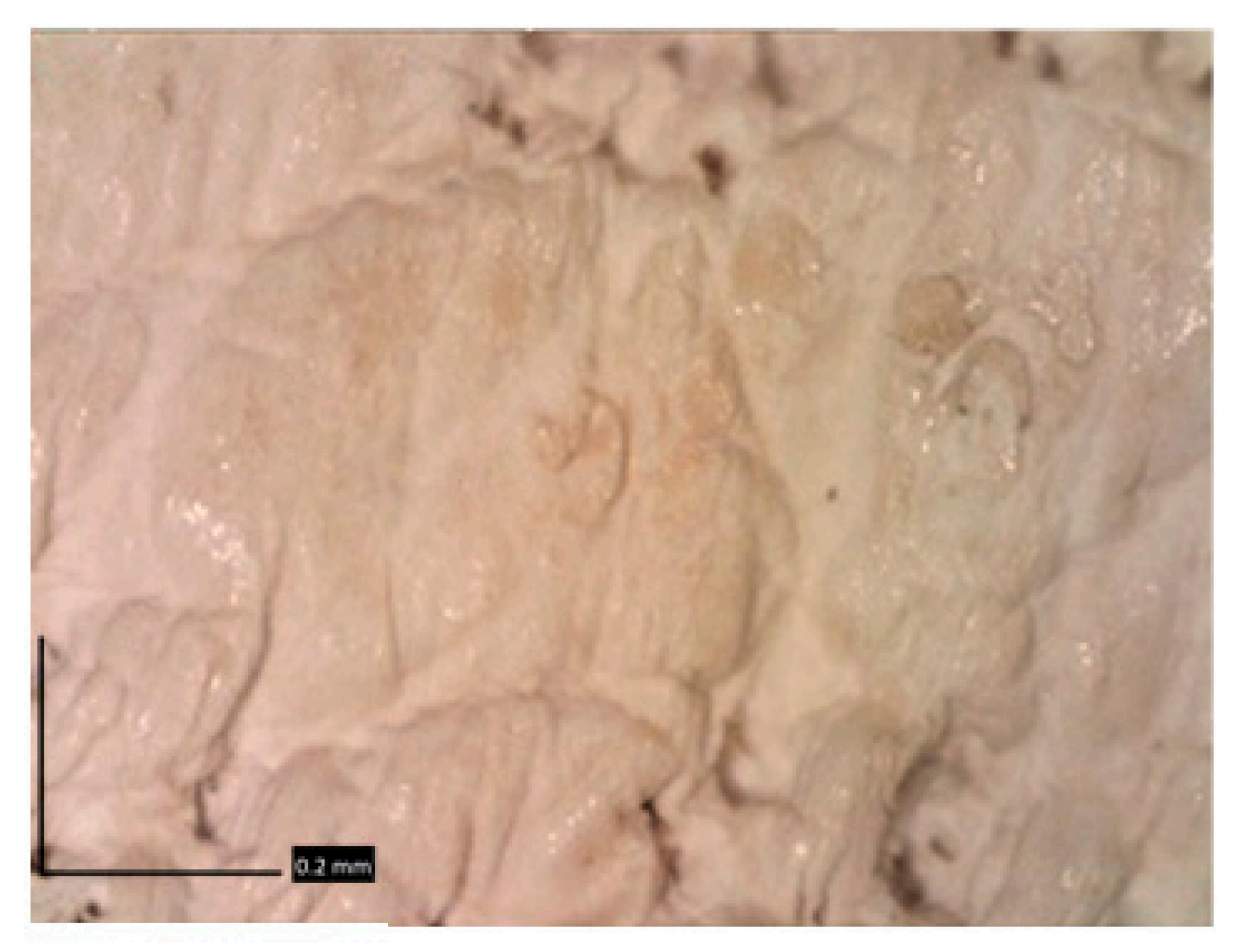	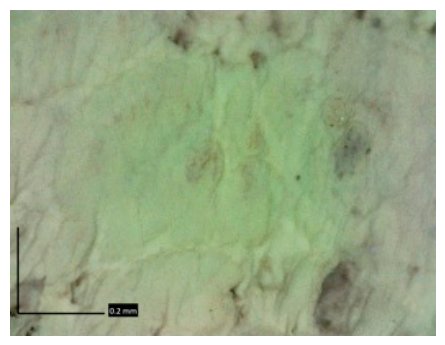
IA_2 g/L	NT	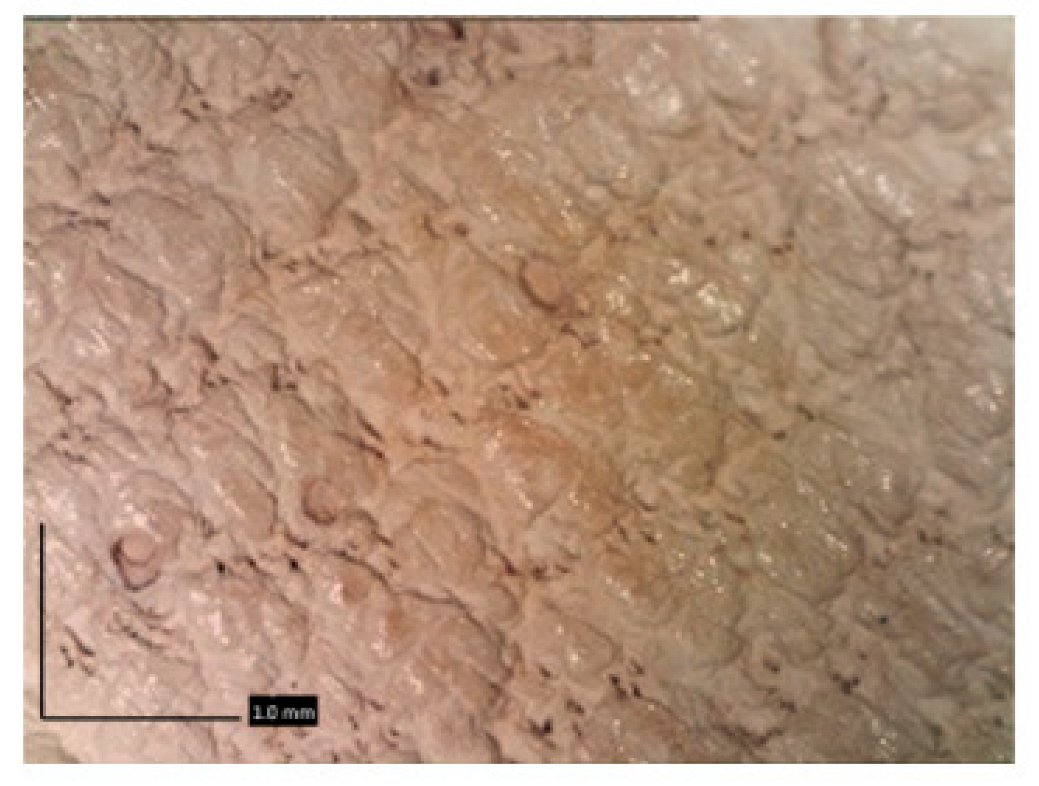	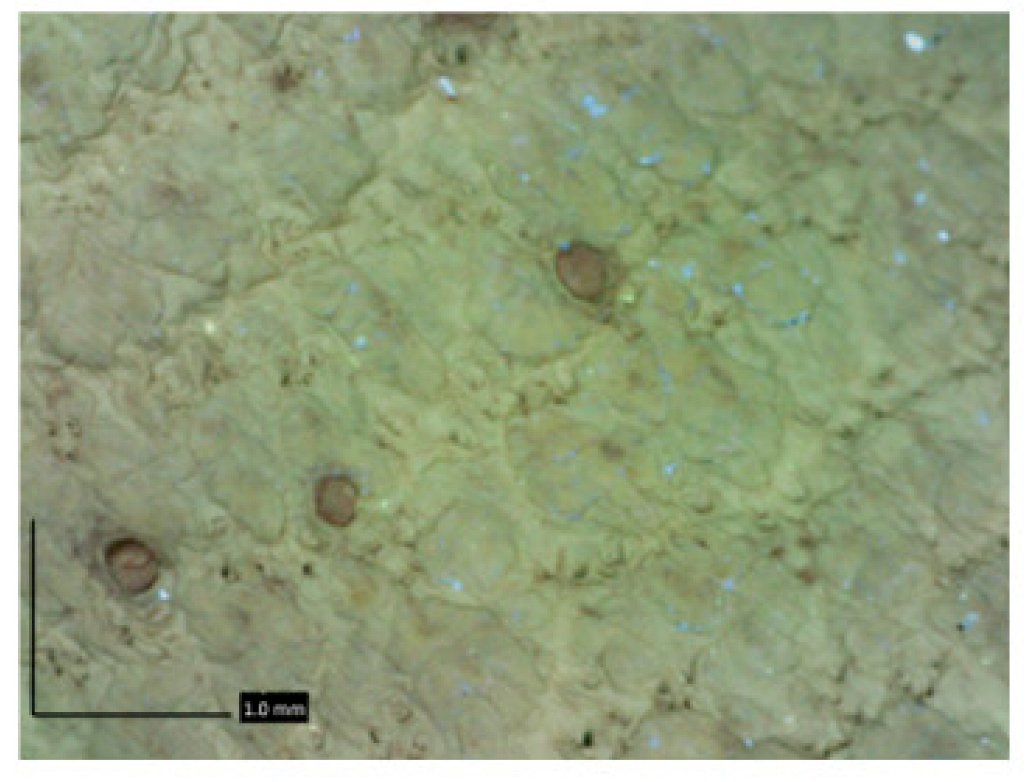	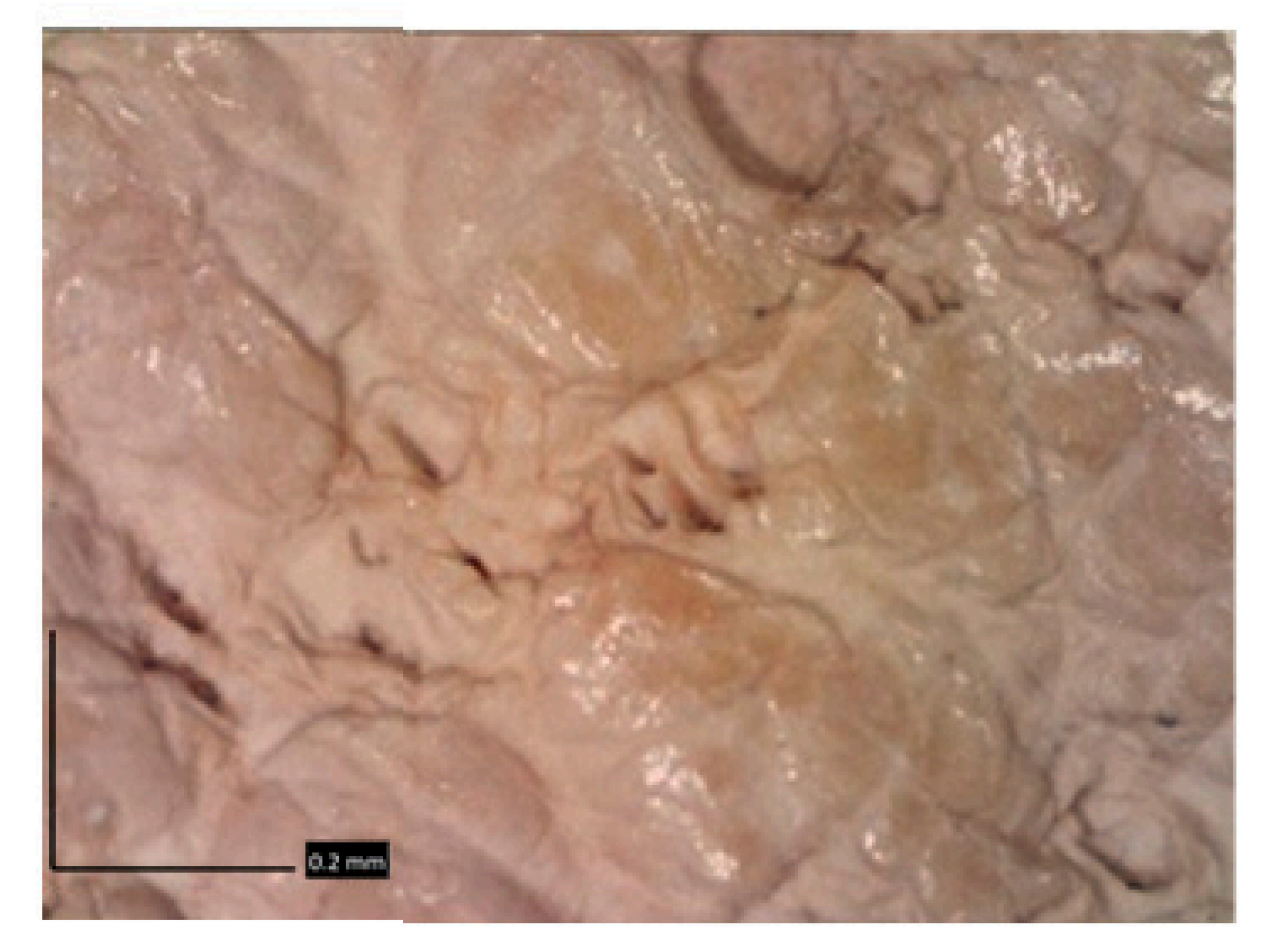	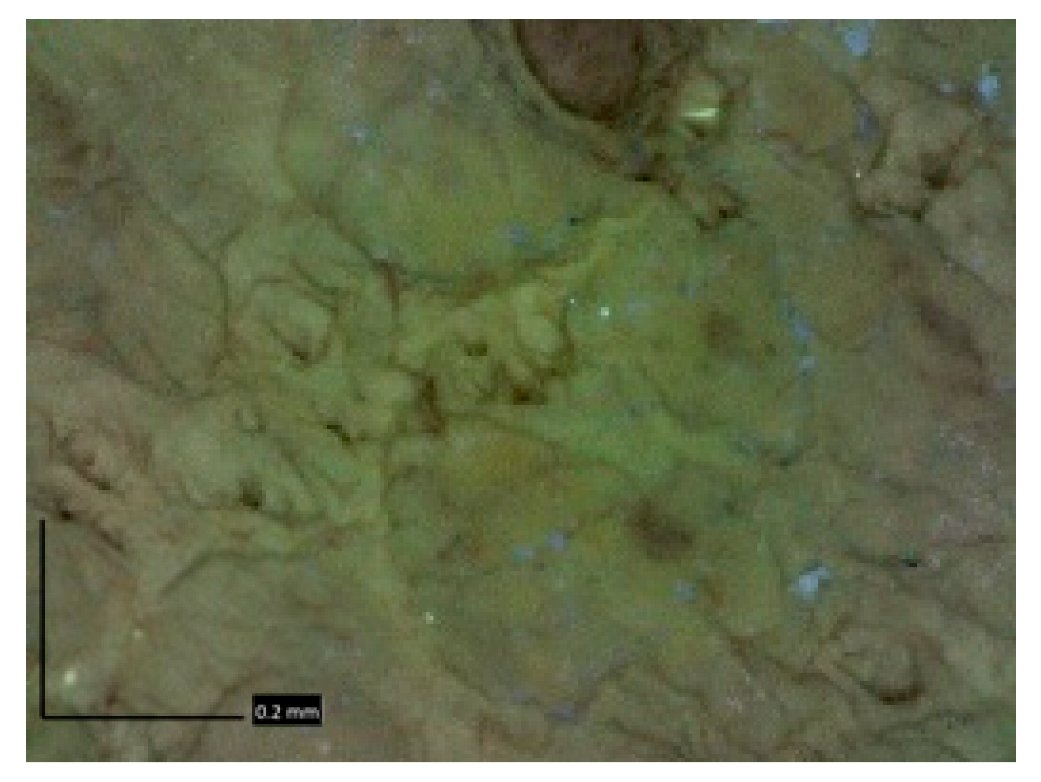
IA_2 g/L	Nanocollagen	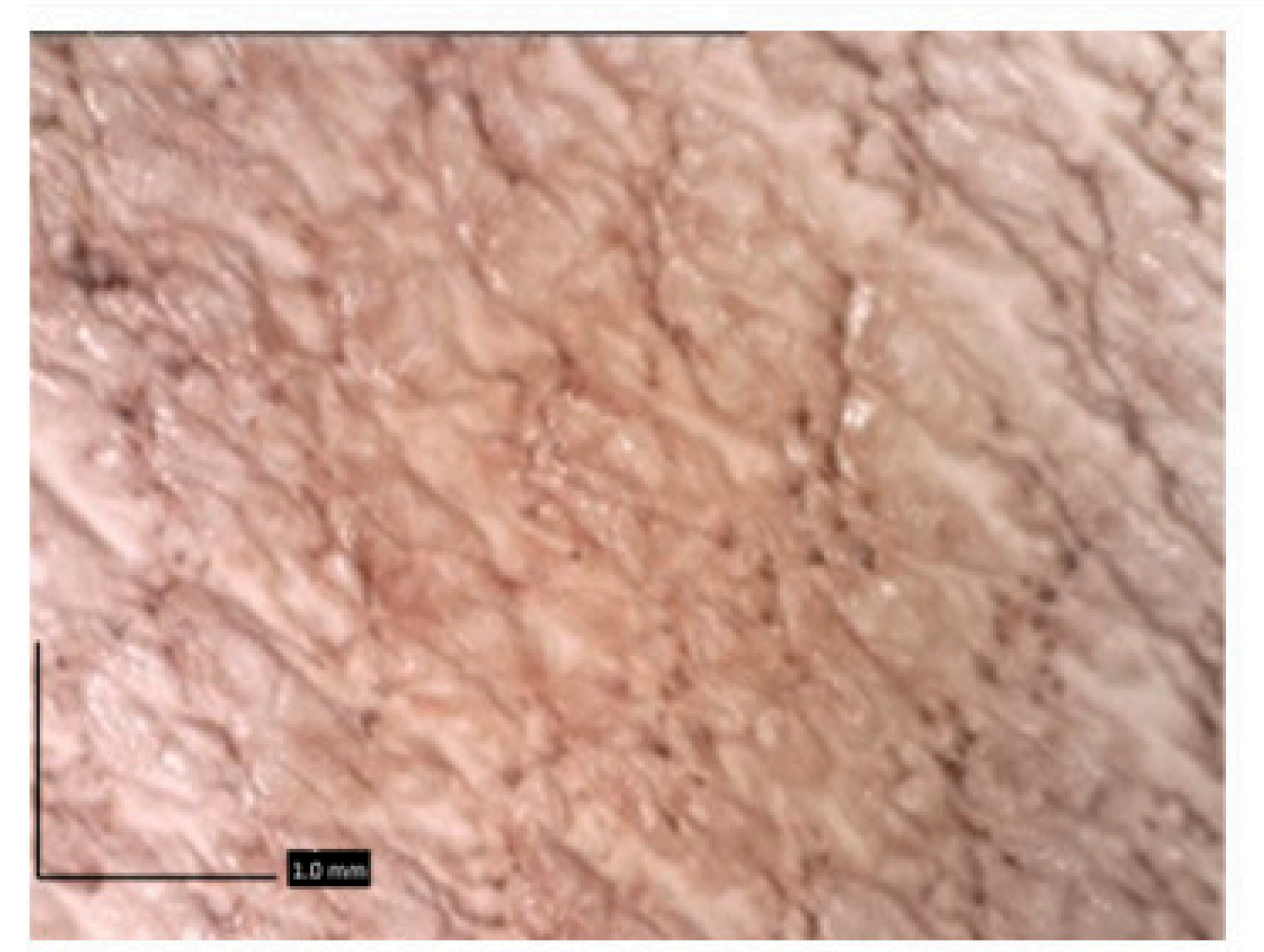	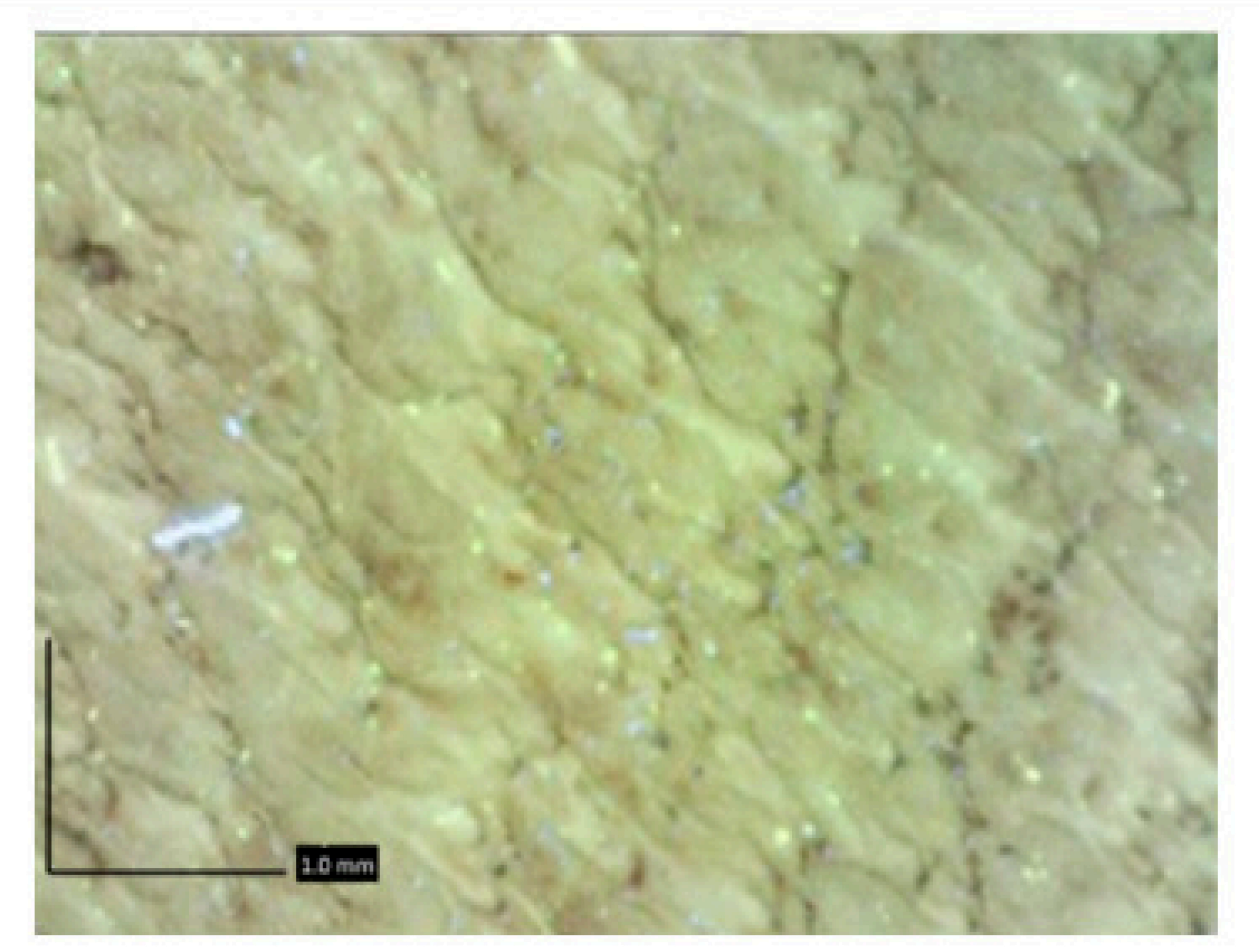	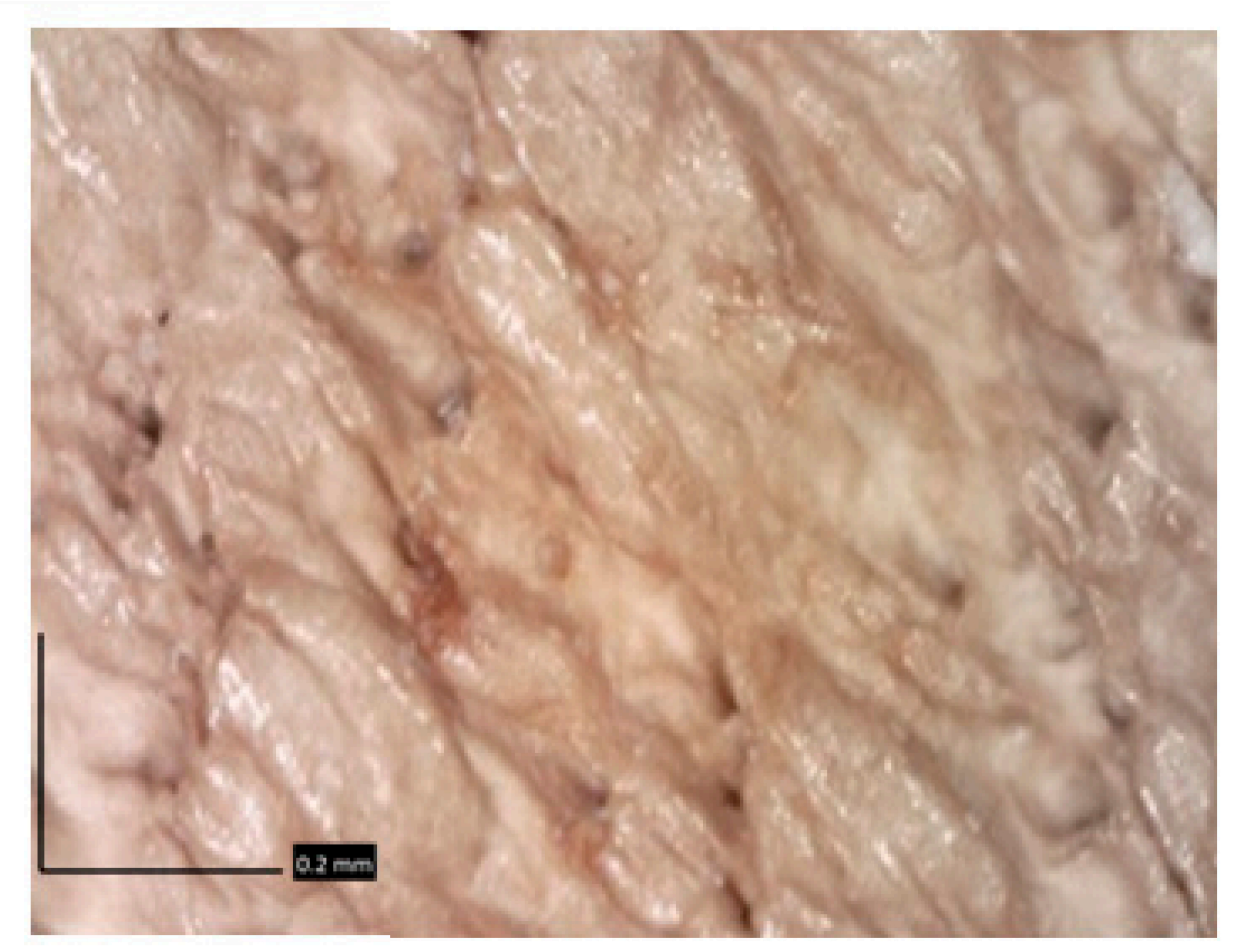	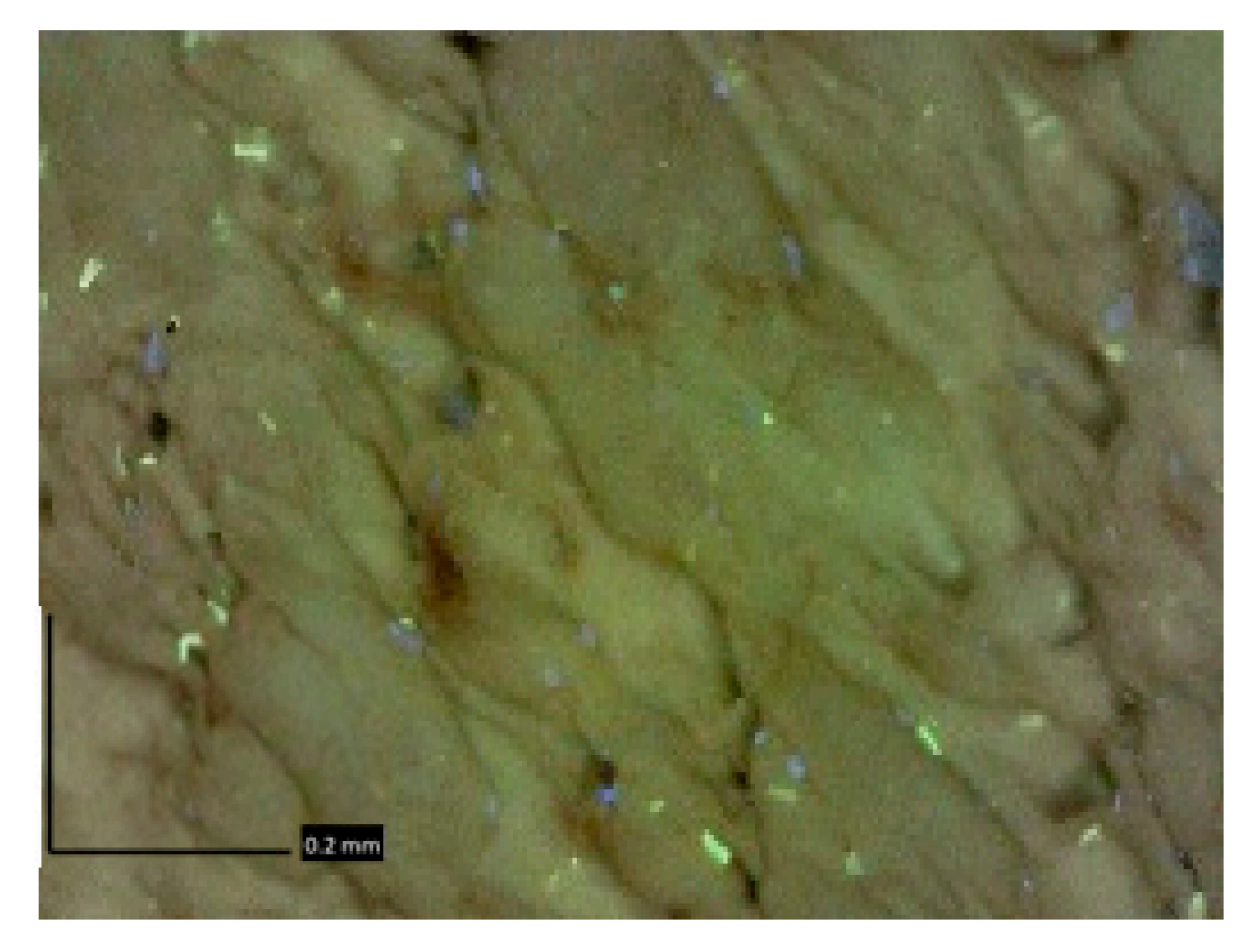
IA_2 g/L	Klucel	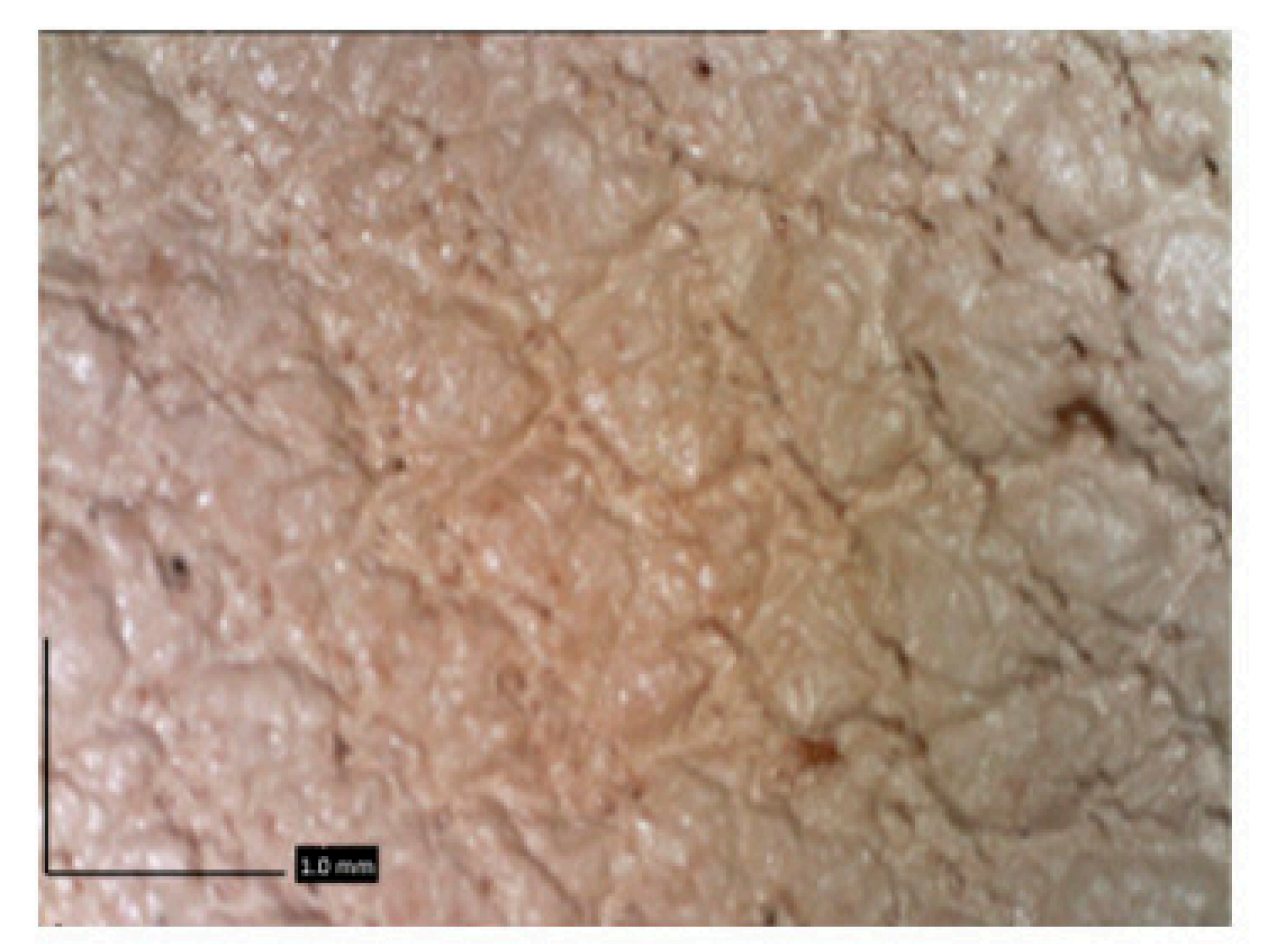	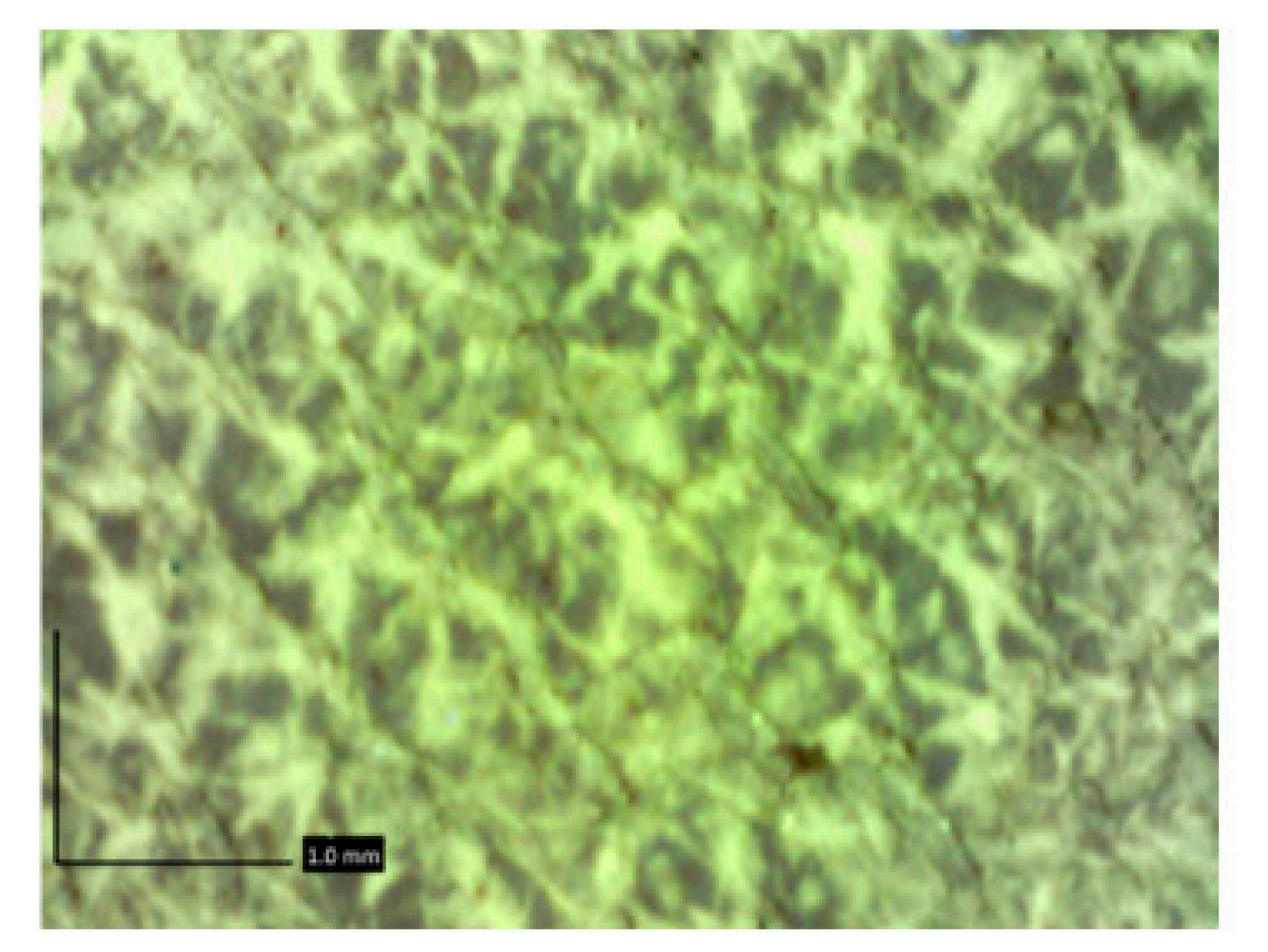	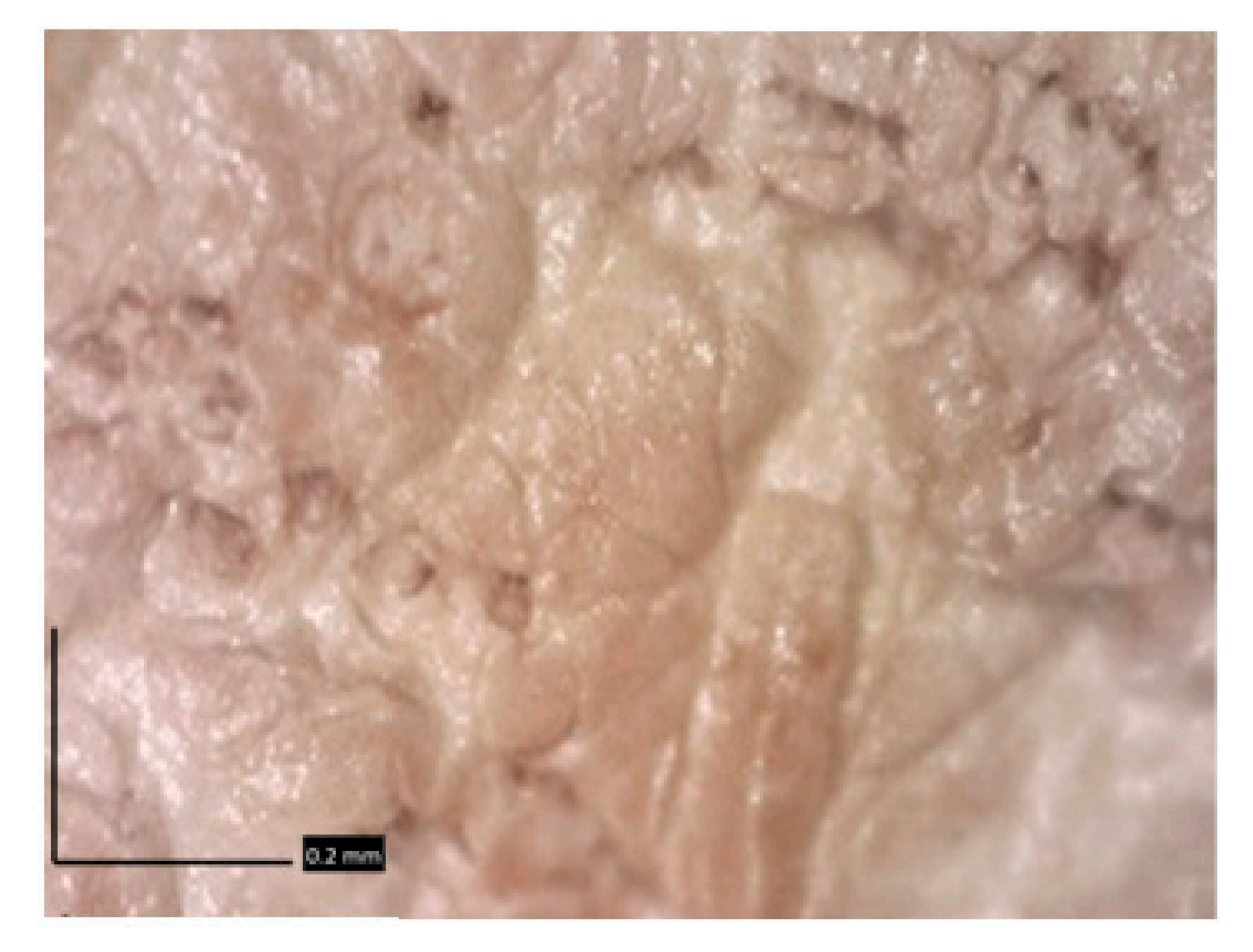	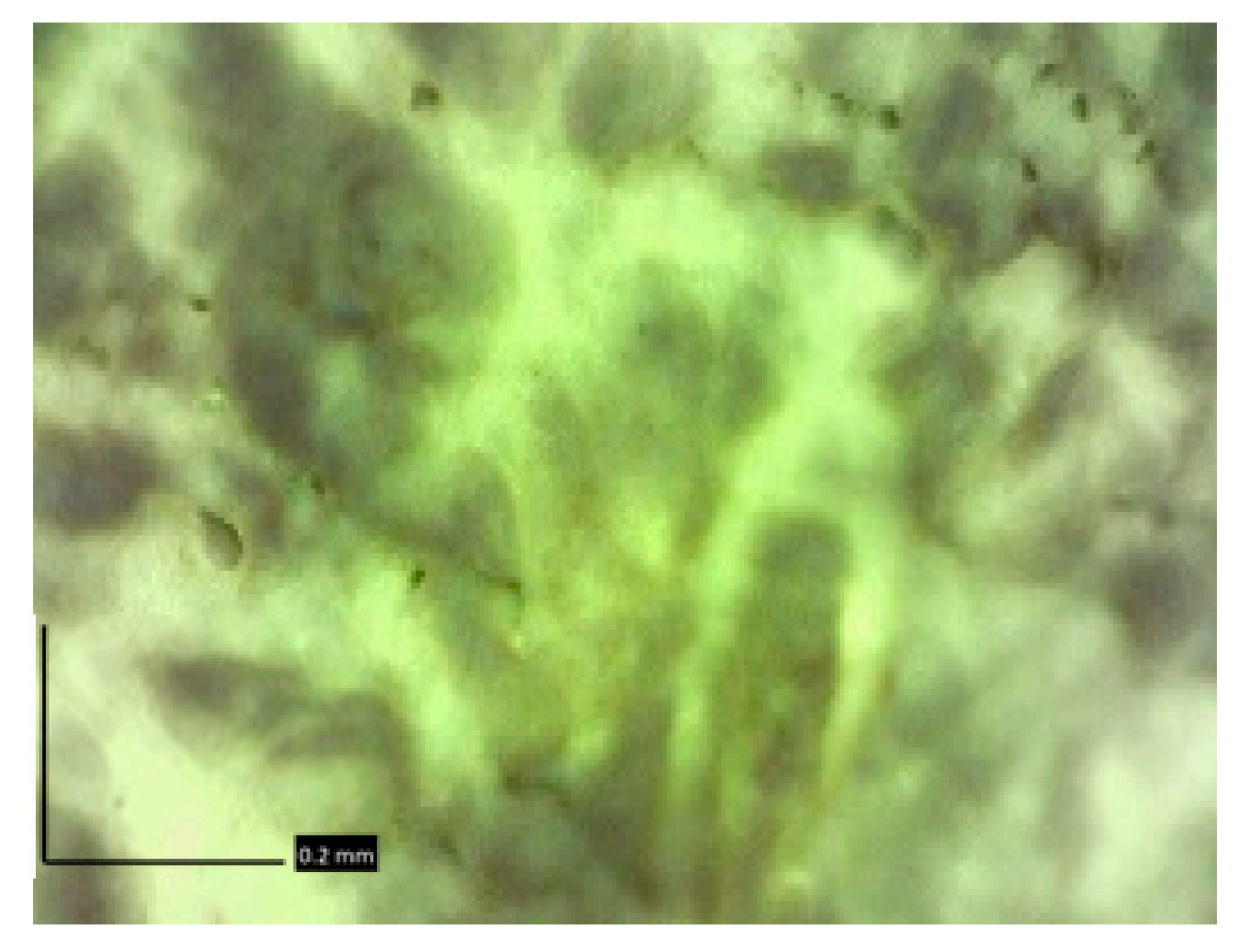
IA_10 g/L	NT	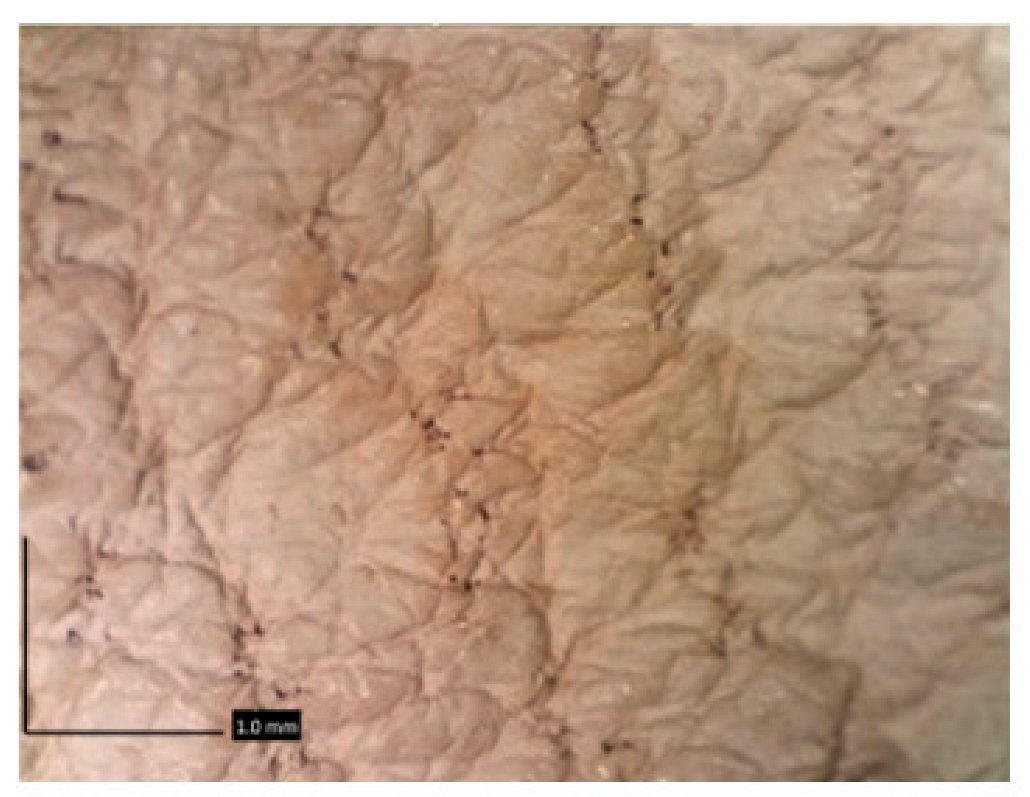	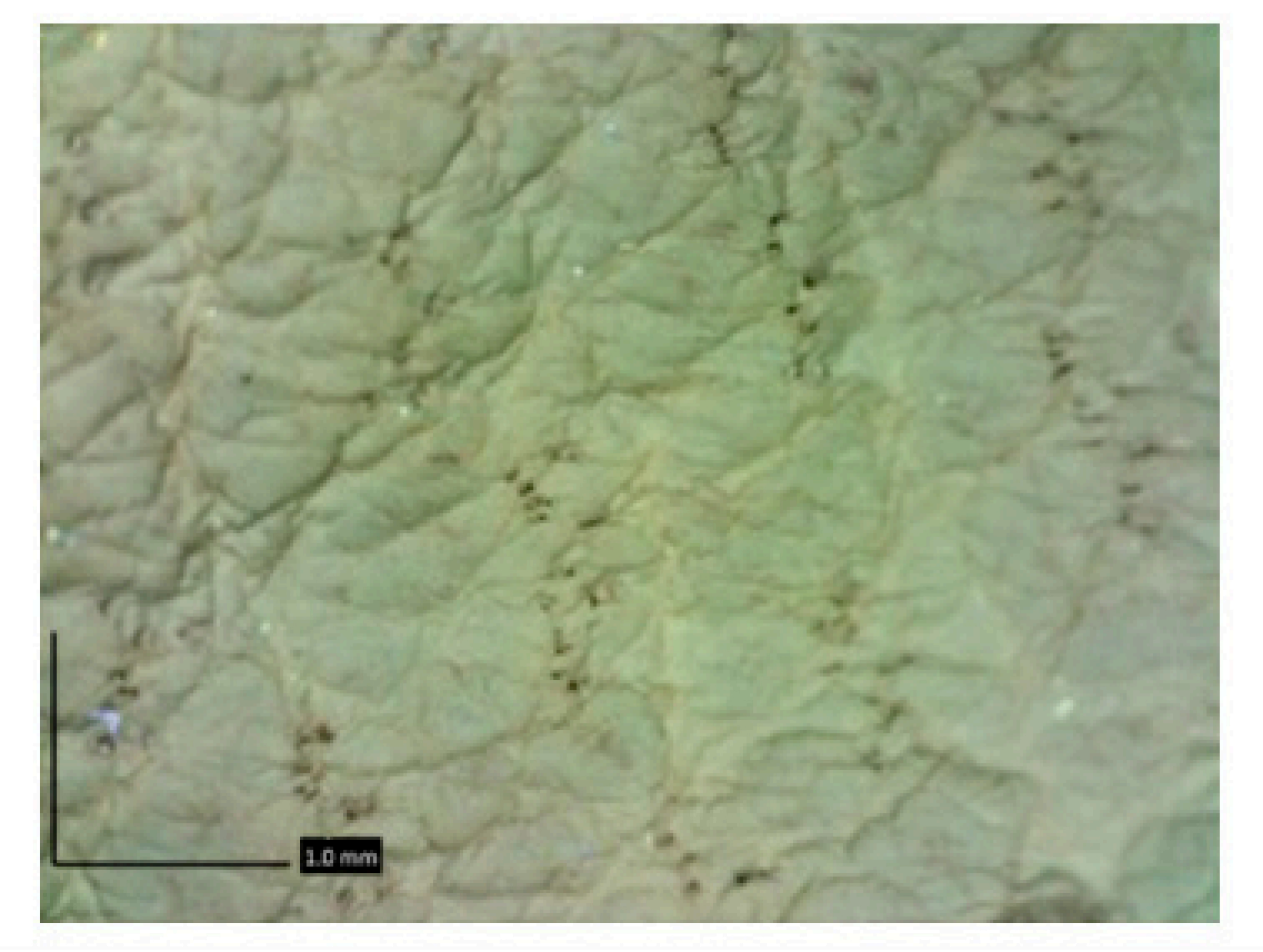	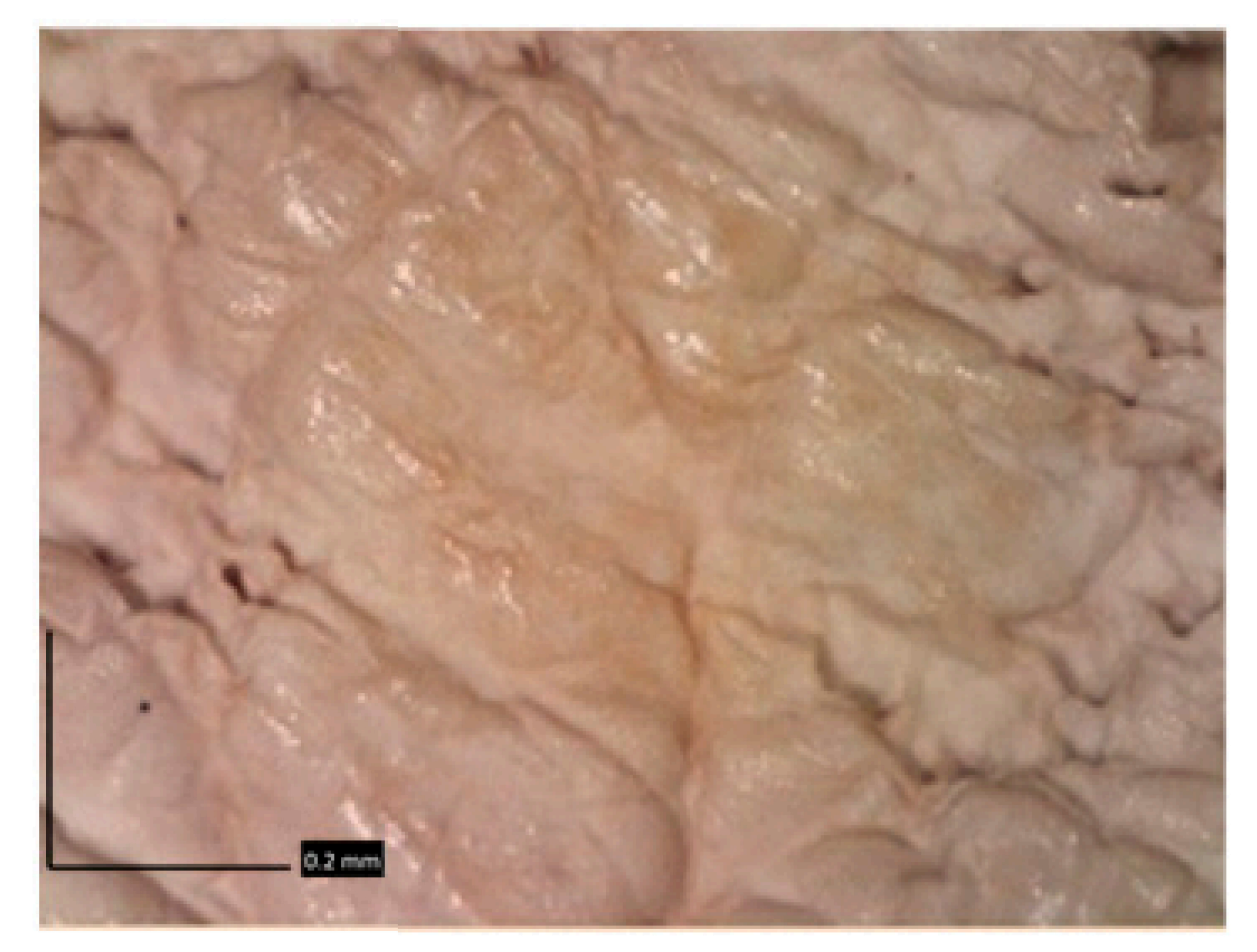	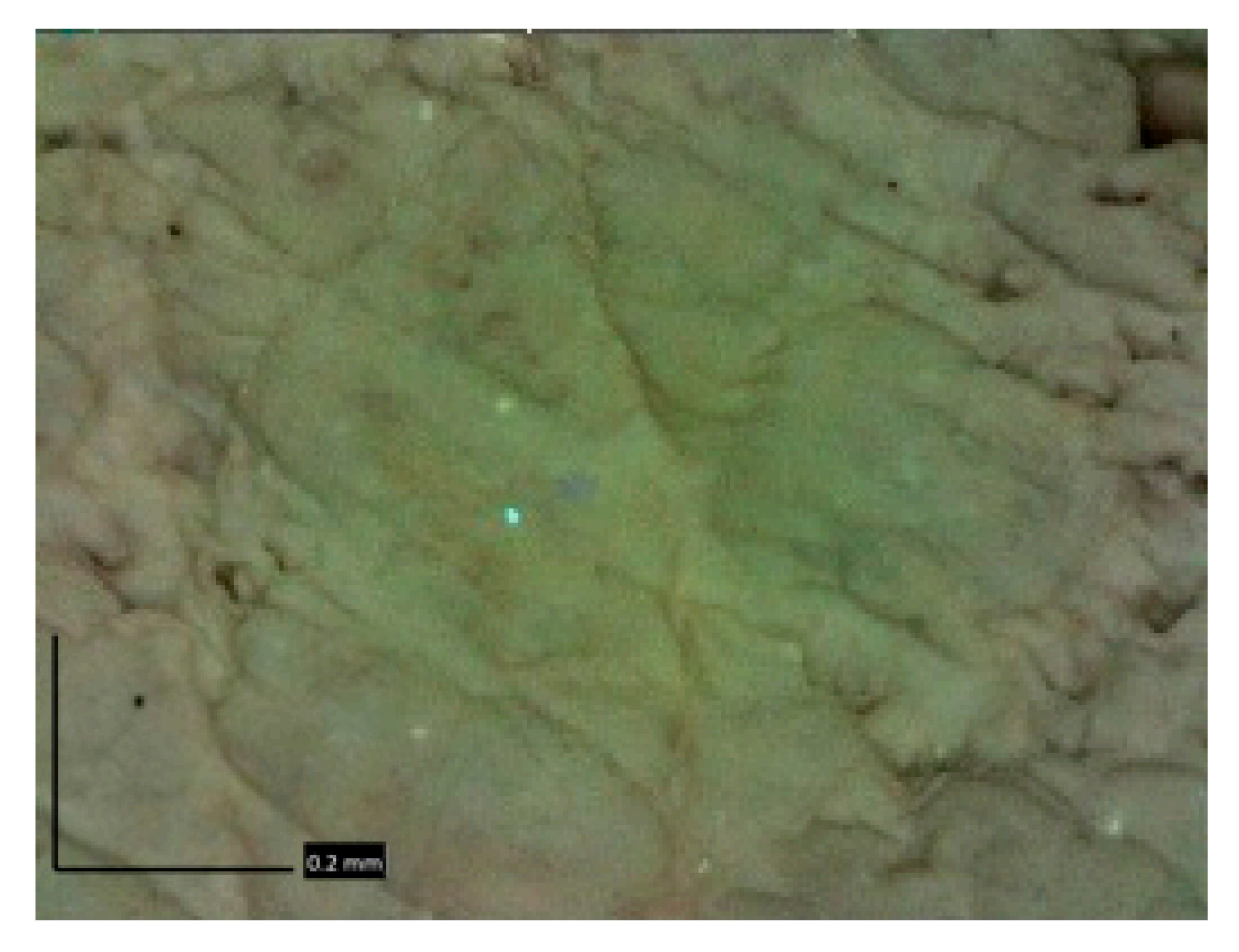
IA_10 g/L	Nanocollagen	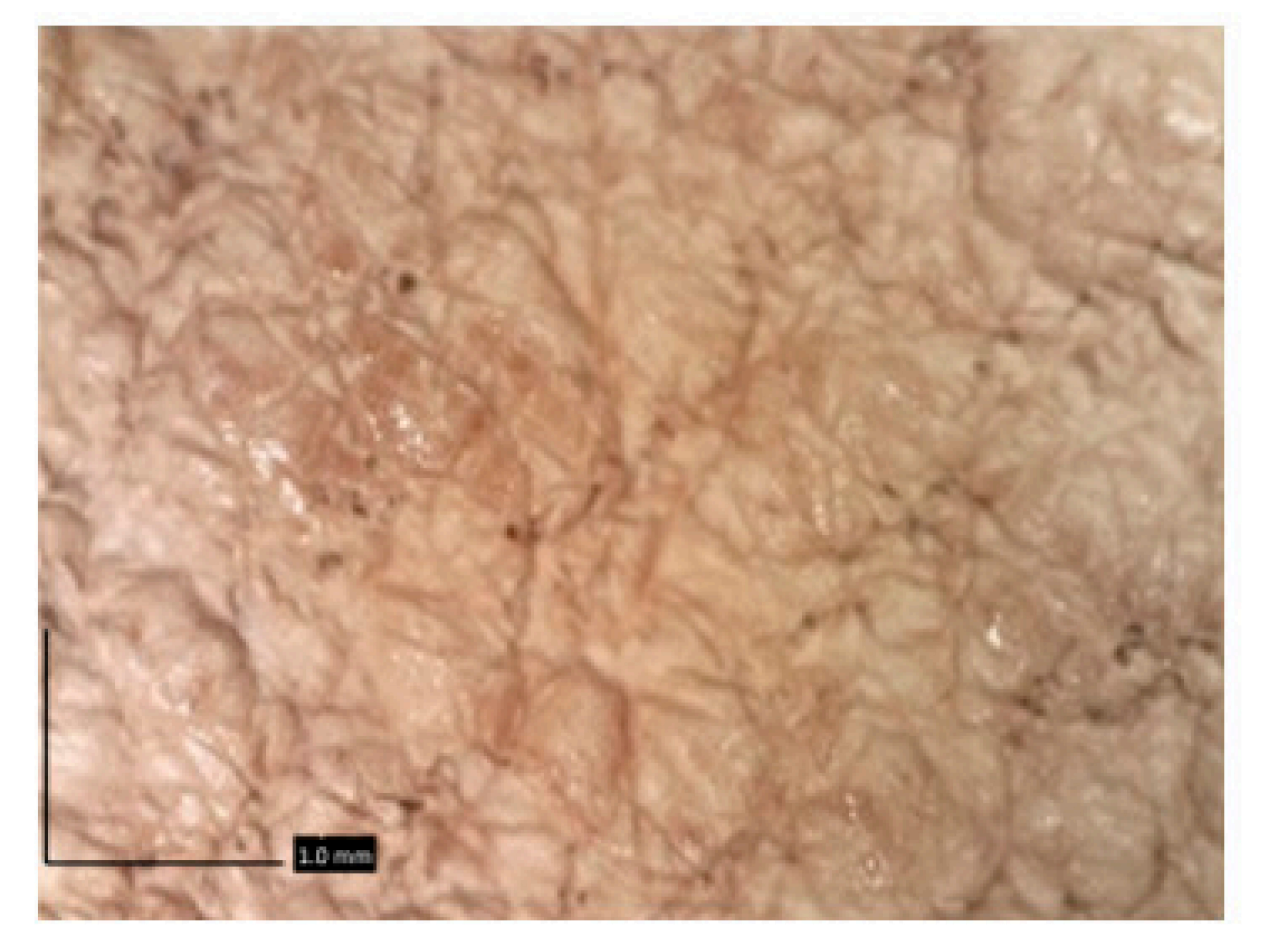	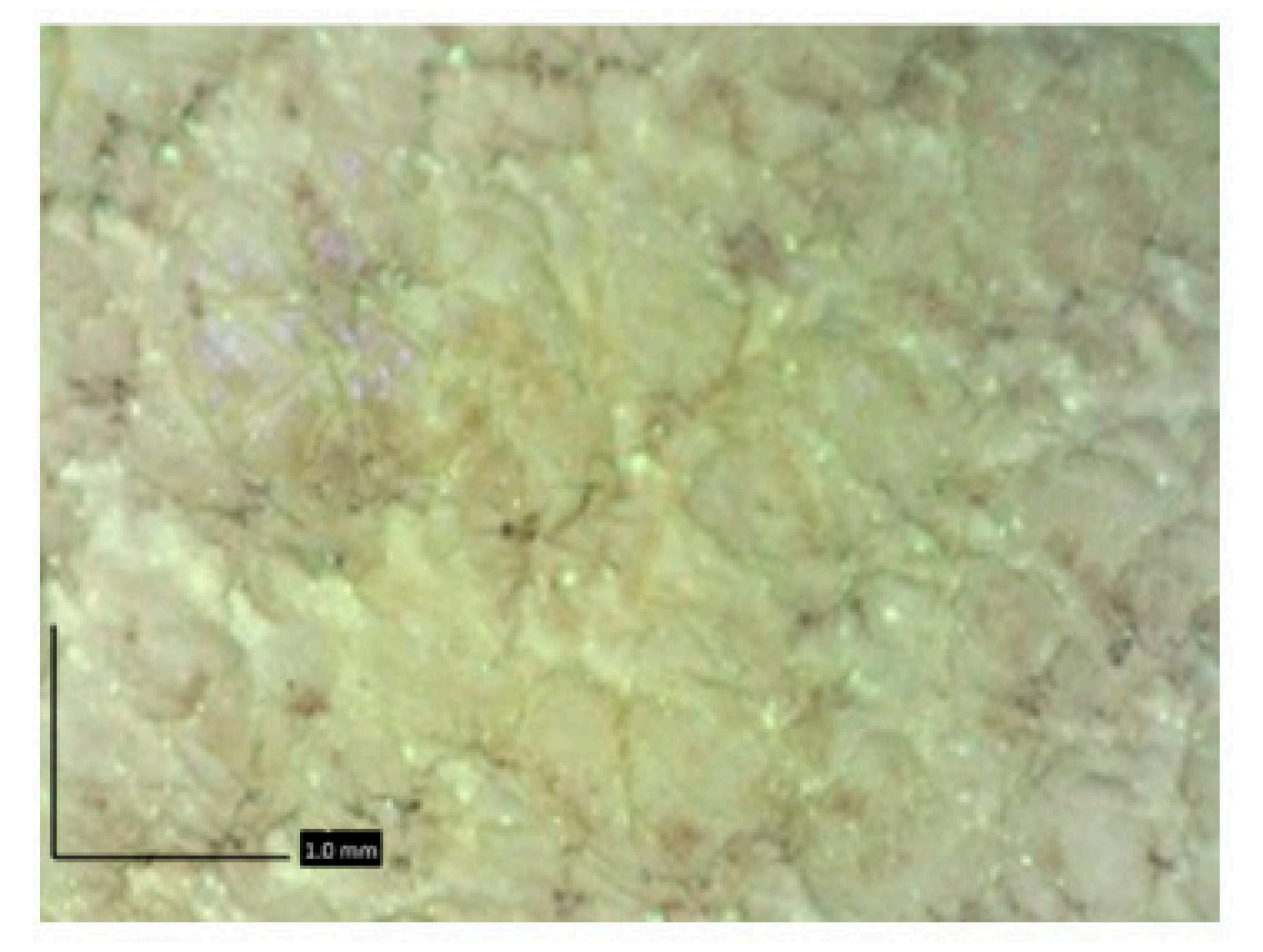	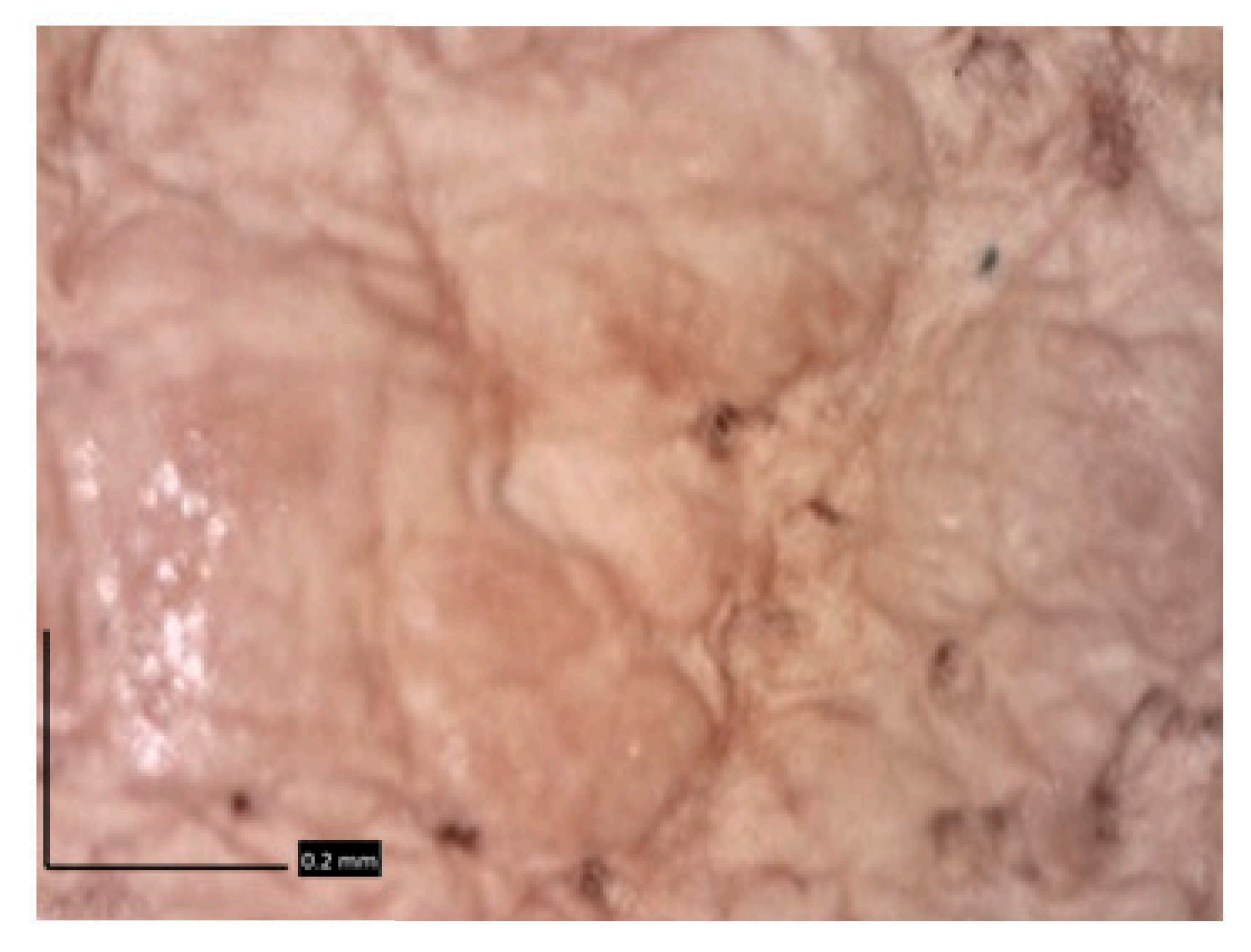	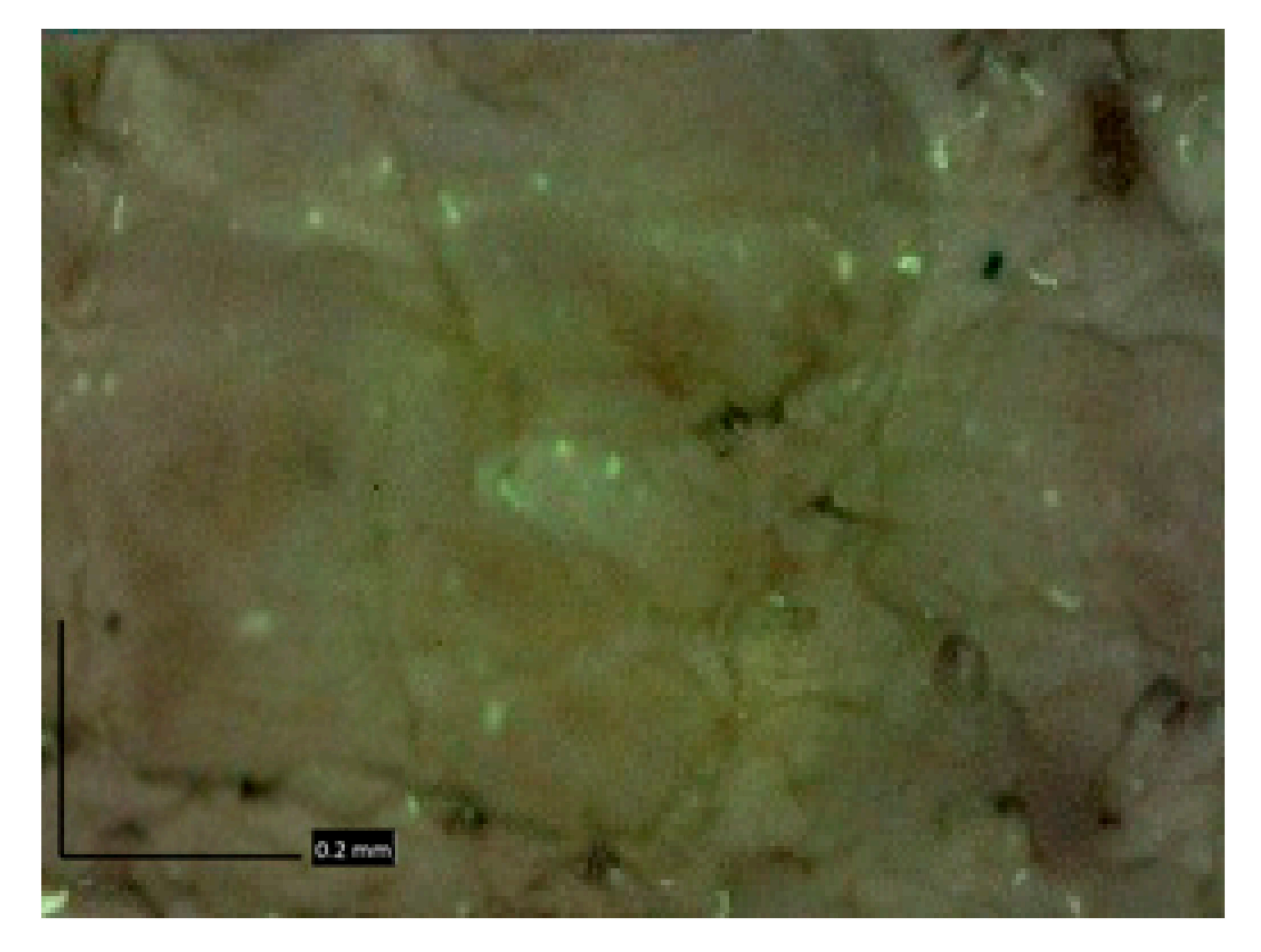
IA_10 g/L	Klucel	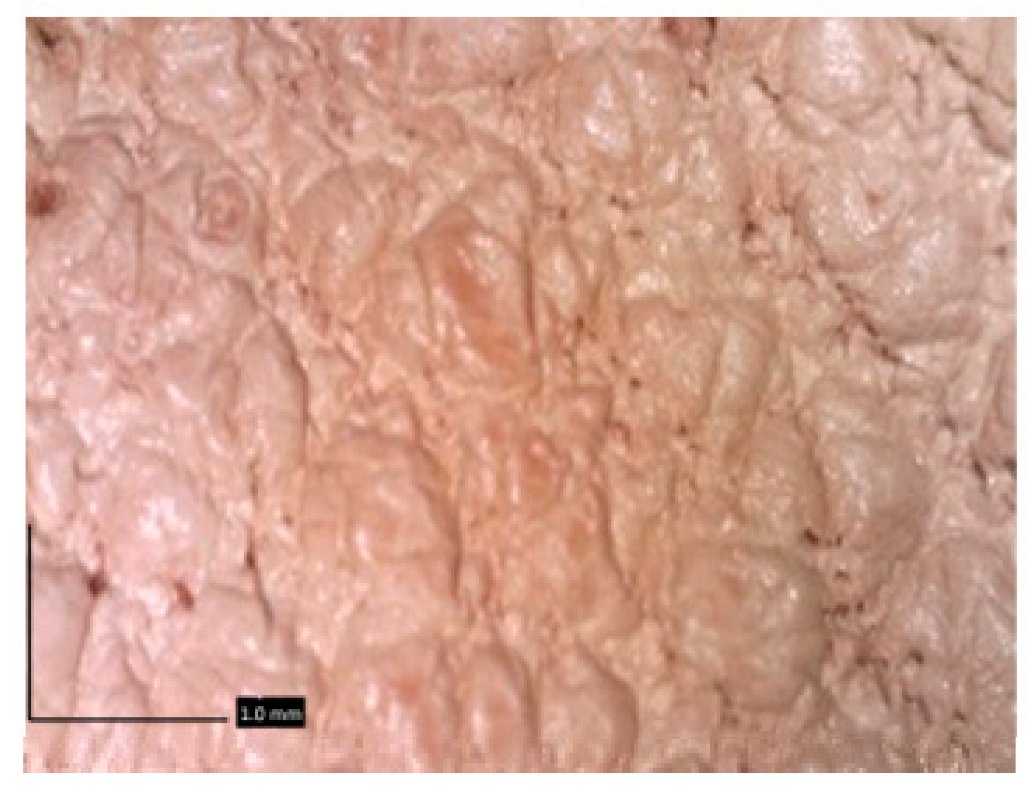	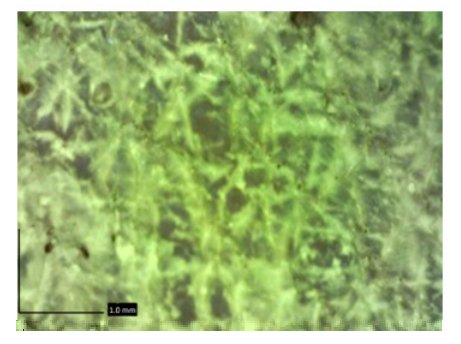	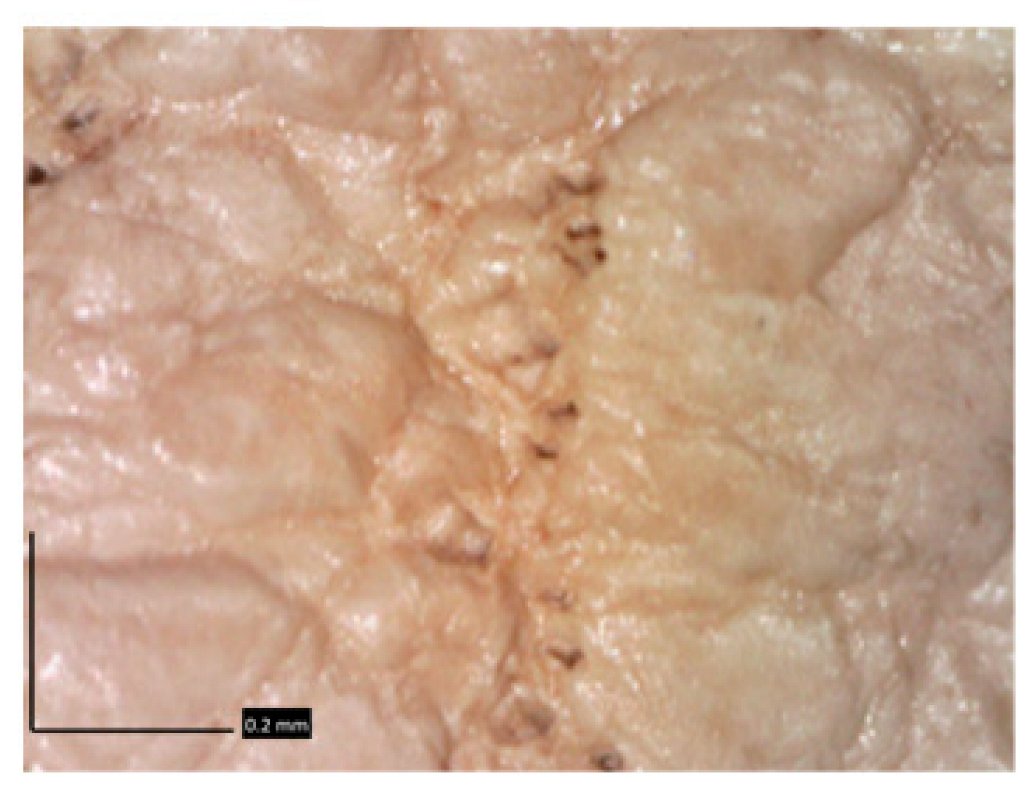	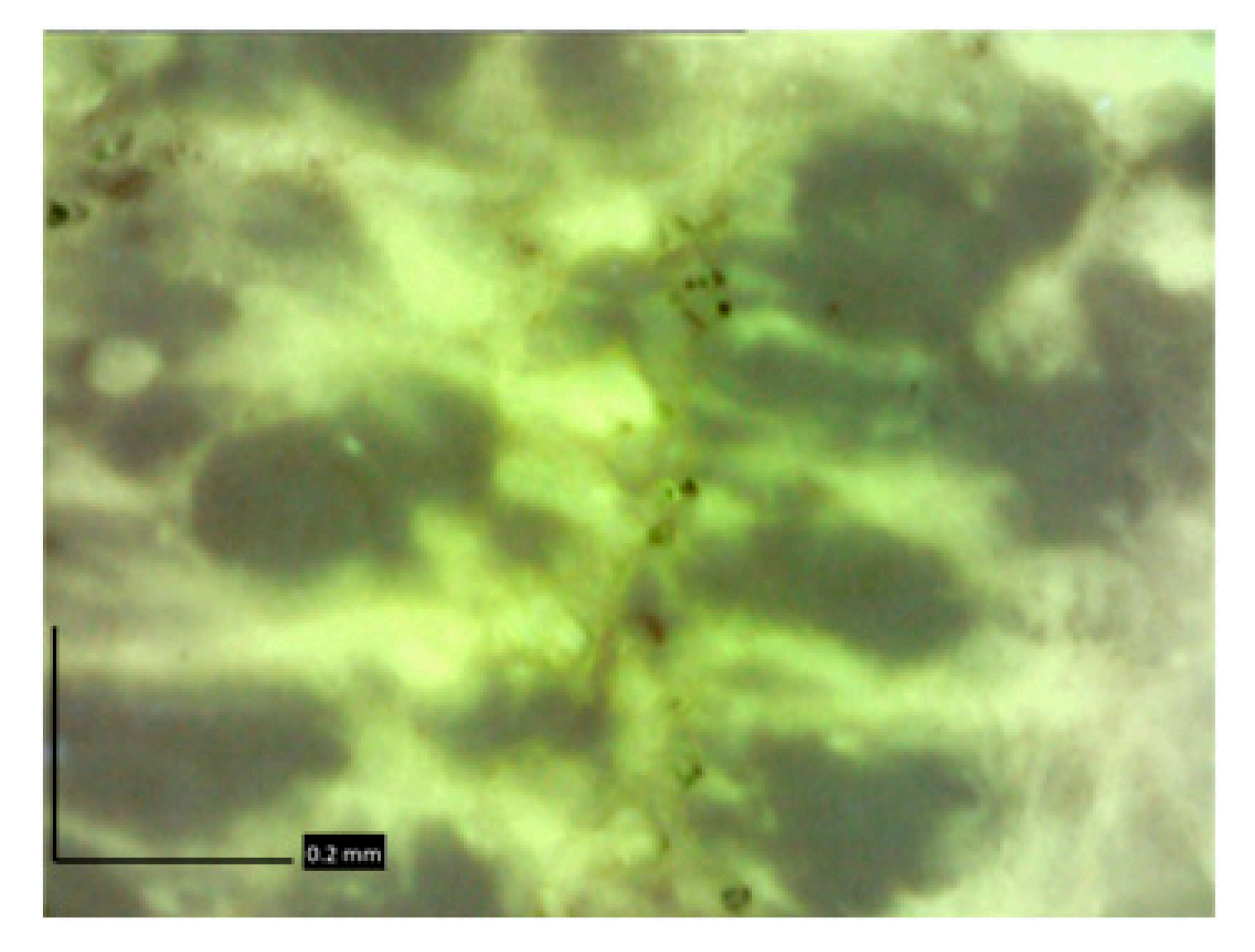
INA_2 g/L	NT	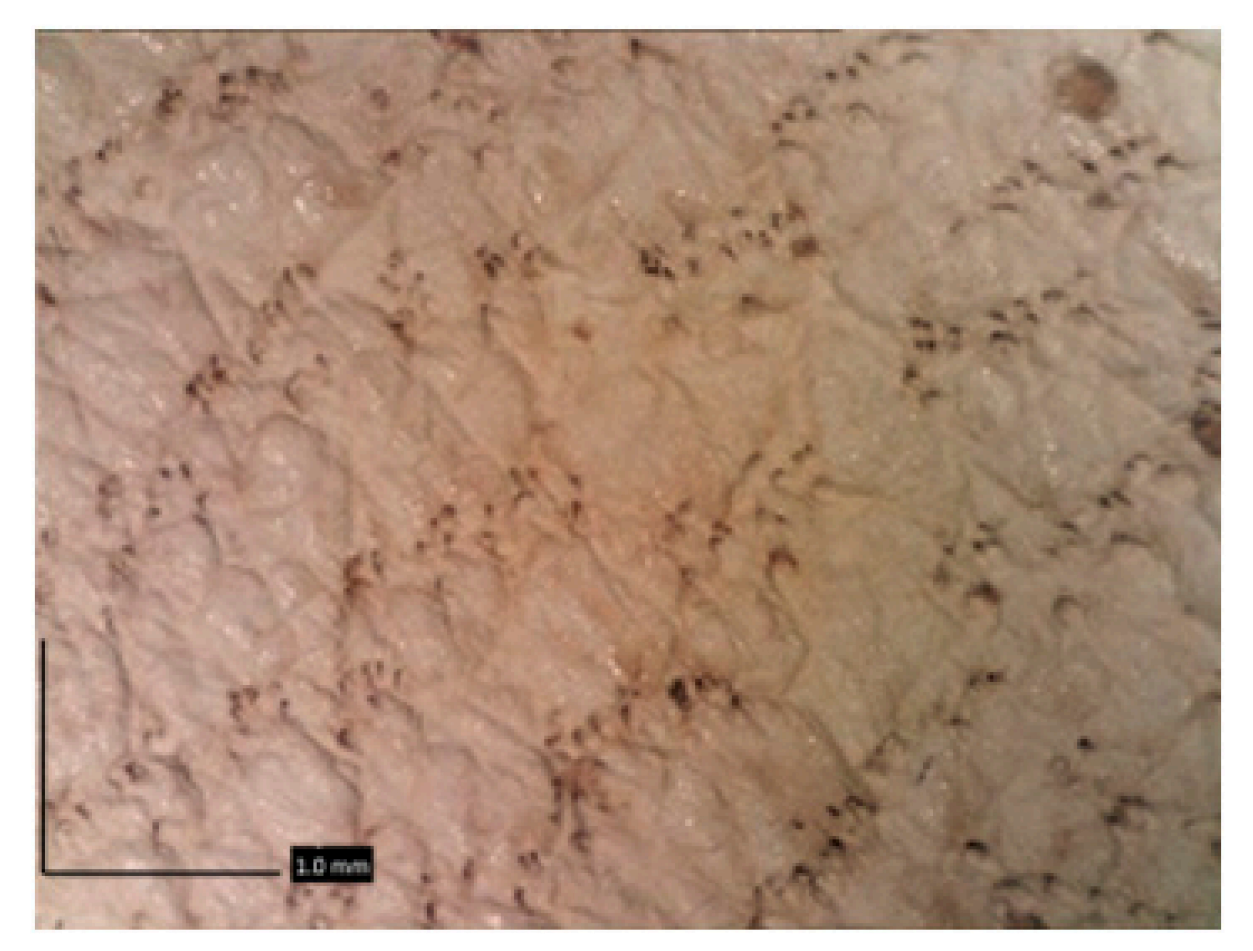	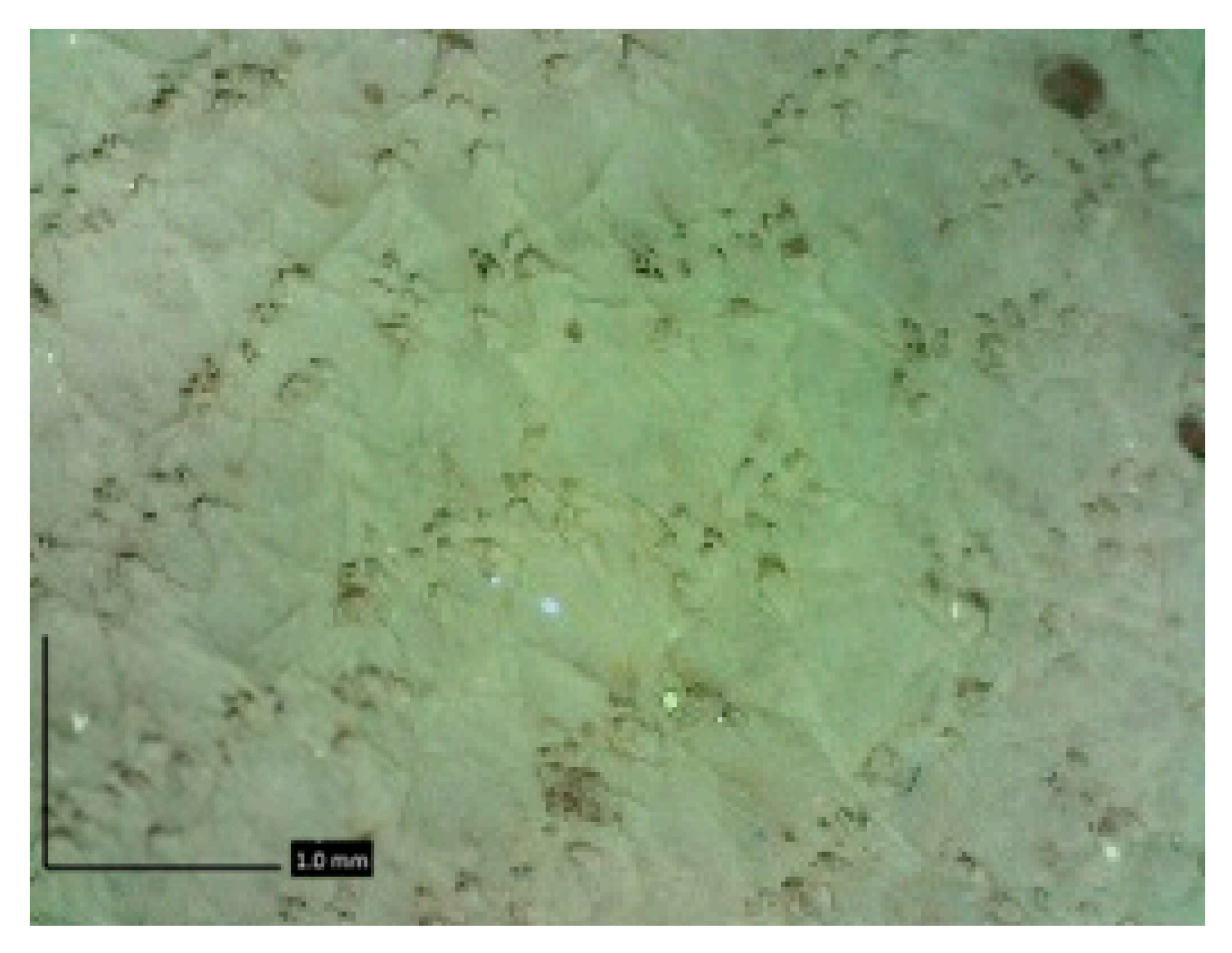	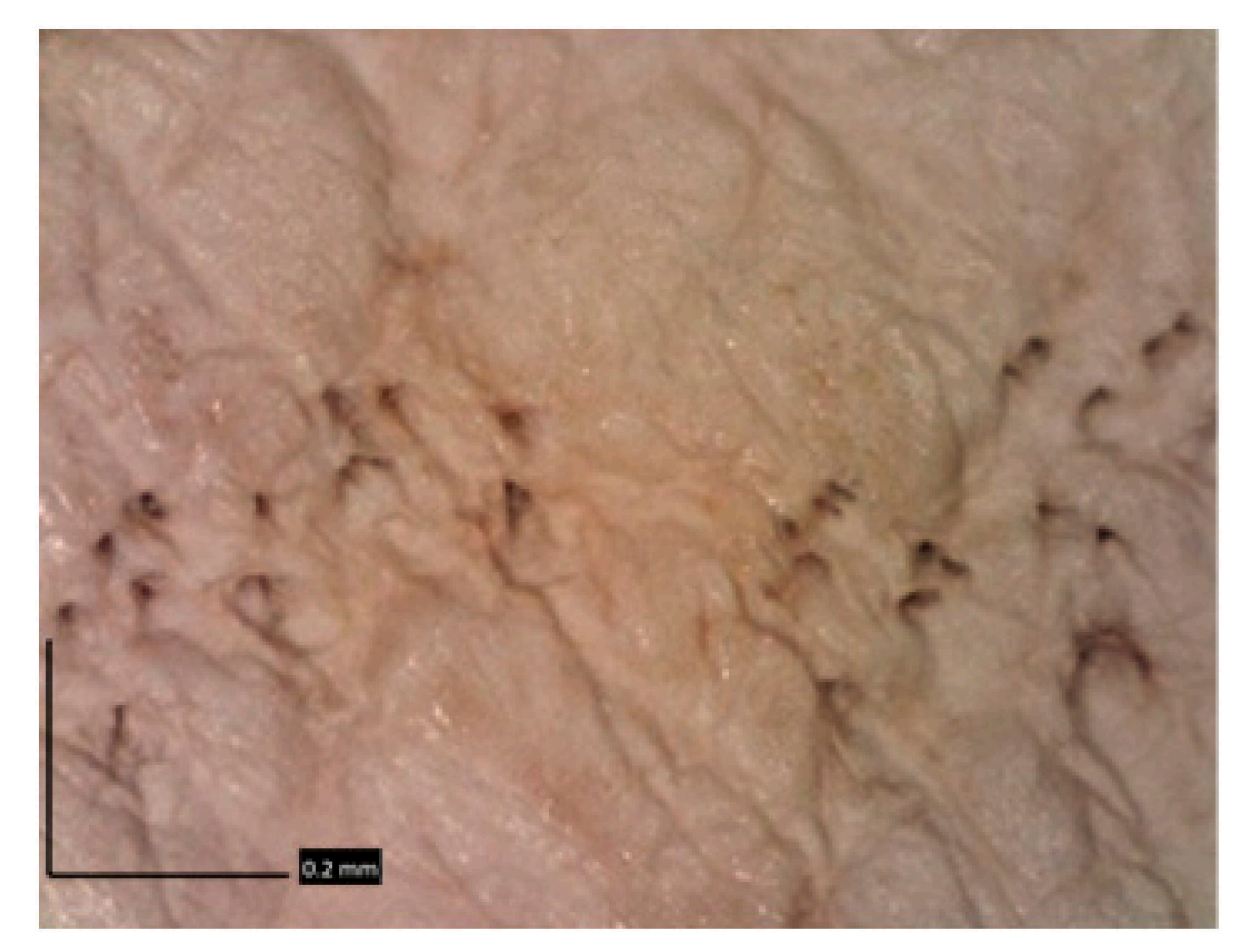	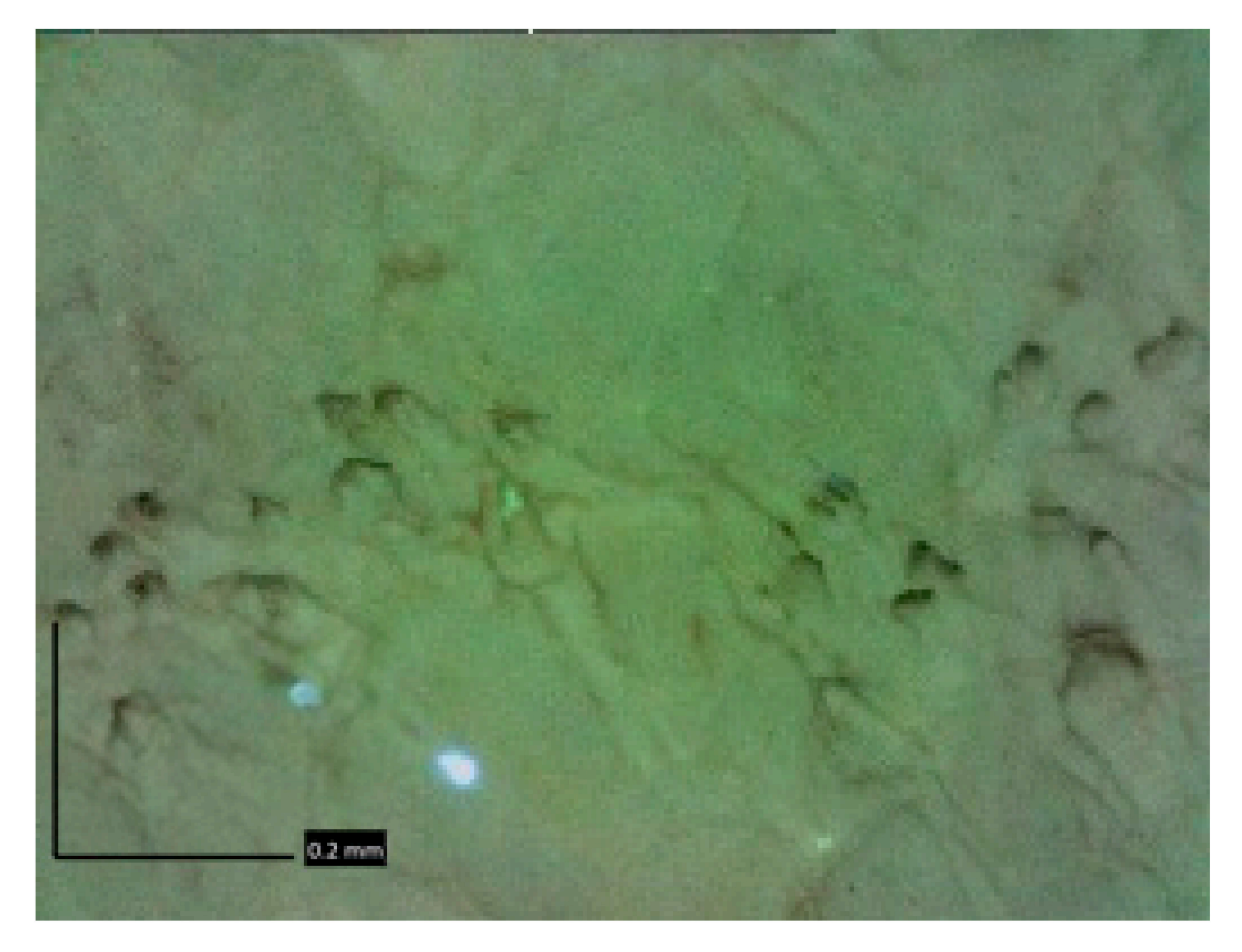
INA_2 g/L	Nanocollagen	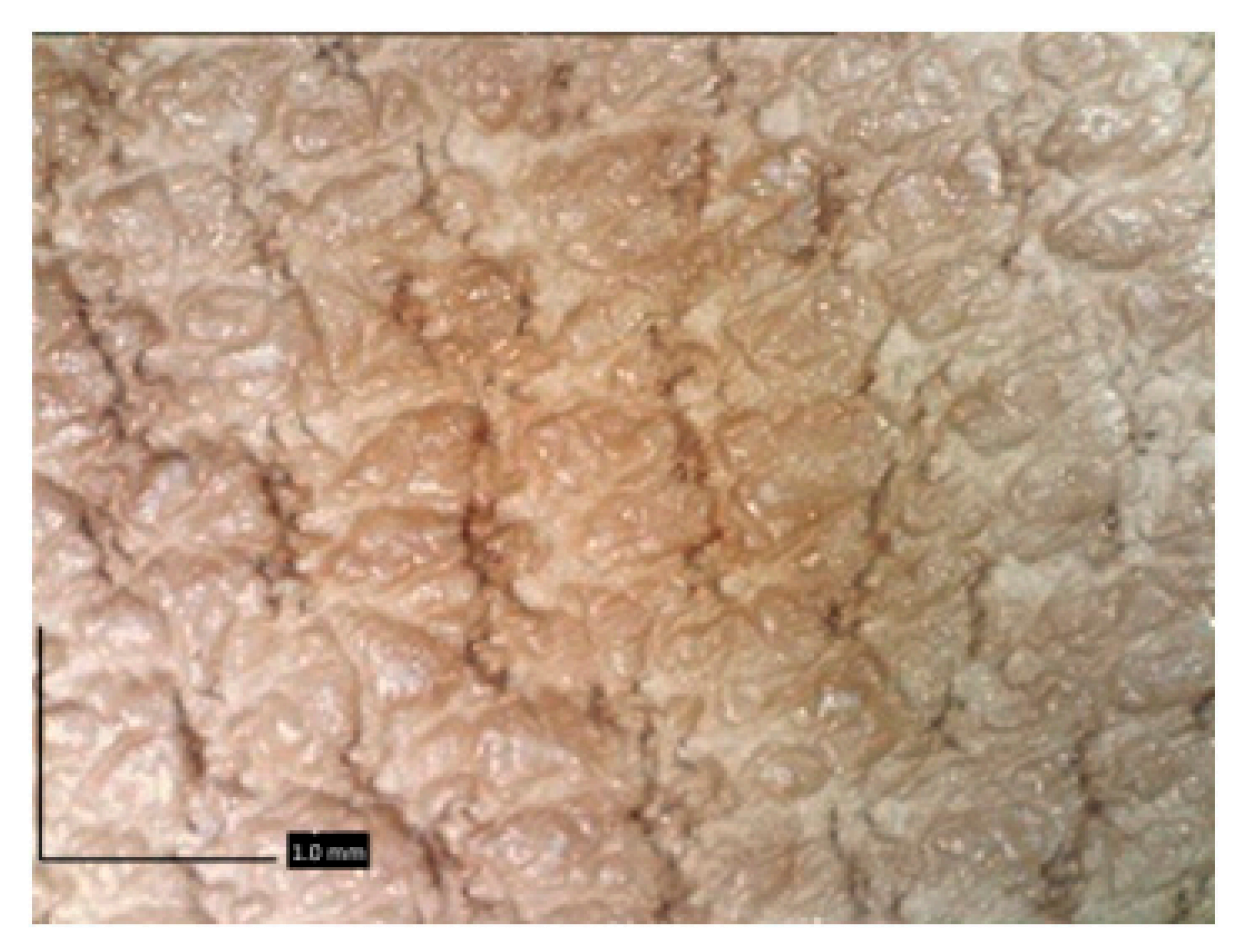	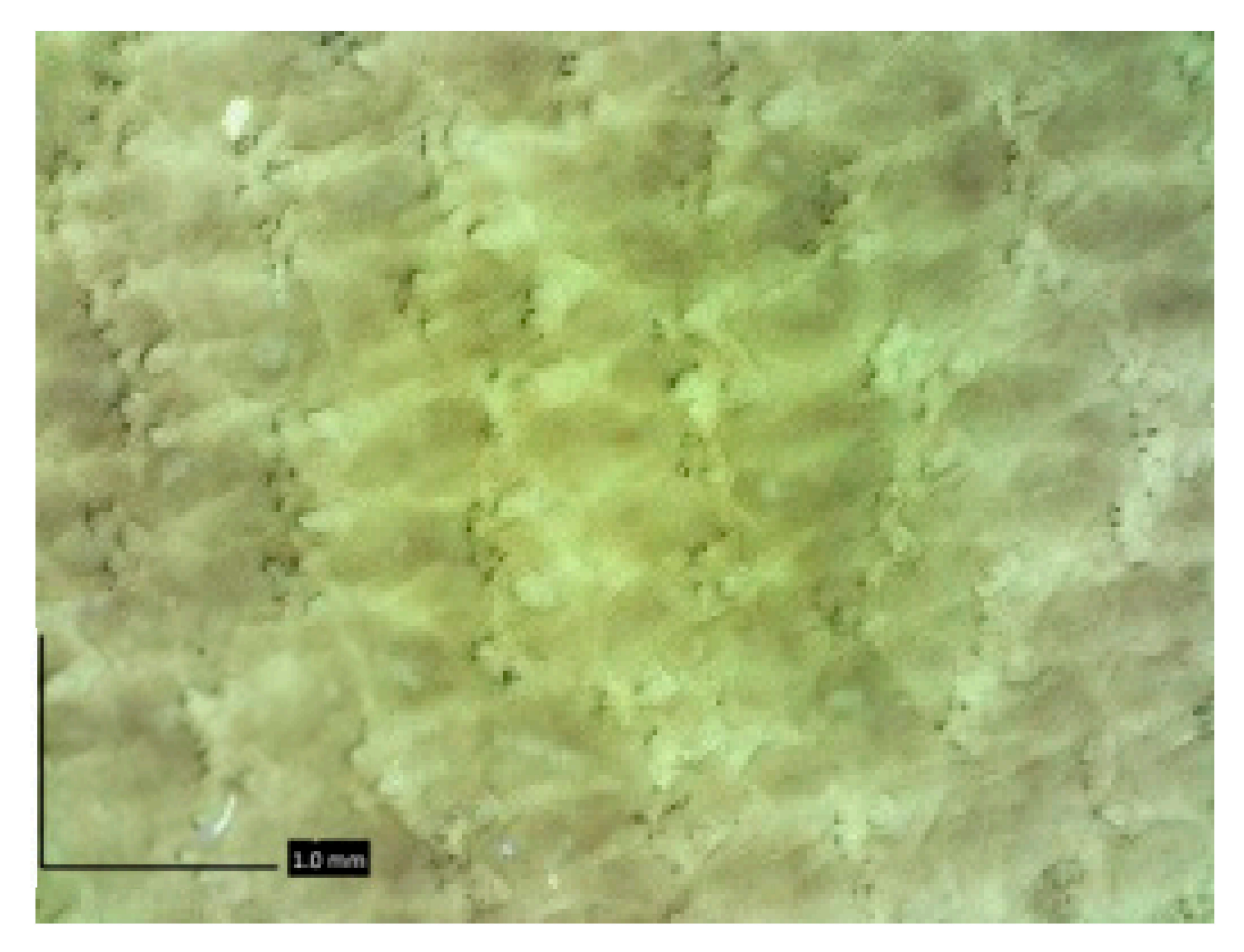	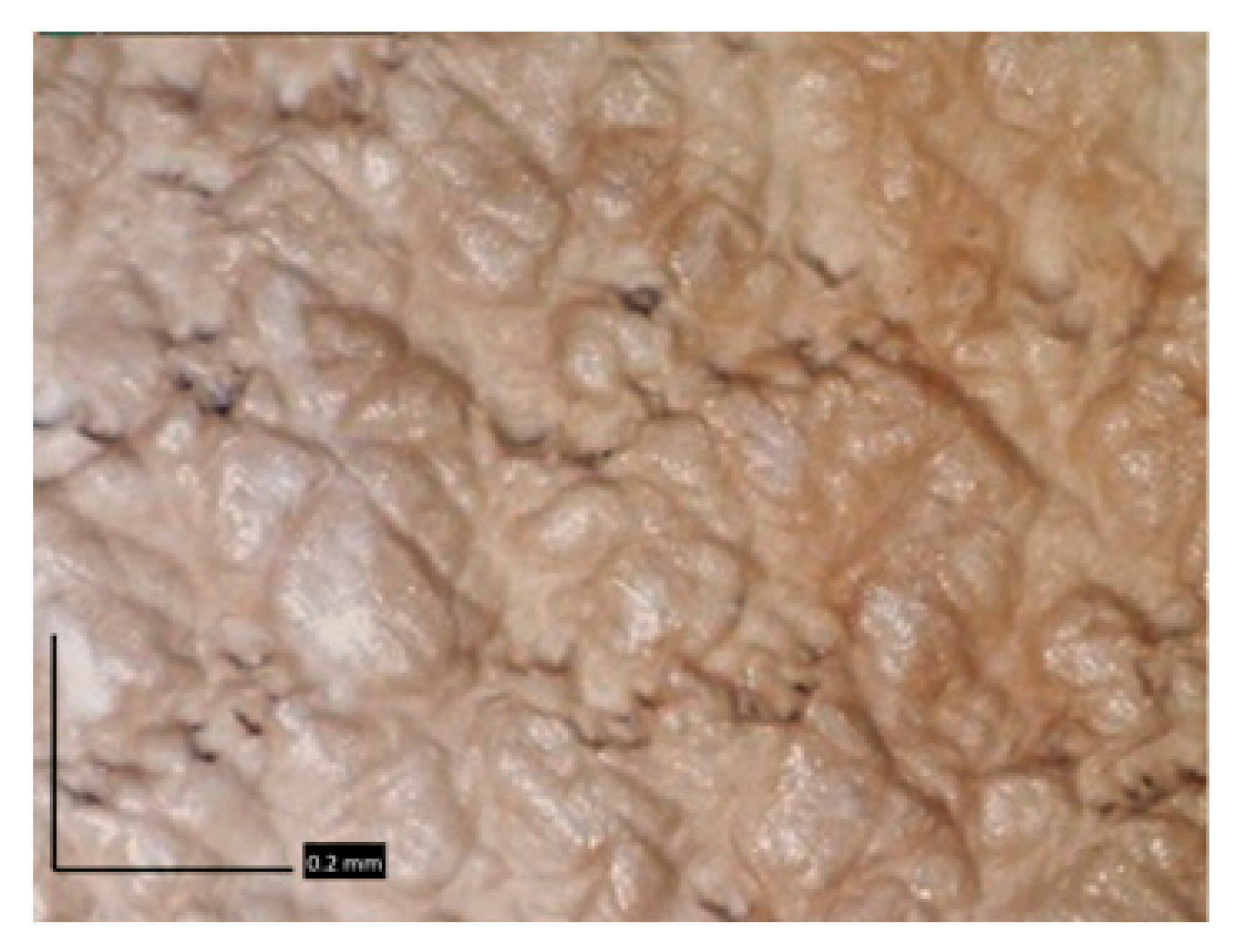	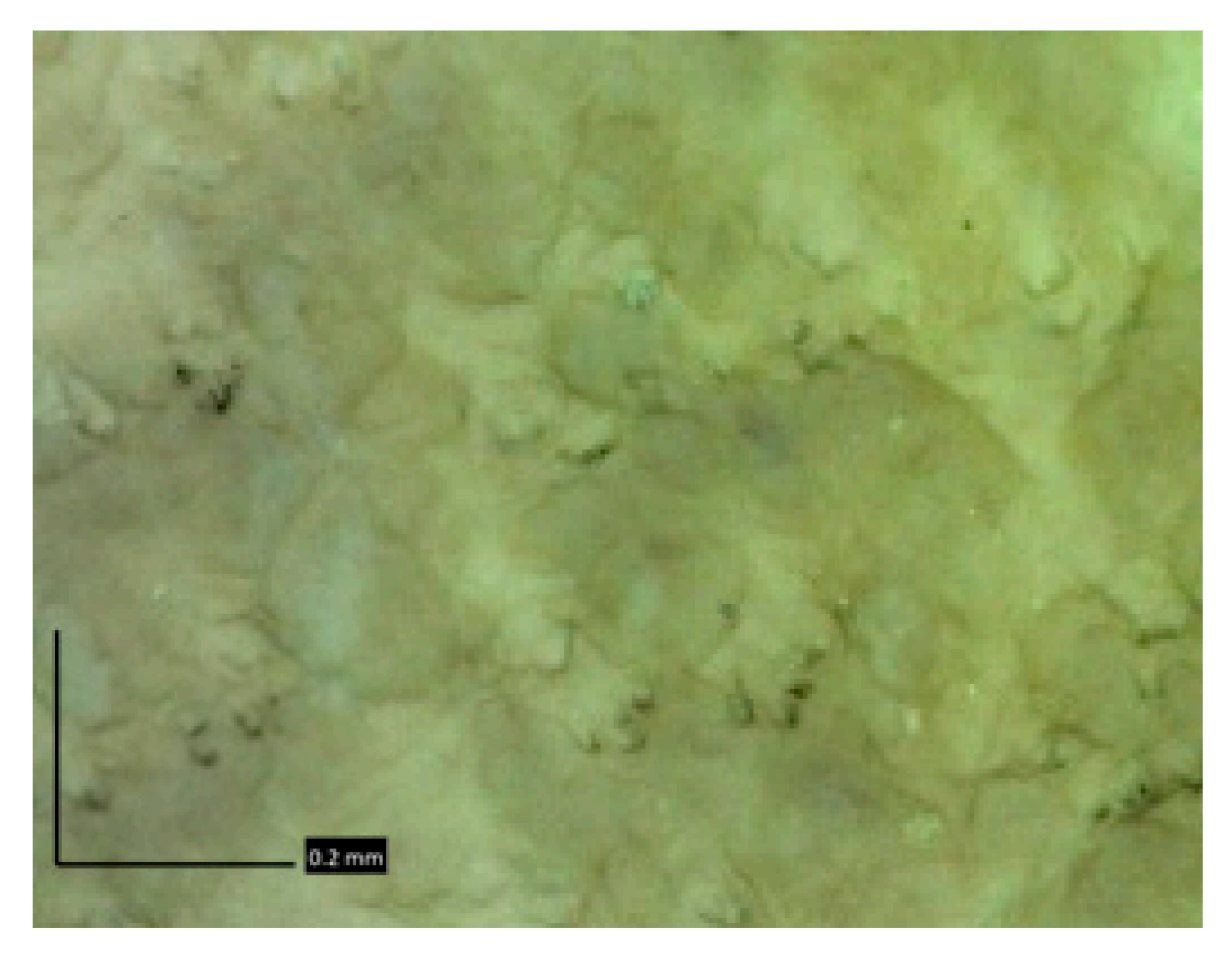
INA_2 g/L	Klucel	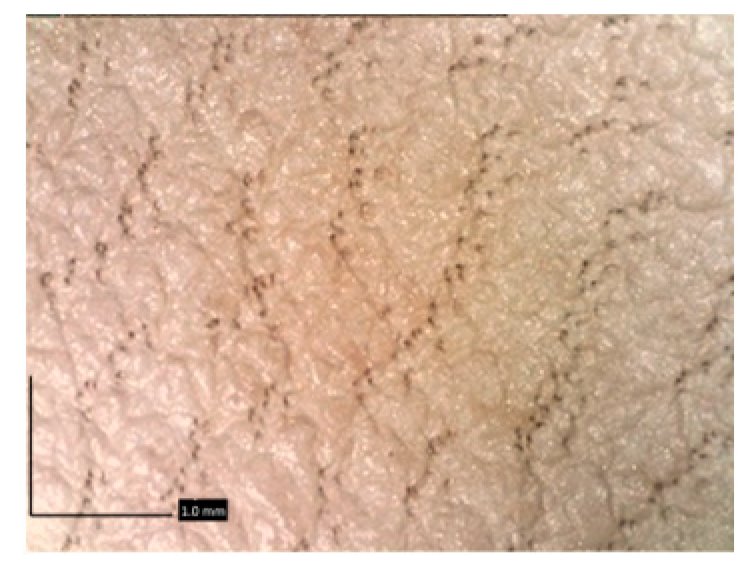	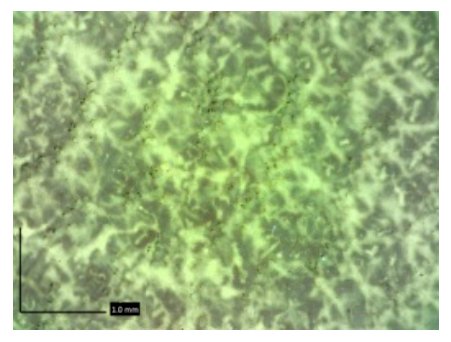	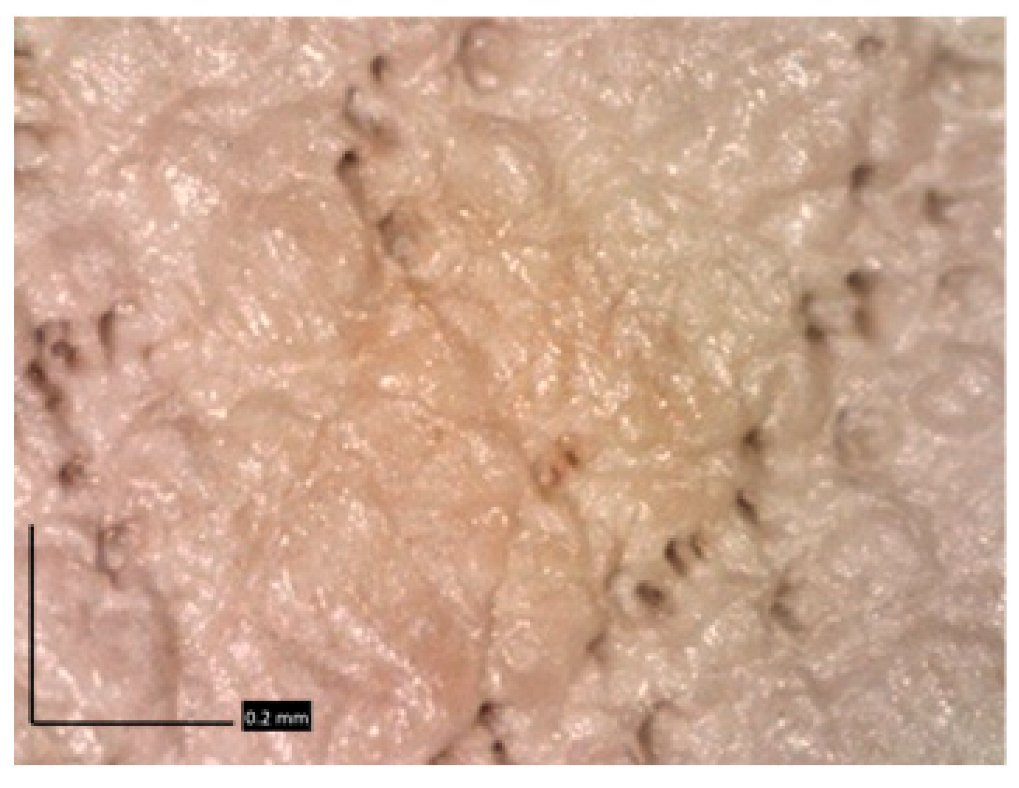	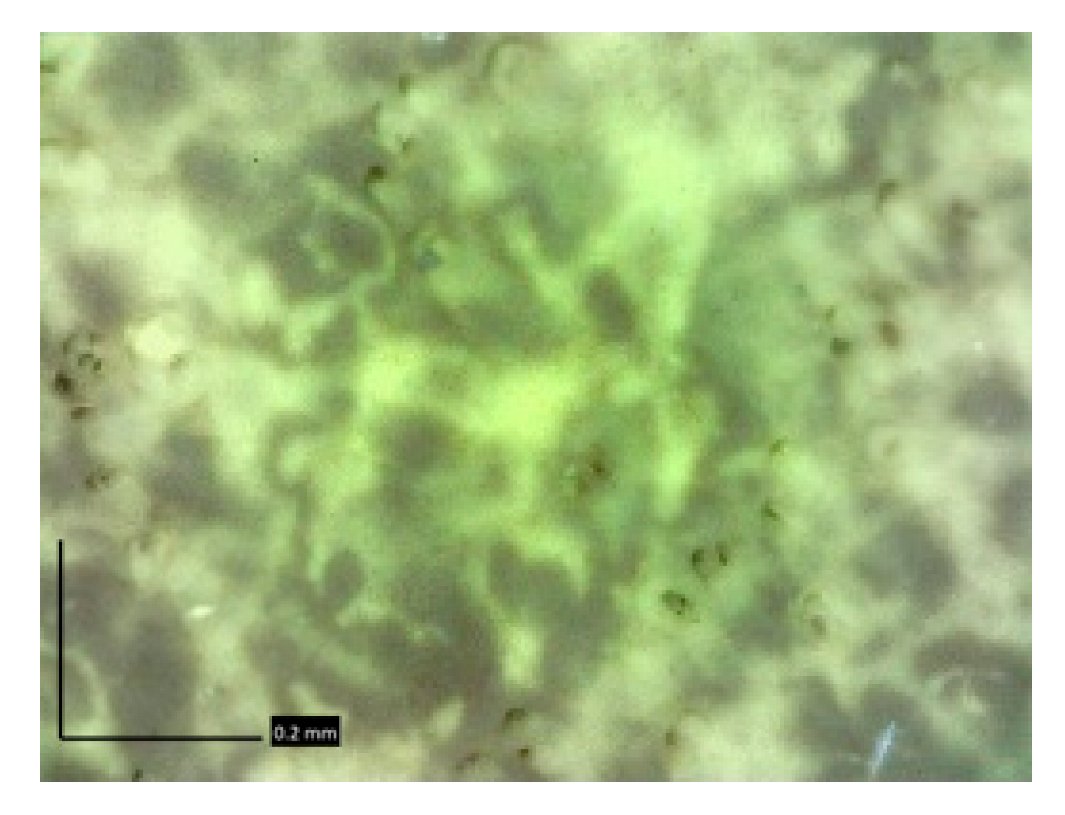
INA_10 g/L	NT	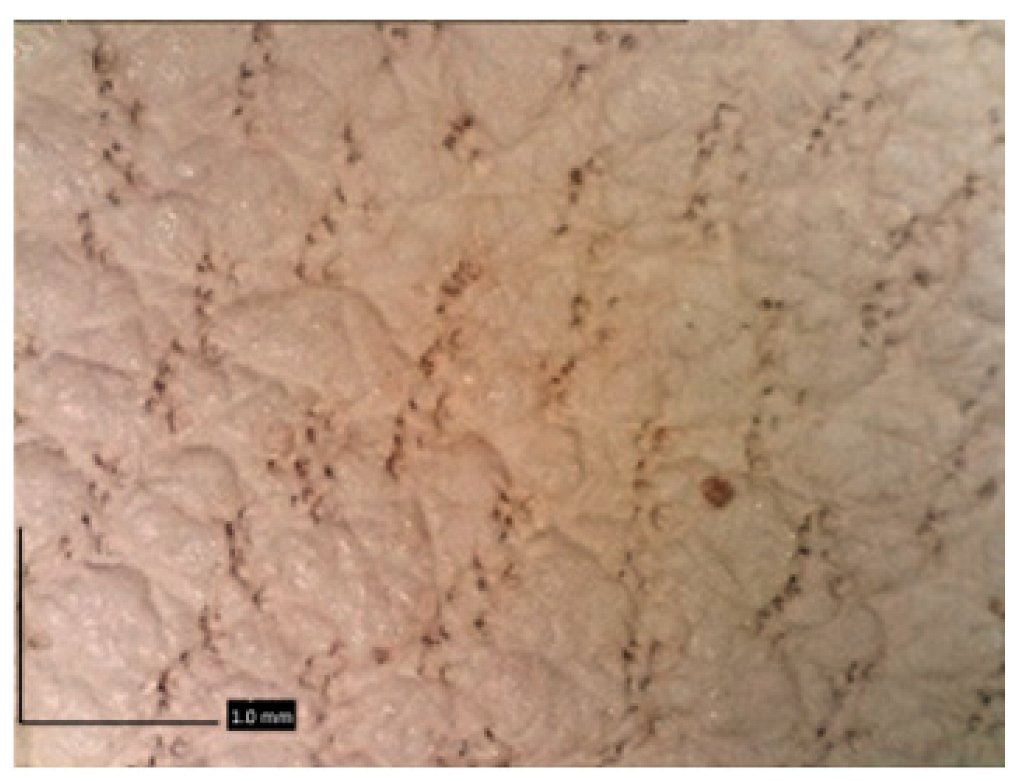	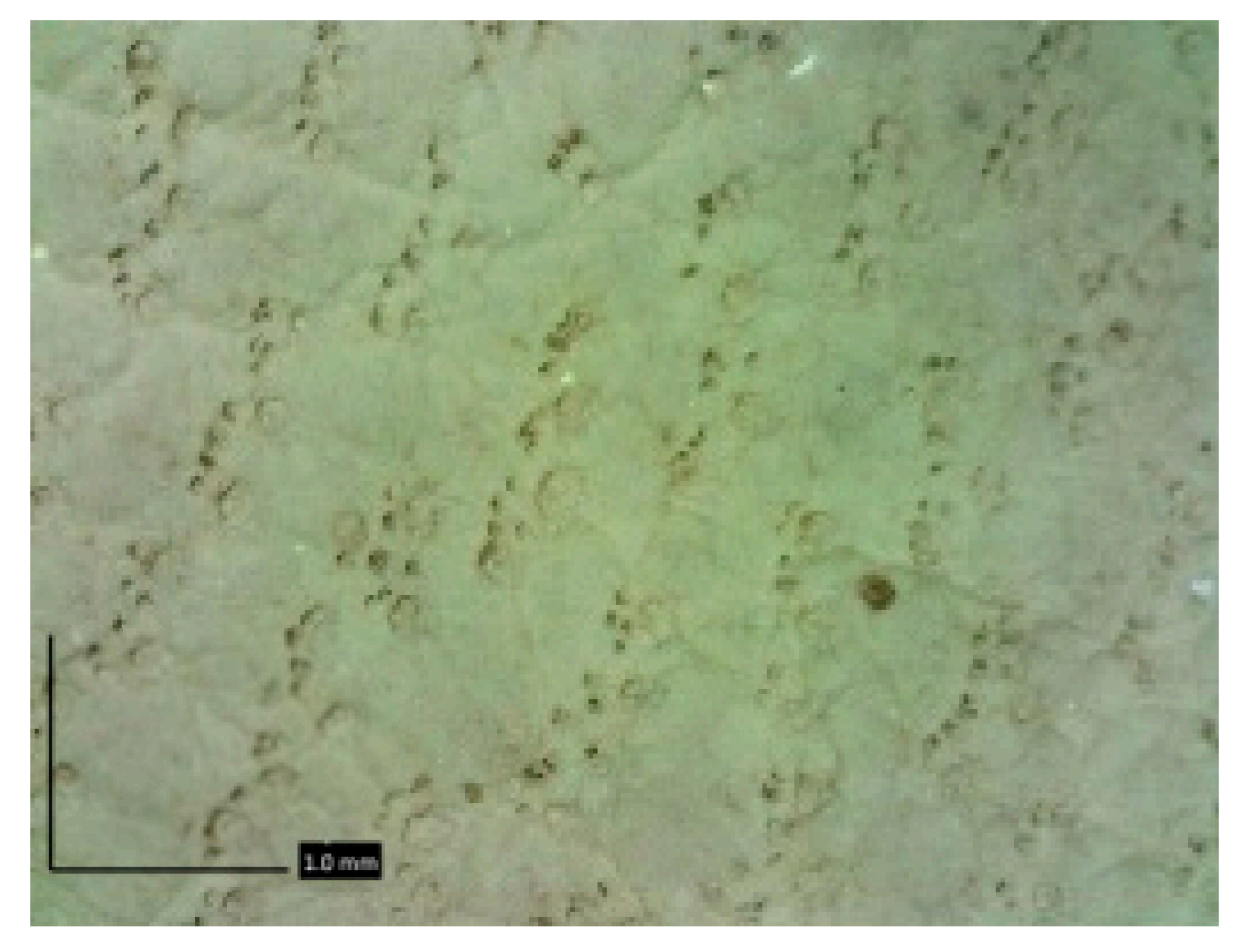	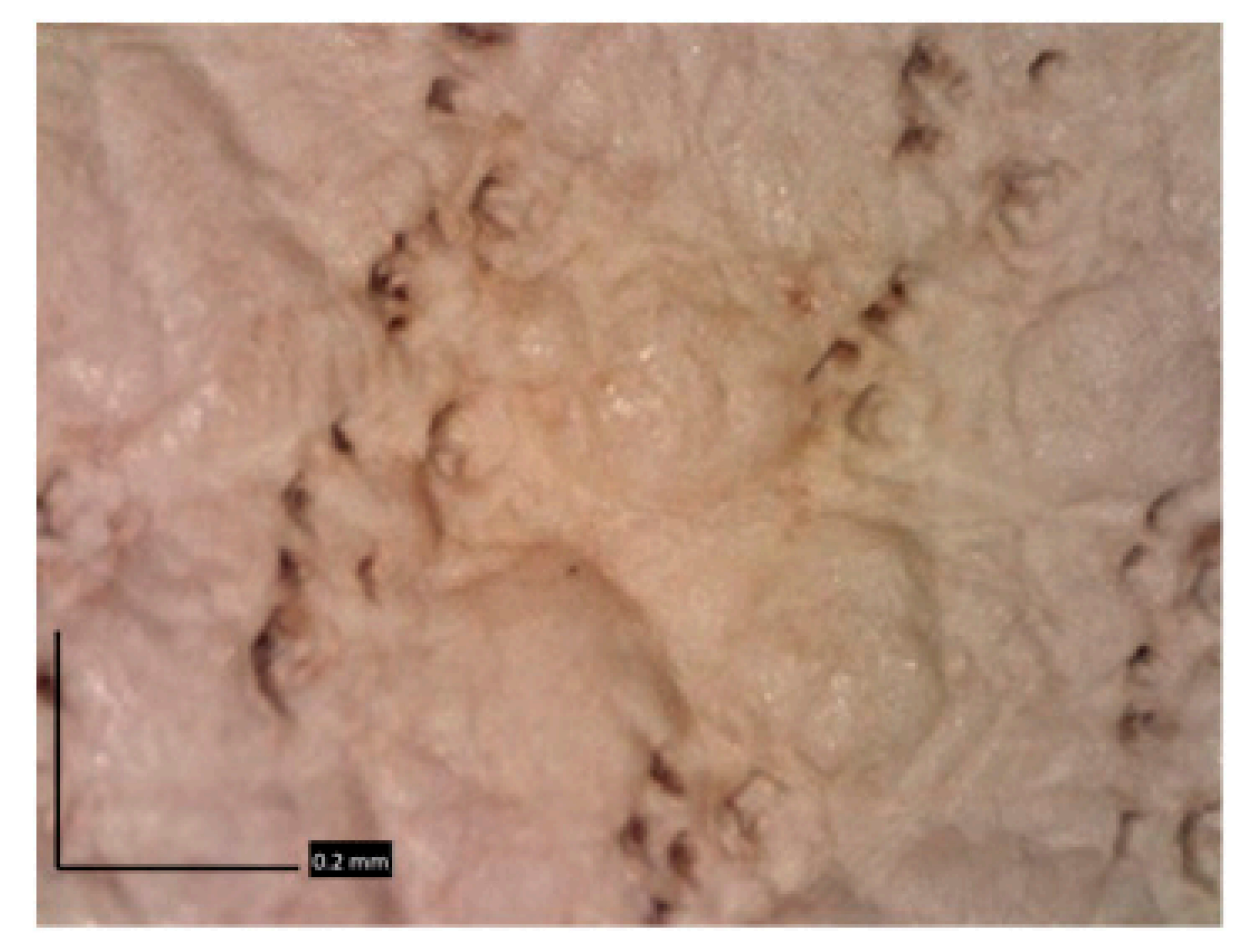	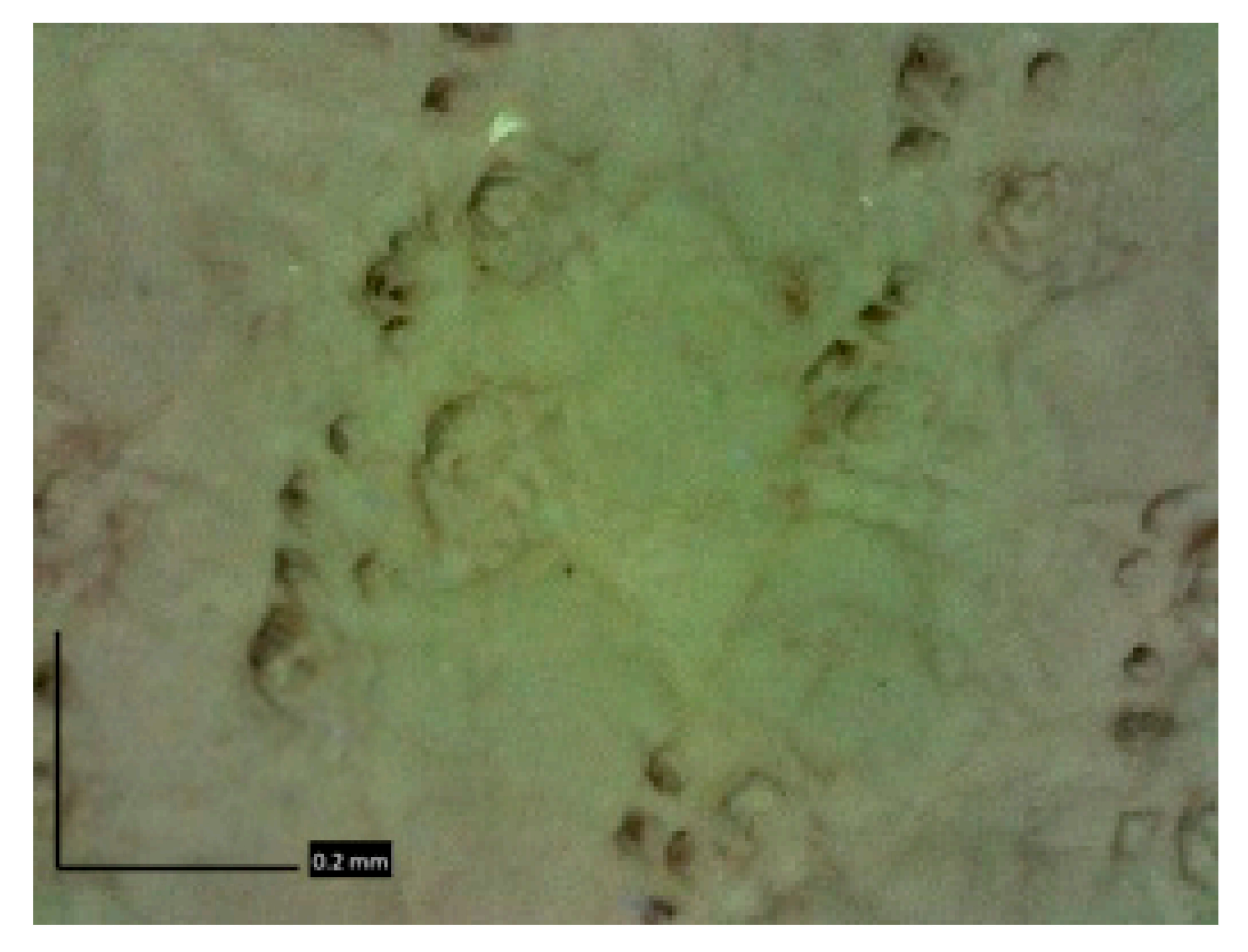
INA_10 g/L	Nanocollagen	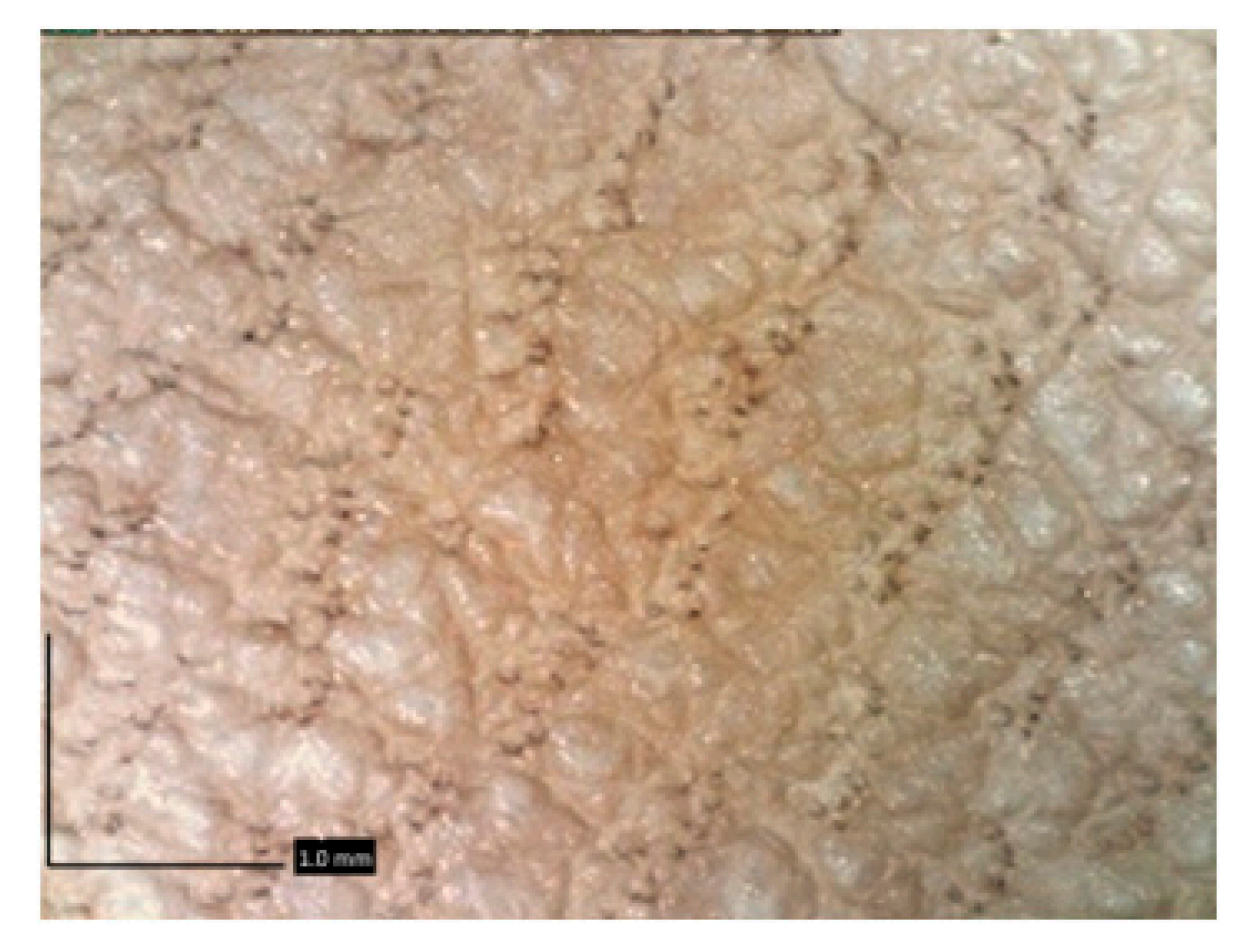	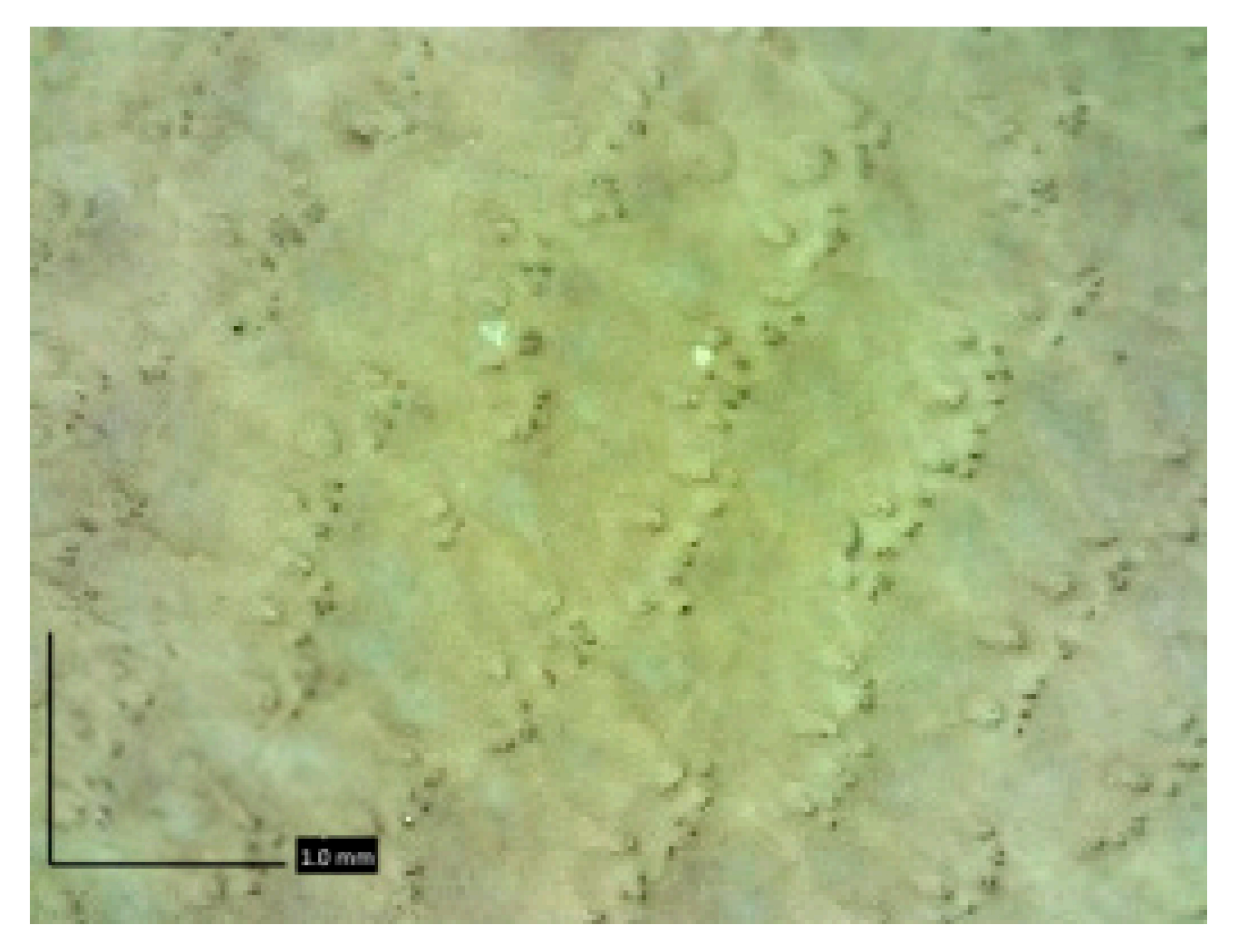	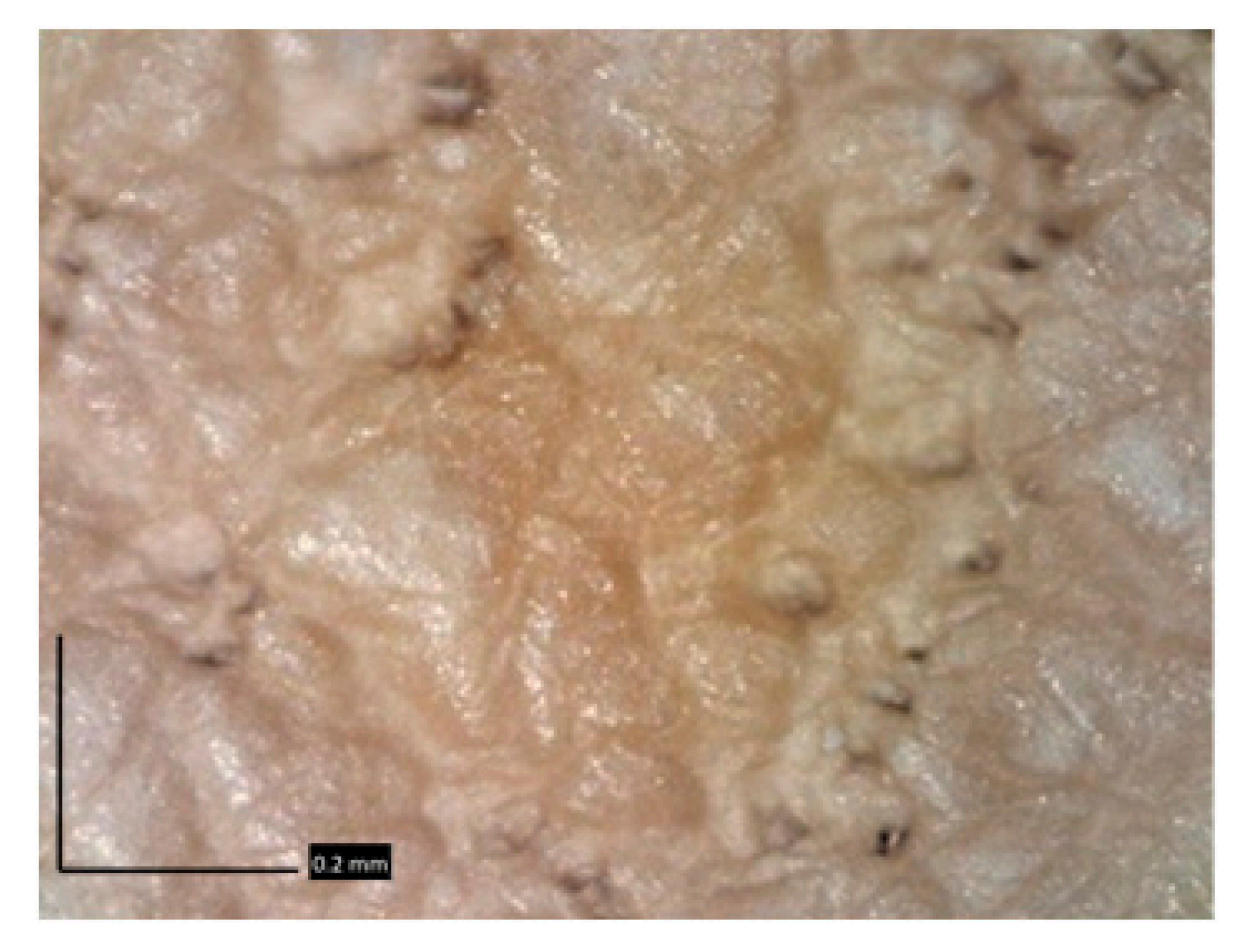	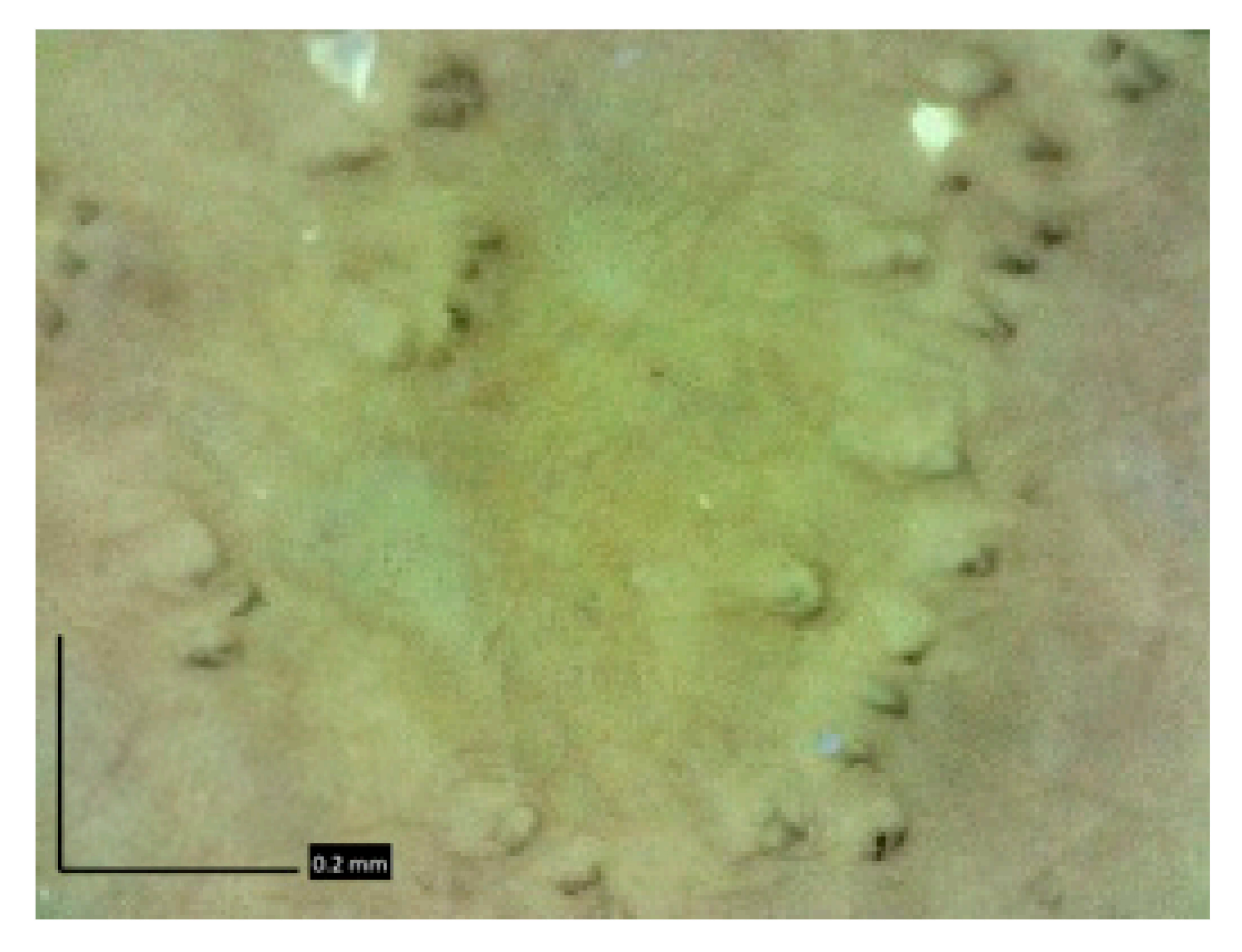
INA_10 g/L	Klucel	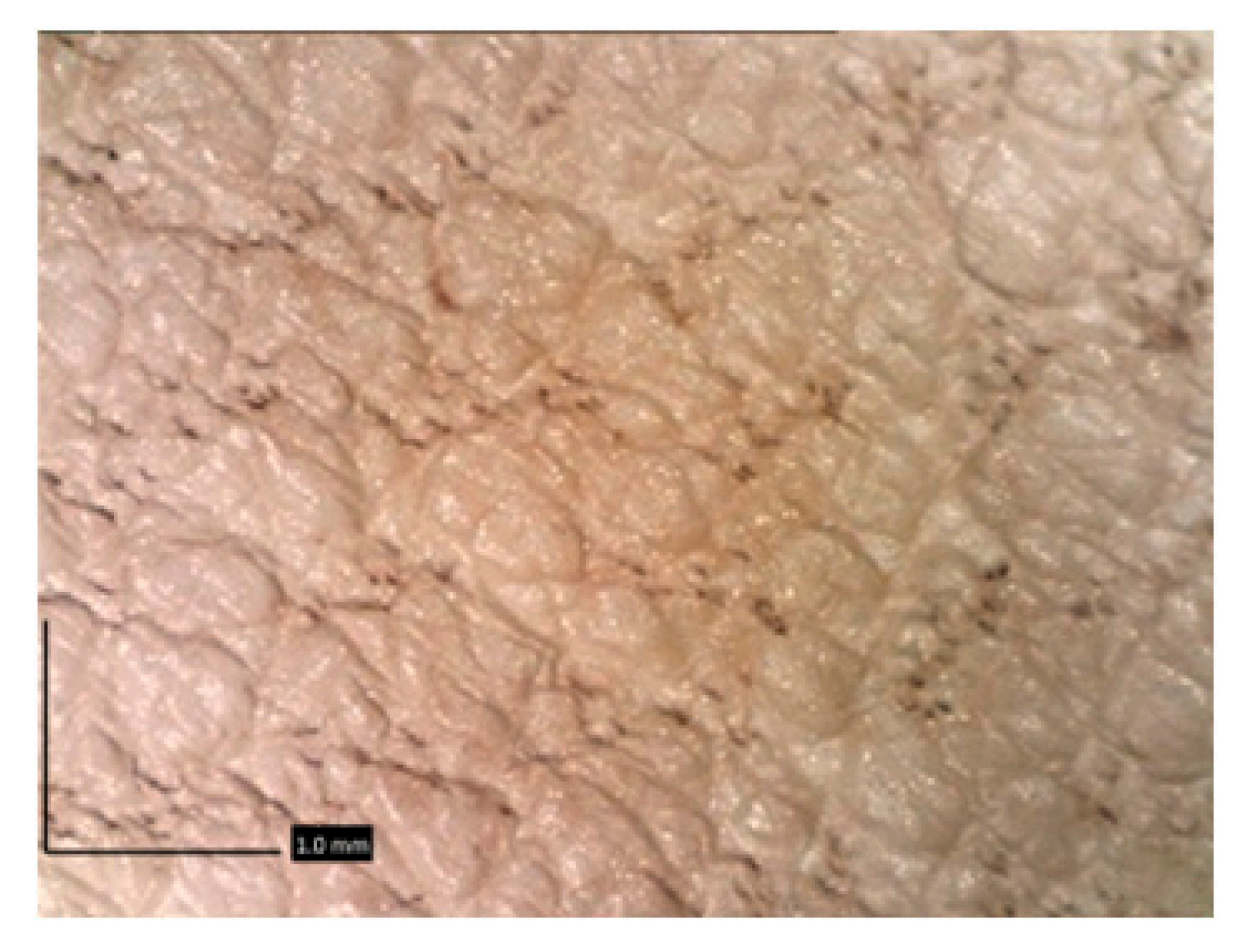	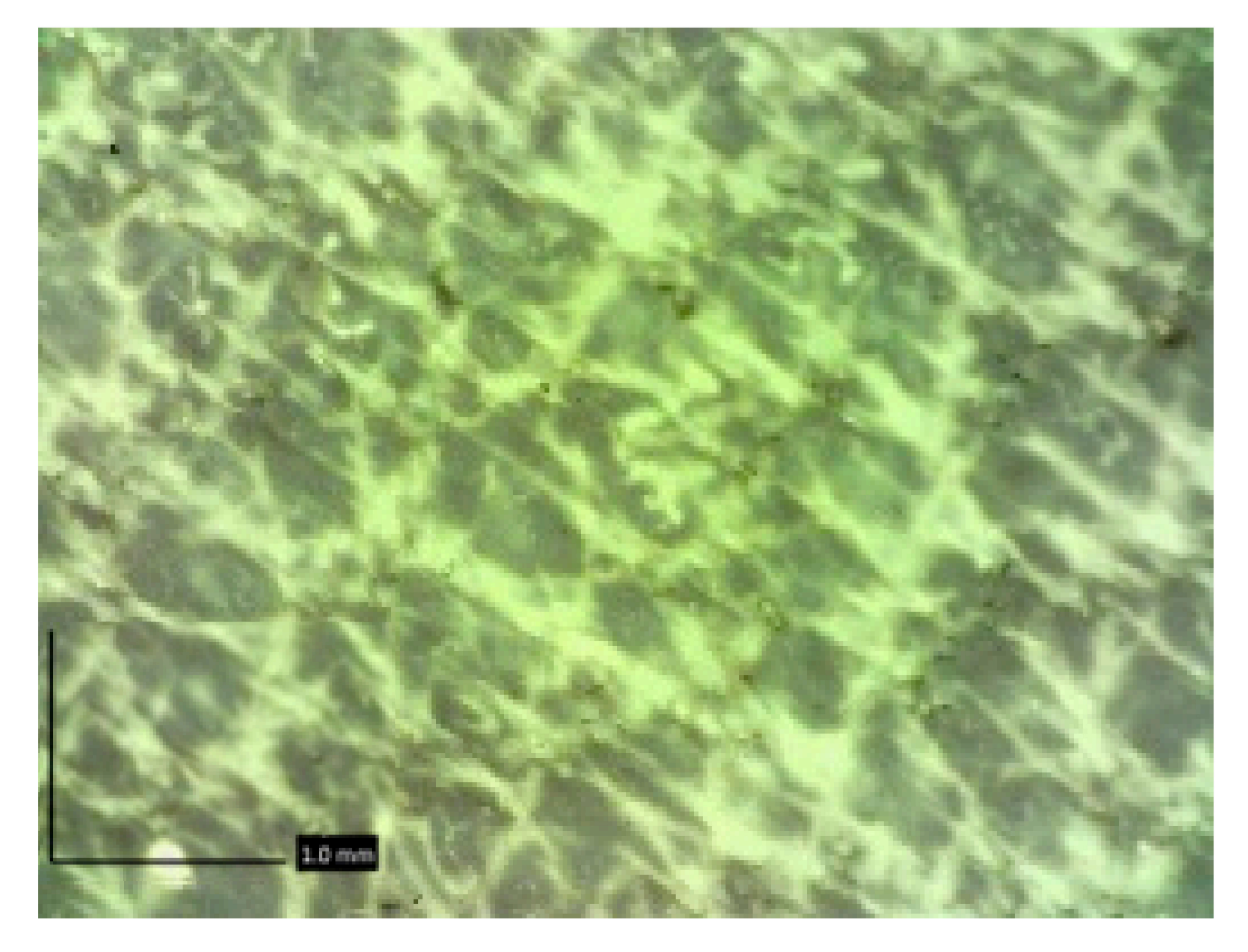	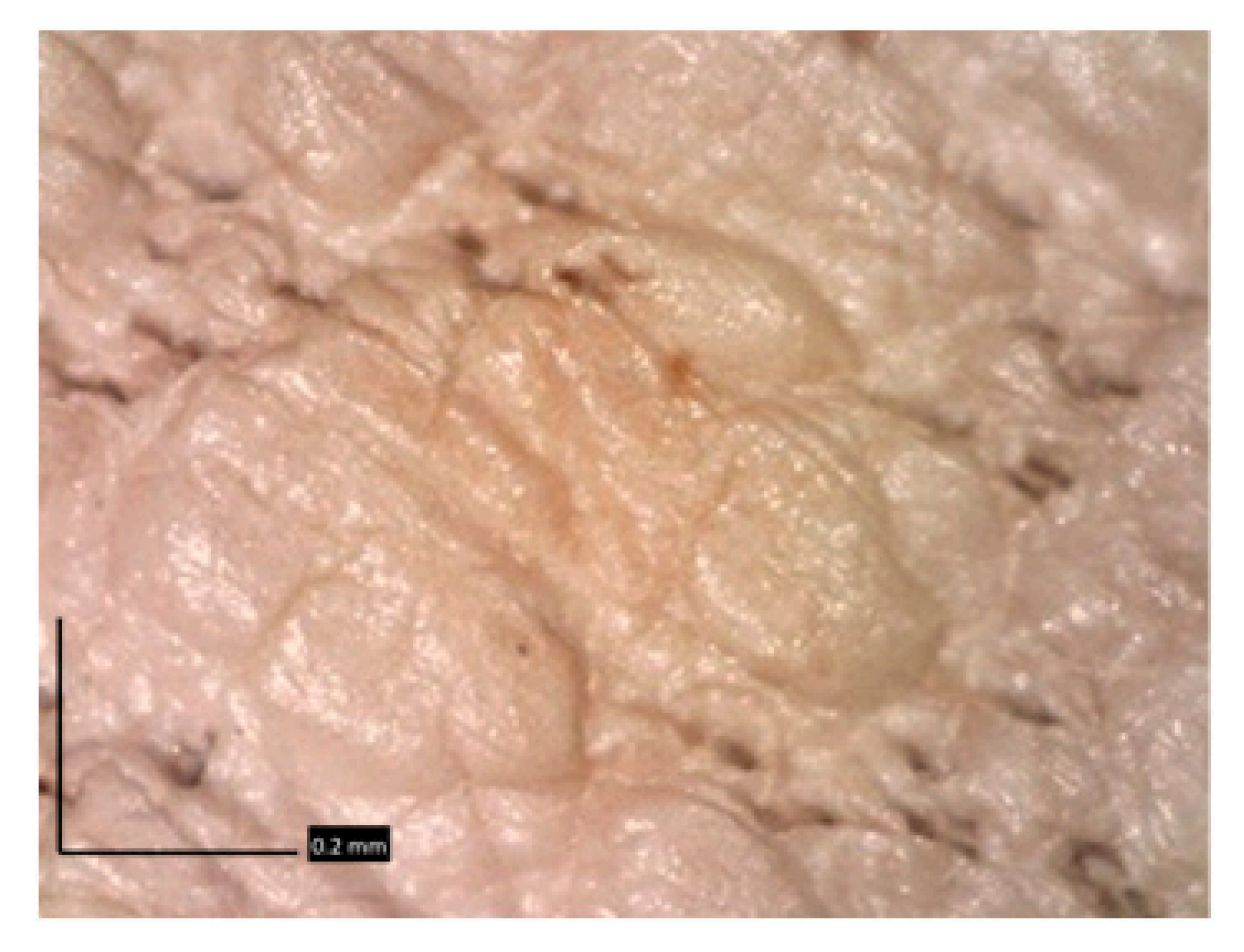	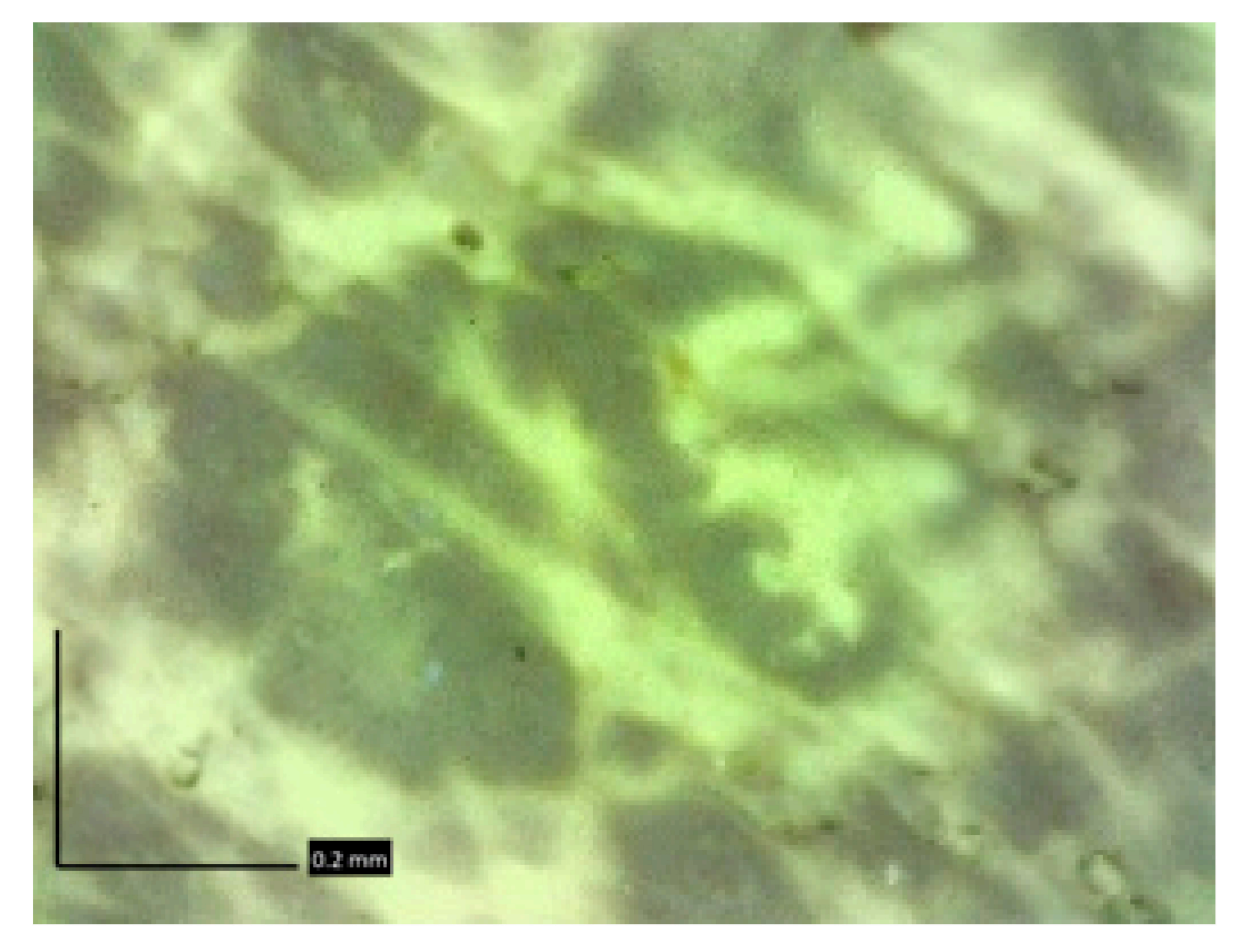

**Table 5:** Mechanical parameters obtained from tensile tests performed on untreated samples and on samples treated with Nanocollagen and Klucel G, under both acidic ageing conditions (presence of HCl) and non-acidic ageing conditions. The reported values include breaking force (F), breaking time (T_breaking_), maximum elongation, maximum stress (Stress_max_) and maximum strain (Strain_max_).

Aging	Camp	F (N)	T_breaking_ (s)	Elongation (mm)	Stress_max_	Strain_max_
IA_2 g/L	NT	291.91 ± 25.13	13.97 ± 1.39	23.24 ± 2.91	19.46 ± 1.67	46.49 ± 5.80
	N	165.81 ± 10.33	25.94 ± 5.70	41.53 ± 9.21	11.05 ± 0.69	83.06 ± 18.39
	K	193.37 ± 3.80	22.54 ± 2.34	37.54 ± 3.81	12.89 ± 0.25	75.07 ± 7.54
IA_10 g/L	NT	185.52 ± 10.40	20.76 ± 1.22	34.57 ± 2.04	13.25 ± 0.69	69.15 ± 4.02
	N	176.64 ± 8.50	19.55 ± 1.20	32.55 ± 2.00	12.62 ± 0.61	65.10 ± 4.00
	K	192.28 ± 13.49	19.38 ± 2.01	32.27 ± 3.35	14.54 ± 0.90	64.53 ± 6.71
INA_2 g/L	NT	352.55 ± 29.51	16.80 ± 2.02	27.96 ± 3.32	25.18 ± 1.95	55.93 ± 6.69
	N	394.45 ± 8.99	17.63 ± 1.22	29.35 ± 2.06	28.17 ± 0.60	58.71 ± 4.12
	K	304.11 ± 42.36	16.41 ± 2.62	27.52 ± 4.27	21.72 ± 2.82	55.05 ± 8.53
INA_10 g/L	NT	312.45 ± 33.17	18.15 ± ± 1.59	30.21 ± 2.59	24.03 ± 2.19	60.43 ± 5.21
	N	348.37 ± 5.28	18.62 ± 1.02	30.98 ± 1.74	24.88 ± 0.35	61.97 ± 3.43
	K	279.27 ± 31.57	15.42 ± 1.32	25.67 ± 2.18	19.95 ± 2.10	51.34 ± 4.35

**Table 6:** CIELab parameters measured on (IA) and (INA) laboratory samples; measurements were acquired before and after Klucel and Nanocollagen treatments, respectively. ΔE values were calculated with respect to the untreated sample. The other parameters, such as ΔC and ΔH, are listed in Table S8 in the Supplementary Materials Section.

Aging	Treatment	L*	a*	b*	ΔE (No Treated-Treated)
IA_2 g/L	NT	70.24 ± 0.47	16.18 ± 0.46	24.35 ± 0.80	-
	Klucel	66.29 ± 2.02	17.68 ± 0.98	27.41 ± 0.58	5.22
	Nanocollagen	67.77 ± 0.60	15.98 ± 0.33	24.55 ± 1.12	2.49
IA_10 g/L	NT	68.05 ± 0.93	17.64 ± 0.47	25.15 ± 0.51	-
	Klucel	66.06 ± 0.33	18.28 ± 0.17	26.49 ± 0.12	2.48
	Nanocollagen	66.80 ± 0.38	17.33 ± 0.50	27.06 ± 0.71	2.30
INA_2 g/L	NT	59.52 ± 0.31	14.57 ± 0.19	22.60 ± 0.38	-
	Klucel	69.45 ± 0.42	14.72 ± 0.25	29.93 ± 0.47	1.34
	Nanocollagen	71.30 ± 0.66	14.75 ± 0.28	24.45 ± 0.20	1.93
INA_10 g/L	NT	69.52 ± 0.22	15.08 ± 0.16	23.83 ± 0.30	-
	Klucel	69.11 ± 0.47	15.46 ± 0.08	25.70 ± 0.20	1.95
	Nanocollagen	68.36 ± 0.08	16.21 ± 0.09	24.25 ± 0.13	1.67

**Table 7:** CIELab parameters measured on the original binding samples before treatment (NT) and after treatments with Klucel G and Nanocollagen. Colour variations (ΔE) were calculated with respect to the untreated sample. Bin. 1: Original Samples collected from Binding 1; Bin. 5: Original Samples collected from Binding 5; Bin. 6: Original Samples collected from Binding 6. The other parameters, such as ΔC and ΔH, are listed in Table S8 in the Supplementary Materials Section.

Sample	Treatment	L*	a*	b*	ΔE (No Treated-Treated)
Bin. 1	NT	34.87 ± 1.61	11.17 ± 0.45	10.26 ± 1.05	-
	Klucel	35.41 ± 0.03	11.47 ± 0.04	12.34 ± 0.03	2.17
	Nanocollagen	32.26 ± 0.64	11.57 ± 0.52	9.32 ±2.28	2.80
Bin. 5	NT	38.93 ± 0.18	14.78 ± 0.38	26.04 ± 1.35	-
	Klucel	38.31 ± 0.01	14.20 ± 0.03	22.79 ± 0.08	3.36
	Nanocollagen	39.55 ± 0.02	14.60 ± 0.05	24.54 ± 0.05	1.62
Bin. 6	NT	37.32 ± 0.03	12.31 ± 0.02	15.44 ± 0.07	-
	Klucel	38.03 ± 0.03	12.82 ± 0.01	16.15 ± 0.06	1.12
	Nanocollagen	35.80 ± 0.08	12.30 ± 0.77	13.75 ± 0.64	2.27

## Data Availability

The data that support the findings of this study are available from the corresponding author upon reasonable request.

## Supplementary Materials

The following supporting information can be downloaded at https://www.mdpi.com/article/10.3390/s26216709/s1. Table S1. FTIR band assignments for Bin. 1 sample. Table S2. FTIR band assignments for Bin. 5 sample. Table S3. FTIR band assignments for Bin. 6 sample. Figure S1. Far-infrared (FIR) spectroscopy for all investigated original samples. Table S4. Far-infrared (FIR) spectroscopy band assignments for all investigated samples. Figure S2. Raman spectrum for all samples, carried out on the hair/grain side. We focused solely on the grain side, as it is more representative of the degradation and allows the signal to stand out more clearly than when superimposed on the signal from the flesh side—which is characterized by higher fluorescence. To overcome this problem and extract significant Raman peaks from the fluorescent background, laboratories employ near-infrared laser excitation sources (e.g., at 1064 nm using the FT-Raman hyphenated technique) or advanced methods such as time-gated Raman spectroscopy. Table S5. Raman bands assignements for all samples. Figure S3. EDX spectrum of Bin. 1 sample. Table S6. Comparison: Nanocollagen vs. Traditional Klucel G: a comparison of sensor devices (portable and non-portable) to evaluate and monitor the performances of novel consolidating agents versus traditional ones. Table S7. (ICE) % index and all the other parameters to evbaluate the consolidation efficiency in the case of the original Bin. 1 sample and the laboratory one sample, called IA_2g/L. As explained in the text, this specific sample was selected from among the laboratory samples because it yields a greater amount of scientifically interesting and useful information following treatment (with Nano-collagen and Klucel G, respectively) and comparison with the laboratory control. Table S8. Data from C* and H* for the more extensive CIELab study.
