# Supplementary material for: Nanocollagen for Historical Leather Conservation: A Case Study on Three Islamic Bookbindings
Source: Sensors (Basel). 2026 Oct 23;26(21):6709. doi: 10.3390/s26216709 (PMC13765650; doi:10.3390/s26216709)
Supplement: Supplementary file 1 [file sensors-26-06709-s001.zip › sensors-4514949-supplementary.pdf]

**Supplementary Materials**

# Nanocollagen for Historical Leather Conservation: A Case Study on Three Islamic Bookbindings

Irene Angela Colasanti[1], Federica Valentini[1*], Alberto Mario Campus[1], Stefania Zeppieri[1], Camilla Zaratti[1,2], Andrea Macchia[2], Michela Relucenti[3], Loredana Cristiano[3], Ilaria Fratoddi[4], Sara Cerra[4]

[1] *Sciences and Chemical Technologies Department, Tor Vergata University, Via della Ricerca Scientifica 1, 00133 Rome, Italy; ireneangela.colasanti@students.uniroma2.eu (I.A.C.); federicavalentini.chem@gmail.com (F.V.); camilla.zaratti@students.uniroma2.eu*
[2] *YOCOCU aps, Via Torquato Tasso 108, 00185 Rome, Italy*
[3] *Department of Anatomical, Histological, Forensic and Orthopedic Sciences, Sapienza University of Rome, Via Alfonso Borelli 50, 00161 Rome, Italy; michela.relucenti@uniroma1.it*
[4] *Department of Chemistry, Sapienza University of Rome, P.le Aldo Moro 5, 00185 Rome, Italy*

**Corresponding author: federicavalentini.chem@gmail.com*

**Table S1**. FTIR band assignments for Bin. 1 sample.

| Flesh side ($cm^{-1}$) | Hair side ($cm^{-1}$) | Assignment | Reference |
|---|---|---|---|
| 3744 | 3744 | ν(Si–OH) | (Volkov et al., 2021) |
| 3273 | 3273 | ν(O–H) of water (free) | (Avram & Mateescu, 1978) |
| 3078 | 3078 | Amide B<br>Fermi resonance overtone of Amide II | (Vyskočilová et al., 2022) |
| 2955 | 2955 | $\nu_{as}$(–$CH_3$) | (Avram & Mateescu, 1978) |
| 2923 | 2923 | $\nu_{as}$(–$CH_2$) | (Avram & Mateescu, 1978) |
| 2854 | 2854 | $\nu_s$(–$CH_2$) | (Avram & Mateescu, 1978) |
| 1649 | 1649 | Amide II (AI)<br>ν(C=O) | (Kristoffersen et al., 2023) |
| 1637 | 1637 | ν(C=O) of Amide II (AII) | (Kristoffersen et al., 2023) |
| 1543 | 1543 | δ(N–H) e ν(C–N) of Amide II (AII) | (Vyskočilová et al., 2022) |
| 1457 | 1457 | $\delta_{as}$(C–H) deformation of (–$CH_3$) | (Avram & Mateescu, 1978) |
| 1436 | 1436 | ν(–C=C) aromatic | (Vichi et al., 2018) |
| 1420 | 1420 | $\nu_{as}$(–$CO_3^{2-}$) of carbonates | (Cai et al., 2010; Vichi et al., 2018) |
| 1396 | 1396 | $\nu_{as}$(–$CO_3^{2-}$) of carbonates | (Vichi et al., 2018) |
| 1375 | 1375 | δ(–$CH_2$, –$CH_3$) | (Vyskočilová et al., 2022) |
| 1364 | 1364 | δ/ω(–CH/–$CH_2$) | (Castrovilli et al., 2025) |
| 1338 | 1338 | δ(–OH/–$CH_2$) | (Castrovilli et al., 2025) |
| 1320 | 1320 | $\nu_{as}$(C–O–C) and (O–H) def. hydrolysable tannins | (Ricci et al., 2015) |
| 1276 | 1276 | δ(N–H) e ν(C–N) of Amide III (AIII) | (Castrovilli et al., 2025) |
| 1237 | 1237 | $\nu_s$(C–O–C)<br>+<br>$\nu_{as}$(–$SO_4^{2-}$) | (Vyskočilová et al., 2022; Wijayati et al., 2021) |
| 1200 | 1200 | ν(S=O) | (Wijayati et al., 2021) |
| 1150 | 1150 | δ(C–OH) of condensed tannins | (Koochakzaei & Sabaghian, 2023) |
| 1107 | 1107 | $\nu_s$(–$SO_4^{2-}$) | (Wijayati et al., 2021) |
| 1074 | 1074 | ν(S=O)<br>+<br>$N_s$(C–O–C) of aryl phenolic ester of gallotannins | (Vyskočilová et al., 2022; Wijayati et al., 2021) |
| 1043 | 1043 | ν(Si–O) of aluminosilicates | (China et al., 2019) |
| 1027 | 1027 | ν(Si–O) of aluminosilicates | (China et al., 2019) |
| 1006 | 1006 | ν(Si–O) of aluminosilicates | (China et al., 2019) |
| 920 | 920 | Asym. def. of the α-D-glucopyranose ring (band type 1) of starch | (Castrovilli et al., 2025) |
| 875 | 875 | ν(C–C)<br>+<br>δ(Al–O) | (Johnsen et al., 2004; Koochakzaei & Sabaghian, 2023) |
| 860 | 860 | ν(Al–O)<br>+<br>Asym. def. of the α-D-glucopyranose ring (band type 2) of starch | (China et al., 2019) |
| 776+760 | 776+760 | Al–O complex<br>+<br>$\nu_s$ skeletal sugar ring breathing vibration of gallotannins and of starch (band type 3) | (Saniger, 1995) |

**Table S2.** . FTIR band assignments for Bin. 5 sample.

| Flesh side ($cm^{-1}$) | Hair side ($cm^{-1}$) | Assignment | Reference |
|---|---|---|---|
| 3737 | 3737 | ν(Si–OH) | (Volkov et al., 2021) |
| 3283 | 3283 | ν(O–H) of water (free) | (Avram & Mateescu, 1978) |
| 3078 | 3078 | Amide B<br>Fermi resonance overtone of Amide II | (Vyskočilová et al., 2022) |
| - | 2960 | $\nu_{as}$(–$CH_3$) | (Avram & Mateescu, 1978) |
| 2923 | 2923 | $\nu_{as}$(–$CH_2$) | (Avram & Mateescu, 1978) |
| 2854 | 2854 | $\nu_{s}$(–$CH_2$) | (Avram & Mateescu, 1978) |
| 1649 | 1649 | ν(C=O) of amide I (AI) | (Kristoffersen et al., 2023) |
| 1633 | 1633 | ν(C=O) of amide I (AI) | (Kristoffersen et al., 2023) |
| 1543 | 1543 | δ(N–H) e ν(C–N) of amide II (AII) | (Vyskočilová et al., 2022) |
| 1450 | 1450 | $\delta_{as}$(C–H) def. of (–$CH_3$) | (Avram & Mateescu, 1978) |
| 1400 | 1400 | δ(C–OH) of photooxidation of carboxylic acids | (Vyskočilová et al., 2019) |
| 1374 | 1374 | δ(–$CH_2$, –$CH_3$) | (Ricci et al., 2015) |
| 1327 | 1327 | $\nu_{as}$(C–O–C) and (O–H) def. of hydrolysable tannins | (Koochakzaei & Sabaghian, 2023) |
| 1280 | 1280 | ν(phenyl-O) of condensed tannins | (Koochakzaei & Sabaghian, 2023; Ricci et al., 2015) |
| 1237 | 1237 | δ(N–H) and ν(C–N) of amide III (AIII) + small contribution of $\delta_{ip}$(C=O) and<br>ν(C–C)<br>+<br>$\nu_{as}$(–$SO_4^{2-}$) | (Vyskočilová et al., 2022; Wijayati et al., 2021) |
| 1205 | 1205 | ν(S=O) | (Wijayati et al., 2021) |
| 1155 | 1155 | δ(C–OH) of condensed tannins | (Koochakzaei & Sabaghian, 2023) |
| 1105 | 1105 | $\nu_{s}$(–$SO_4^{2-}$) | (Wijayati et al., 2021) |
| 1075 | 1075 | ν(S=O)<br>+<br>$\nu_{s}$(C–O–C) of aryl phenolic ester of gallotannins | (Vyskočilová et al., 2022; Wijayati et al., 2021) |
| 1032 | 1032 | ν(Si–O) of aluminosilicates | (China et al., 2019) |
| 1021 | - | ν(Si–O) of aluminosilicates | (China et al., 2019) |
| 1004 | - | ν(Si–O) of aluminosilicates | (China et al., 2019) |
| 924 | 924 | Asym. def. of the $\alpha$-D-glucopyranose ring (band type 1) of starch | (Castrovilli et al., 2025; Volkov et al., 2021) |
| 876 | 876 | ν(C–C)<br>+<br>δ(Al–O) | (Johnsen et al., 2004; Koochakzaei & Sabaghian, 2023) |
| 865 | 865 | ν(A–O)<br>+<br>Asym. def. of the $\alpha$-D-glucopyranose ring (band type 2) of starch | (Castrovilli et al., 2025; China et al., 2019) |
| 840 | - | $\delta_{op}$(C–H) of aromatic condensed tannins | (Koochakzaei & Sabaghian, 2023) |
| 775+757 | 775+757 | Complex Al–O<br>+<br>$\nu_{s}$ skeletal sugar ring breathing vibration of gallotannins and of starch (band type 3) | (Saniger, 1995) |
| 680 | - | δ(Al–O) | (China et al., 2019) |
| 668 | 668 | $\delta_{s}$(–$SO_4^{2-}$)<br>+<br>δ(O–H) | (Castrovilli et al., 2025) |
| 655 | 655 | δ(C–H) alifatic | (Avram & Mateescu, 1978) |
| 642 | 642 | δ(O–Si(Al–O) | (Theodosoglou et al., 2017) |

**Table S3.** . FTIR band assignments for Bin. 6 sample.

| Flesh side ($cm^{-1}$) | Hair side ($cm^{-1}$) | Assignment | Reference |
|---|---|---|---|
| 3737 | - | ν(Si–OH) | (Volkov et al., 2021) |
| 3530 | - | ν(O–H) of polyphenolic compounds | (Ricci et al., 2015) |
| 3396 | - | ν(N–H) of Amide A | (Vyskočilová et al., 2022) |
| 3280 | 3280 | ν(O–H) of bonded water | (Avram & Mateescu, 1978) |
| 3068 | 3068 | Amide B<br>Fermi resonance overtone of Amide II | (Vyskočilová et al., 2022) |
| 2960 | 2960 | $\nu_{as}(-CH_3)$ | (Avram & Mateescu, 1978) |
| 2920 | 2920 | $\nu_{as}(-CH_2)$ | (Avram & Mateescu, 1978) |
| 2850 | 2850 | $\nu_{s}(-CH_2)$ | (Avram & Mateescu, 1978) |
| 1648 | 1648 | ν(C=O) of Amide I (AI) | (Kristoffersen et al., 2023) |
| 1630 | 1630 | ν(C=O) of Amide I (AI) | (Vyskočilová et al., 2022) |
| 1540 | 1540 | δ(N–H) and ν(C–N) of Amide II (AII) | (Vyskočilová et al., 2022) |
| 1446 | 1446 | $\delta_{as}$(C–H) def. $(-CH_3)$ | (Avram & Mateescu, 1978) |
| 1410 | 1410 | δ(C–OH) of photooxidation of carboxylic acids | (Vyskočilová et al., 2019) |
| 1375 | 1375 | $\delta(-CH_2, -CH_3)$ | (Ricci et al., 2015) |
| 1320 | 1336+1320 | $\nu_{as}$(C–O–C) and (O–H) def. hydrolysable tannins | (Koochakzaei & Sabaghian, 2023) |
| 1277 | 1277 | ν(phenyl-O) of condensed tannins | (Koochakzaei & Sabaghian, 2023; Ricci et al., 2015) |
| 1235 | 1235 | δ(N–H) and ν(C–N) of Amide III (AIII)<br>+<br>small contribution of $\delta_{ip}$(C=O) and<br>ν(C–C)<br>+<br>$\nu_{as}(-SO_4^{2-})$ | (Vyskočilová et al., 2022; Wijayati et al., 2021) |
| 1200 | 1200 | ν(S=O) | (Wijayati et al., 2021) |
| 1157 | - | δ(C–OH) of condensed tannins | (Koochakzaei & Sabaghian, 2023) |
| - | 1150 | δ(C–OH) of condensed tannins | (Koochakzaei & Sabaghian, 2023) |
| 1107 | 1107 | $\nu_{s}(-SO_4^{2-})$ | (Wijayati et al., 2021) |
| 1078 | 1078 | ν(S=O)<br>+<br>$\nu_{s}$(C–O–C) of aryl phenolic ester of gallotannins | (Vyskočilová et al., 2022; Wijayati et al., 2021) |
| 1032 | 1032 | ν(Si–O) of aluminosilicates | (Wijayati et al., 2021) |
| 875 | 875 | ν(C–C)<br>+<br>δ(Al–O) | (China et al., 2019; Koochakzaei & Sabaghian, 2023) |
| 776 | 776 | Complex Al–O<br>+<br>$\nu_{s}$ (breathing) of skeletal sugar ring vibration of gallotannins | (China et al., 2019) |
| 670 | 670 | $\delta_{s}(-SO_4^{2-})$ | (Wijayati et al., 2021) |
| 655 | 655 | $\delta_{op}$(C–OH) of phenolic ring | (Ricci et al., 2015) |
| 648+640 | 648+640 | $\delta_{op}$ (C–OH) of phenolic ring | (Ricci et al., 2015) |

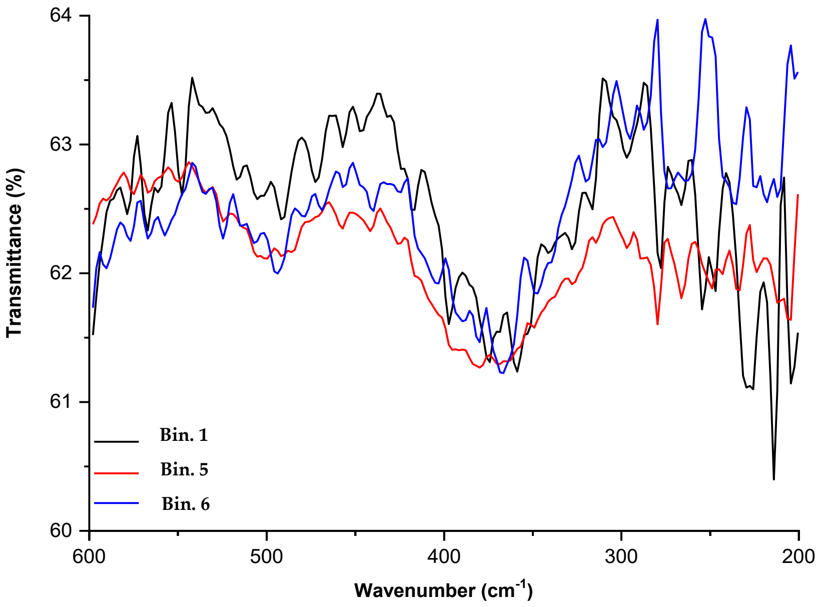


**Figure S1**. Far-infrared (FIR) spectroscopy for all investigated original samples.

**Table S4**. Far-infrared (FIR) spectroscopy band assignments for all investigated samples

| **Bin. 1 ($cm^{-1}$)** | **Bin. 5 ($cm^{-1}$)** | **Bin. 6 ($cm^{-1}$)** | **Assignment** | **Ref.** |
|---|---|---|---|---|
| 560 | - | 560 | δ(Al–OH), δ(C–C–O) | (Ricci et al., 2015; Volkov et al., 2021) |
| - | 525 | 525 | $\nu_s[Al(H_2O)_6]^{3+}$ dal $KAl(SO_4)_2{\cdot}12(H_2O)$ | (Vyskočilová et al., 2022) |
| 496 | 496 | 496 | $\nu_s$(Al–O) | (Saniger, 1995) |
| 370 | 380 | 376 | δ(C–C–C) | (Ruan et al., 2002) |
| 274 | 274 | 268 | Def. $[Al(H_2O)_6]^{3+}$ in $KAl(SO_4)_2{\cdot}12(H_2O)$ | (Barashkov et al., 2004) |
| 251 | 249 | 239 | $\nu_7$(Al–O) | (Ruan et al., 2002) |
| 220 | - | 230 | Def. ($-CO_3^{2-}$) | (Avram & Mateescu, 1978) |
| 205 | 208 | - | $\nu_9$(Al–O) | (Ruan et al., 2002) |

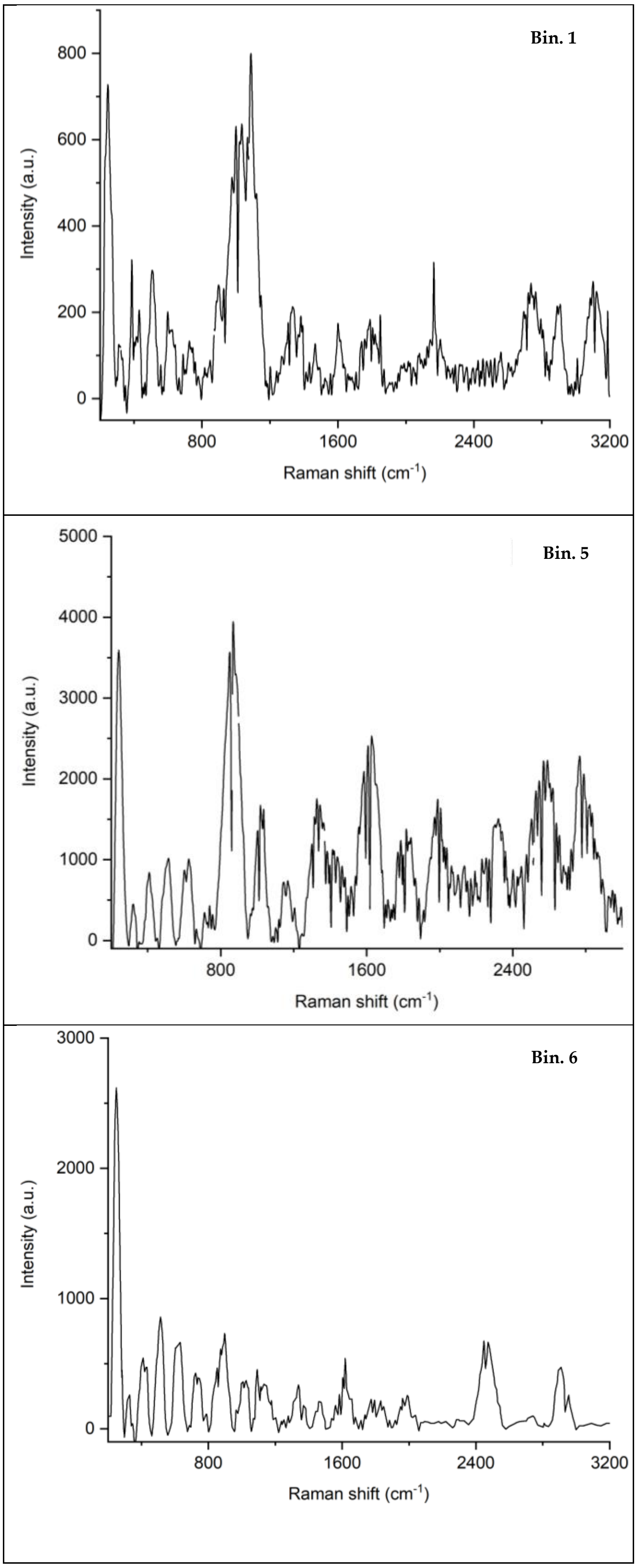


**Figure S2.** Raman spectrum for all samples, carried out on the hair/grain side. We focused solely on the grain side, as it is more representative of the degradation and allows the signal to stand out more clearly than when superimposed on the signal from the flesh side—which is characterized by higher fluorescence. To overcome this problem and extract significant Raman peaks from the fluorescent background, laboratories employ near-infrared laser excitation sources (e.g., at 1064 nm using the FT-Raman hyphenated technique) or advanced methods such as time-gated Raman spectroscopy.

**Table S5.** Raman bands assignements for all samples

| Bin. 1 ($cm^{-1}$) | Bin. 5 ($cm^{-1}$) | Bin. 6 ($cm^{-1}$) | Assignment | References |
|---|---|---|---|---|
| 249 | 242 | 248 | Lattice mode of silicates + δ(CCC) of gallic acid | (Espina et al., 2022; Palmeri et al., 2009; Sharma et al., 2006; Wang et al., 2015) |
| 316 | 323 | 328 | Lattice mode of silicates + $\delta_{op}$(CO), $\delta_{op}$(ring) of tannins | (Espina et al., 2022; Palmeri et al., 2009; Sharma et al., 2006; Wang et al., 2015) |
| 390 | 408 | 403 | Collagen skeleton def. + $A_{1g}$ $Al_2O_3$ | (Cárcamo et al., 2012; Delbé et al., 2022; Malea et al., 2024; Mandair & Morris, 2015) |
| 427 | - | 427 | $\nu_2$ bending ($SO_4^{2-}$)+ lattice modes of silicates + $E_g$ $Al_2O_3$ | (Barashkov et al., 2004; Delbé et al., 2022; Palmeri et al., 2009; Sharma et al., 2006; Wang et al., 2015) |
| 501 | 512 | 511 | $\nu_s$(Si–O–Si/Si–O–Al) K-feldspar + $\delta_{op}$(ring) of tannins | (Espina et al., 2022; Palmeri et al., 2009; Sharma et al., 2006; Wang et al., 2015) |
| 598 | 598 | 600 | ν(C–S) of cysteine/cystine and methionine residue (collagen) + $\delta_{op}$(CO), ν(ring) of pyrogallol | (Cárcamo et al., 2012; Espina et al., 2022; Malea et al., 2024; Mandair & Morris, 2015) |
| 628 | 620 | 626 | δ($SO_4^{2-}$) | (Barashkov et al., 2004) |
| 639 | - | - | ϱ, δ, ω (COO) of collagen | (Cárcamo et al., 2012; Malea et al., 2024; Mandair & Morris, 2015) |
| 683 | - | - | $\delta_{op}$(OH) of tannins | (Espina et al., 2022) |
| 724 | 706 | 722 | $\nu_4$($CO_3^{2}$) + ν(ring) of gallic acid | (Espina et al., 2022; Mandair & Morris, 2015) |
| 743 | 747 | 746 | $E_g$ $Al_2O_3$ + $\delta_{op}$(CH), $\delta_{op}$(OH) of tannins | (Delbé et al., 2022; Espina et al., 2022) |
| 780 | - | 786 | δ($COO^-$) of collagen + $\delta_{op}$(CH), $\delta_{op}$(OH) of tannins | (Cárcamo et al., 2012; Espina et al., 2022; Malea et al., 2024; Mandair & Morris, 2015) |
| 817 | - | - | $\delta_{op}$(CH) of tannins | (Espina et al., 2022) |
| 847 | - | 849 | ν(C–C) of proline (collagen) + $\delta_{op}$(CH) of tannins | (Cárcamo et al., 2012; Espina et al., 2022; Malea et al., 2024; Mandair & Morris, 2015) |
| - | - | 873 | ν(C–C) in hydroxyproline and glycine (collagen) + $\delta_{op}$(CH) of tannins | (Cárcamo et al., 2012; Espina et al., 2022; Malea et al., 2024; Mandair & Morris, 2015) |
| 900 | - | 901 | $\delta_{op}$(CH) of syringic acid | (Espina et al., 2022) |
| 977 | - | 973 | $\nu_1$ (symmetric stretching) $SO_4^{2-}$ + ν(C–COOH) of gallic acid | (Barashkov et al., 2004; Espina et al., 2022) |
| 1003 | 1000 | 1001 | $\nu_1$ (symmetric stretching) $SO_4^{2-}$ + $\nu_1$ (symmetric stretching) $CO_3^{2-}$+ ν(C–C) of phenilalanine (collagen) + δ(CH), ν(ring), 18a of pyrogallol | (Barashkov et al., 2004; Cárcamo et al., 2012; Espina et al., 2022; Malea et al., 2024; Mandair & Morris, 2015) |
| 1036 | 1018 | 1029 | ν(C–N) of proline (collagen) + δ(CH), ν(ring), 18a of tannins | (Cárcamo et al., 2012; Espina et al., 2022; Malea et al., 2024; Mandair & Morris, 2015) |
| 1089 | - | 1092 | $\nu_3$ (symmetric stretching) $SO_4^{2-}$ + δ(NCH) of proline (collagen) + δ(CH), 9b of tannins | (Barashkov et al., 2004; Cárcamo et al., 2012; Espina et al., 2022; Malea et al., 2024; Mandair & Morris, 2015) |
| 1122 | 1137 | 1136 | δ(CH), ν(CO) of pyrogallol and syringic acid | (Espina et al., 2022) |

| | | | | |
|---|---|---|---|---|
| - | 1167 | 1172 | ω($CH_2$), τ($NH_2$), ϱ($NH_3^+$), hydroxylysine (collagen) + δ(CH), ν(CO) of tannins | (Cárcamo et al., 2012; Espina et al., 2022; Malea et al., 2024; Mandair & Morris, 2015) |
| 1193 | - | 1192 | δ(CH), δ(OH) of tannins | (Espina et al., 2022) |
| - | - | 1303 | δ(NH), δ(CH) of collagen | (Cárcamo et al., 2012; Malea et al., 2024; Mandair & Morris, 2015) |
| 1330 | 1326 | 1339 | δ(NH), δ(CH) of collagen | (Cárcamo et al., 2012; Malea et al., 2024; Mandair & Morris, 2015) |
| 1386 | - | 1371 | ω($CH_2$) of glycine (collagen) + δ(CH), δ(OH), δ(C–OH), ν(ring) of tannins | (Cárcamo et al., 2012; Espina et al., 2022; Malea et al., 2024; Mandair & Morris, 2015) |
| 1471 | - | 1463 | $\nu_{as}$($COO^-$) of lysine (collagen) + ν(ring), δ(OH), 19a of tannins | (Cárcamo et al., 2012; Espina et al., 2022; Malea et al., 2024; Mandair & Morris, 2015) |
| - | - | 1558 | ν(ring), δ(C–OH), δ(CH), 19b of tannins | (Espina et al., 2022) |
| - | - | 1582 | ν(ring), δ(OH), δ(CH), 8a of tannins | (Espina et al., 2022) |
| 1601 | - | 1622 | δ(C=C) of phenilalanine and tyrosine (collagen) + ν(ring), δ(OH), δ(CH), 8a of tannins | (Cárcamo et al., 2012; Espina et al., 2022; Malea et al., 2024; Mandair & Morris, 2015) |
| - | - | 1654 | Amide I, ν(C=O) of collagen | (Cárcamo et al., 2012; Malea et al., 2024; Mandair & Morris, 2015) |
| 1787 | - | 1781 | ν(C=O) stretching of free carbonyl groups and/or overtone of Amide I in collagen | (Espina et al., 2022) |
| 1847 | - | 1833 | O–D···$SO_4^{2-}$ | (Avakyants et al., 2025) |
| 2163 | - | 1984 | O–D···$SO_4^{2-}$ | (Avakyants et al., 2025) |
| | - | 2466 | O–D···$SO_4^{2-}$ | (Avakyants et al., 2025) |
| 2735<br>2891 | - | 2896 | $\nu_s$($CH_2$) of collagen and tannins + $\nu_s$/$\nu_{as}$(O–$CH_3$) of methoxy groups (tannins) + contribution of νO-H (water) | (Espina et al., 2022; Kolesov, 2006; Tenne et al., 2000) |

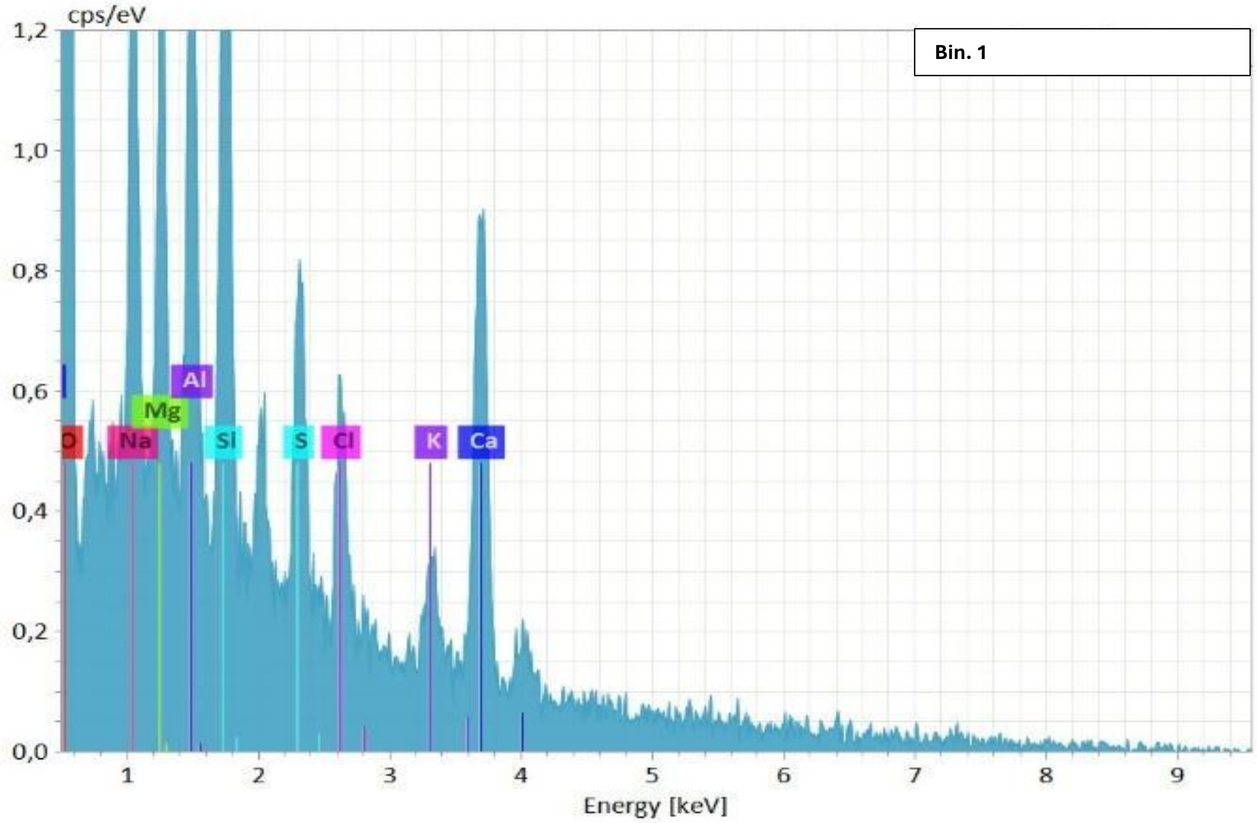


**Figure S3.** EDX spectrum of Bin. 1 sample

**Table S6.** Comparison: Nanocollagen vs. Traditional Klucel G: a comparison of sensor devices (portable and non-portable) to evaluate and monitor the performances of novel consolidating agents versus traditional ones.

| **Property / Parameter** | ***Nanocollagen*** | ***Klucel G*** |
|---|---|---|
| Mechanical Reinforcement<br>Devices that become part of the integrated sensor platform | Excellent; significantly improves tensile strength, elasticity, and resistance to mechanical stress. | Poor/Limited; provides insufficient structural reinforcement or fails to sustain long-term mechanical stress. |
| Shrinkage/Contraction Control | High efficiency; minimal contraction during curing/drying, maintaining substrate dimensional stability. | Low efficiency; prone to shrinkage or poor dimensional stability over time. |
| Colorimetric Properties (CIElab)<br>Devices that become part of the integrated sensor platform (in this specific case, portable/mobile ones as well) | Excellent optical compatibility; negligible color change or below human perception threshold. No yellowing. | Suboptimal; potential for slight color alteration, gloss modification, or yellowing upon aging. |
| Overall Assessment | Highly recommended for applications requiring simultaneous mechanical strengthening and strict aesthetic preservation. | Not recommended when high mechanical reinforcement and optical purity are required. |

**Table S7.** ($I_{CE}$) % index and all the other parameters to evbaluate the consolidation efficiency in the case of the original Bin. 1 sample and the laboratory one sample, called IA_2g/L. As explained in the text, this specific sample was selected from among the laboratory samples because it yields a greater amount of scientifically interesting and useful information following treatment (with Nano-collagen and Klucel G, respectively) and comparison with the laboratory control.

| **Time Zero** | | | | **After 2 years** | | | |
|---|---|---|---|---|---|---|---|
| **Bin. 1 original sample** | | | | | | | |
| $E_{Cohesion}$ | $E_{Elasticity}$ | Color (ΔE) | $I_{CE}$ (%) | $E_{Cohesion}$ | $E_{Elasticity}$ | Color (ΔE) | $I_{CE}$ (%) |
| 90% | 95% | 1.2 | 93.5 | 88% | 85% | 1.8 | 85.9 |
| **IA_2g/L laboratory sample** | | | | | | | |
| $E_{Cohesion}$ | $E_{Elasticity}$ | Color (ΔE) | $I_{CE}$ (%) | $E_{Cohesion}$ | $E_{Elasticity}$ | Color (ΔE) | $I_{CE}$ (%) |
| 89% | 94% | 1.1 | 93.3 | 87% | 84% | 1.6 | 85.7 |

The lower ΔE value (albeit only slightly so) observed for the laboratory sample—using both consolidating agents tested in this study—can be attributed to a few key factors:

- Lower porosity or controlled degradation. The combined treatment (low-concentration acid plus thermal shock) simulates aging but may not have replicated the deep micro-cracking and structural oxidation found in actual historical leather. Consequently, the consolidant penetrates more uniformly and undergoes fewer physicochemical alterations.
- Interaction with the consolidant. The lower ΔE \) value in the artificial sample demonstrates that the consolidating agent does not significantly alter the color of the laboratory-treated material. In contrast, when applied to naturally aged leather—which already possesses a chemical composition altered by dust, light, and actual pollutants—the consolidant reacts in a way that causes a slightly more pronounced color shift.
- Long-term stability (2-year follow-up). The fact that the trend remains stable after two years confirms the long-term effectiveness of the consolidation on both types of samples; however, it also highlights that laboratory-based accelerated aging models tend to be "optimistic"—or less severe—compared to the complex degradation processes occurring in reality.

**Table S8.** Data from ΔC* and ΔH* for the more extensive CIELab study.

| Samples | ΔE | | ΔC* | | ΔH* | |
|---|---|---|---|---|---|---|
| | Nanocollagen | Klucel | Nanocollagen | Klucel | Nanocollagen | Klucel |
| IA_2g/L | 2.49 | 5.22 | 1.20 | 2.32 | 0.50 | 1.09 |
| IA_10g/L | 2.30 | 2.48 | 1.30 | 1.53 | 0.57 | 1.34 |
| INA_2g/L | 1.93 | 1.34 | 1.12 | 1.40 | 0.25 | 1.11 |
| INA_10g/L | 1.67 | 1.95 | 1.28 | 1.47 | 0.33 | 1.26 |
| Bin. 1 | 2.80 | 2.17 | 1.29 | 1.47 | 0.28 | 1.19 |
| Bin. 5 | 1.62 | 3.36 | 1.24 | 1.39 | 0.22 | 1.12 |
| Bin. 6 | 2.27 | 1.12 | 1.29 | 1.45 | 0.48 | 1.31 |

In the comprehensive study of colorimetry applied to conservation treatments for aged leather (particularly leather subjected to acid hydrolysis and thermal stress, according to the laboratory samples preparation), the distribution of ΔE follows specific physicochemical dynamics: ΔL (Lightness) is generally the parameter that undergoes the greatest change. Traditional polymeric consolidants, such as Klucel G, tend to penetrate the pores and alter the surface's refractive index, often causing a slight "wet" look or darkening (corresponding to a lower ΔL values, see Table 6 and 7 in the full text). Nanocollagen, possessing a matrix structurally similar to that of leather, aims to minimize this inherent optical alteration, thereby maintaining a more limited ΔL.

In the colorimetric tables from this study, it is to be expected that the dominant component of the ΔE value to be accounted for by ΔL. Consequently, for both samples, ΔH will be very low especially for Nanocollagen, confirming that the new nanostructured consolidating agent does not significantly alter the leather's original hue (for the traditional Klucel G product, the values are higher). ΔCwill show low (in presence of Nanocollagen)-to-moderate intensity values (for Klucel G based treatments), tracking the change in saturation induced by the consolidating film.

In light of this addition regarding the extended CIELab study, it can be concluded that parameters such as ΔC and ΔH were omitted from the original version of the manuscript; this is because ΔE is primarily linked to ΔL, and ΔE itself represents the most significant CIELab parameter within the algorithm used to quantitatively determine Consolidation Efficiency ($I_{CE}$%).
